# Genomic‐Based Epidemiological Analysis of the Post‐Pandemic *Mycoplasma pneumoniae* Resurgence

**DOI:** 10.1002/mco2.70617

**Published:** 2026-01-22

**Authors:** Hongbo Liu, Xiaoyi Zheng, Xinying Du, Yule Wang, Ying Xiang, Qi Wang, Sai Tian, Yufan Xian, Wenbin Chen, Hongbo Liu, Hui Wang, Chao Wang, Mingjuan Yang, Huiqun Jia, Xiaoying Li, Yunjie Dan, Libo Tong, Guohong Deng, Huiling Li, Fusheng Wang, Hongbin Song, Shaofu Qiu

**Affiliations:** ^1^ Chinese PLA Center for Disease Control and Prevention Beijing China; ^2^ College of Life Science and Technology Beijing University of Chemical Technology Beijing China; ^3^ Department of Infectious Diseases Southwest Hospital Third Military Medical University (Army Medical University) Chongqing China; ^4^ The 960th Hospital of the PLA Joint Logistics Support Force Jinan China; ^5^ Yu‐Yue Center for Pathology Research Chongqing China; ^6^ Hainan Hospital of Chinese People's Liberation Army General Hospital Sanya China; ^7^ Senior Department of Infectious Diseases The Fifth Medical Center of Chinese PLA General Hospital Beijing China

**Keywords:** macrolide resistance, mycoplasma pneumoniae, phylogenomic analysis, resurgence, targeted next‐generation sequencing

## Abstract

*Mycoplasma pneumoniae* infections resurged globally in 2023–2024 following a significant decline during the coronavirus disease 2019 (COVID‐19) pandemic. To understand the genomic epidemiology of this resurgence in China, a nationwide 1‐year genomic surveillance identified 9907 patients infected with *M. pneumoniae*, resulting in an overall positive rate of 10.05%. We developed a hybrid capture‐based targeted next‐generation sequencing (hc‐tNGS) assay, obtaining 271 high‐quality genomes directly from clinical samples. Phylogenetic analysis of a global collection of 562 *M. pneumoniae* genomes identified six distinct lineages, including three newly emerged main Chinese clades (MCCs) that co‐circulated across various regions of China. Among these MCCs, one clade, comprising P1‐1, ST17, and L4, was localized in Taiwan, while two others—P1‐1, ST3, and L6 clade, and P1‐2, ST14 and L2 clade—co‐circulated in different regions of China during the 2023–2024 epidemic season. Notably, 96.31% of the isolates identified in this study exhibited a point mutation, primarily A2063G (95.94%). This study offers a comprehensive genomic characterization of the post‐pandemic *M. pneumoniae* resurgence in China, highlighting the emergence and spread of resistant clades. These findings emphasize the importance of adopting a One Health approach to address the potential global public health threats posed by this resurgent pathogen.

## Graphical abstract

Phylogenetic analysis of a global collection of 562 *M. pneumoniae* genomes revealed six distinct lineages, including three newly emerged main Chinese clades (MCCs) that co‐circulated in various regions of China. The recent emergence of these distinct clades and their differing geographical distributions may suggest ongoing divergent evolution within the species or indicate specific transmission patterns.

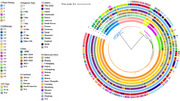

## 1 Introduction

*Mycoplasma pneumoniae*, one of the smallest prokaryotic organisms lacking a rigid cell wall, frequently causes respiratory tract infections (RTIs) in both children and adults [1]. The clinical manifestations of *M. pneumoniae* infection vary widely, ranging from self‐limiting to severe pneumonia and even life‐threatening disease with extrapulmonary complications [2]. *M. pneumoniae* may be responsible for approximately 4%–8% of community‐acquired pneumonia (CAP) cases in endemic settings, and can cause up to 20%–40% of CAP cases in the general population during epidemics, rising to up to 70% in closed populations [3]. A global survey reported an 8.61% incidence of *M. pneumoniae* infection across all age groups from 2017 to 2020 [4]. According to the multinational Asian Surveillance Network for Drug‐Resistant Pathogens (ANSORP), *M. pneumoniae* accounts for 11% of CAP cases in adults across eight Asian countries [5]. In China, it is responsible for 10%–30% of childhood pneumonia cases, surging to 30%–50% during epidemic peak years [6, 7]. Thus, *M. pneumoniae* is a leading cause of RTIs and CAP worldwide, posing a significant threat to global public health.

Macrolides are recommended as the first‐line antibiotics for the treatment of *M. pneumoniae* infections, as alternative antibiotics such as fluoroquinolones and tetracyclines may pose potential toxicities in children [8]. With the extensive use of macrolides, a macrolide‐resistant *M. pneumoniae* (MRMP) strain was first reported in Japan in 2000 [7, 9, 10]. This resistance is mainly due to point mutations in the 23S rRNA gene, such as A2063G and A2064G in domain V, which decrease macrolide binding affinity [10]. Since then, MRMP strains have become increasingly prevalent worldwide, especially in Asia [7, 11]. The prevalence of MRMP varies widely around the world, with prevalence rates ranging from 1% to 30% in the United States and Europe to 80%–90% in Asia [12, 13]. MRMP infections can result in longer duration of fever and hospitalization compared to macrolide‐sensitive infections, leading to more patients being switched to alternative antibiotics [14]. The high prevalence of MRMP in Asia, combined with limited treatment options for children, presents a significant clinical challenge, making empirical treatment more difficult and often ineffective. Consequently, the rise and spread of MRMP complicate the clinical management of *M. pneumoniae* infections, particularly in children, and pose a serious public health threat. This growing resistance crisis highlights the urgent need for comprehensive surveillance to track the evolution and spread of resistant strains.

Beyond antimicrobial resistance, *M. pneumoniae* is characterized by cyclic epidemics occurring every 3–7 years, with each outbreak lasting 1–2 years [15, 16, 17]. The latest epidemic emerged in late 2019 and early 2020, affecting several countries, primarily in Europe and Asia [1, 4, 18, 19]. However, after the implementation of non‐pharmaceutical interventions (NPIs) against the coronavirus disease 2019 (COVID‐19) from 2020, such as mask wearing, hand hygiene, social distancing, travel restrictions, and school closures, *M. pneumoniae* positive rates decreased dramatically in diverse countries in Asia, Oceania, the Americas, and Europe during the pandemic [1, 4]. This suppression significantly reduced population immunity, potentially enlarging the pool of susceptible individuals, particularly young children who had not been previously exposed. With the lifting of social quarantine restrictions, an increase in the incidence of RTIs among children in northern China has been reported since mid‐October 2023, attributed to circulating known pathogens including *M. pneumoniae* [8, 20, 21]. Concurrently, a resurgence of *M. pneumoniae*‐related respiratory infections has been observed in other countries worldwide, predominantly affecting children and adolescents [1, 8, 21, 22]. This atypical epidemic of *M. pneumoniae* is noteworthy and has garnered widespread attention, warranting further investigation into the reasons behind this unusual resurgence.

Given the high burden of *M. pneumoniae* infection recently, a timely study is urgently needed to clarify the current epidemic characteristics and risk factors with emerging trends. Since *M. pneumoniae* infection is not a nationally notifiable disease and lacks an organized surveillance program in China and most other countries, comprehensive epidemiological data on remain scarce, although many single‐center studies have been reported [6, 7, 14, 15, 17]. The absence of a coordinated surveillance system has led to fragmented and often delayed insights, leaving public health authorities with an incomplete picture for effective interventions guidance. The factors contributing to the unexpected resurgence of *M. pneumoniae* infection are still under investigation. It is crucial to determine whether this resurgence is driven by the waning of NPIs, the natural epidemic cycle, the emergence of novel genetic lineages with enhanced fitness, or a combination of these factors to enhance future public health preparedness. Therefore, in this study, we conducted a nationwide prospective surveillance of *M. pneumoniae* epidemiology in China from November 2023 to December 2024. Additionally, *M. pneumoniae* is notoriously difficult to culture, making it challenging to obtain whole genomes, which results in limited understanding of its genotypes, lineages, macrolide resistance, transmission, and genomic variations. To address this, we developed a culture‐independent method using hybrid capture‐based targeted next‐generation sequencing (hc‐tNGS) to sequence whole genomes directly from clinical samples. This approach aims to determine the prevalence, transmission, and phylogeny of *M. pneumoniae*, as well as genomic features potentially linked to its resurgence.

## 2 Results

### 2.1 Demographic and Epidemiological Characteristics of Patients With *M. pneumoniae* Infections

**FIGURE 1:**
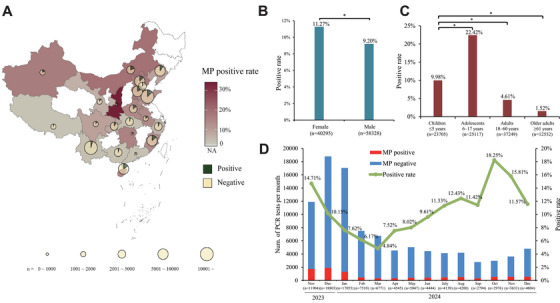
The epidemiology *M. pneumoniae* in China. (A) The study sites and MP positive rate in different provinces of China. The map approval number is GS(2019)1819. (B) MP positive rate in male and female. (C) MP positive rate in different age groups. (D) The monthly number of PCR tests from November 2023 to December 2024. *p < 0.0001.

During the study period from November 1, 2023, to December 31, 2024, a total of 98,623 specimens were collected from patients with ARTI. Among these, 9907 patients (10.05%) from 43 hospitals across 24 provinces were confirmed to have *M. pneumoniae* infections via RT‐PCR (Table S1). The PCR‐positive rate in males and females was 9.20% and 11.27%, respectively (Figure 1B). The highest PCR‐positive rate was observed in individuals aged 6–17 years (22.42%), followed by those aged ≤ 5 years (9.98%) (Figure 1C). The incidence of positive cases decreased from 14.71% in November 2023 to 4.84% in March 2024, peaking at 18.25% in October 2024 (Figure 1D). Of the 9907 positive cases, 5367 (54.17%) were males and 4540 (45.83%) were females, resulting in a gender ratio of 1.18:1. The median age was 8 years (range, 0–95 years), with the majority of patients aged 6–17 years (56.85%), followed by those aged ≤ 5 years (23.88%), 18–60 years (17.34%), and ≥ 61 years (1.93%). Clinically, 5501 patients (55.53%) exhibited symptoms of upper respiratory tract infection, 524 (5.29%) developed bronchitis, and 1975 (19.94%) manifested pneumonia (Table 1).

**TABLE 1:** Demographic and clinical characteristics of *M. Pneumoniae* infections in mainland China, 2023–2024.

	MP positive (*n* = 9907)^a^
Sex, *n* (%)	
Female	4540 (45.83%)
Male	5367 (54.17%)
Age, years, median (IQR)	8 (0–95)
Age group, *n* (%)	
Children,≤ 5 years	2366 (23.88%)
Adolescents, 6–17 years	5632 (56.85%)
Adults, 18–60 years	1718 (17.34%)
Older adults, ≥ 61 years	191 (1.93%)
Clinical diagnosis, *n* (%)	
Pneumonia	1975 (19.94%)
Bronchitis	524 (5.29%)
Upper respiratory tract infection	5501 (55.53%)
Not available	2012 (20.31%)

^a^ For clinical diagnosis, percentages do not sum to 100% due to composite diagnoses, where patients may have been diagnosed with more than one condition simultaneously.

### 2.2 Evaluation of Whole‐Genome Sequencing Methods for *M. pneumoniae*

**FIGURE 2:**
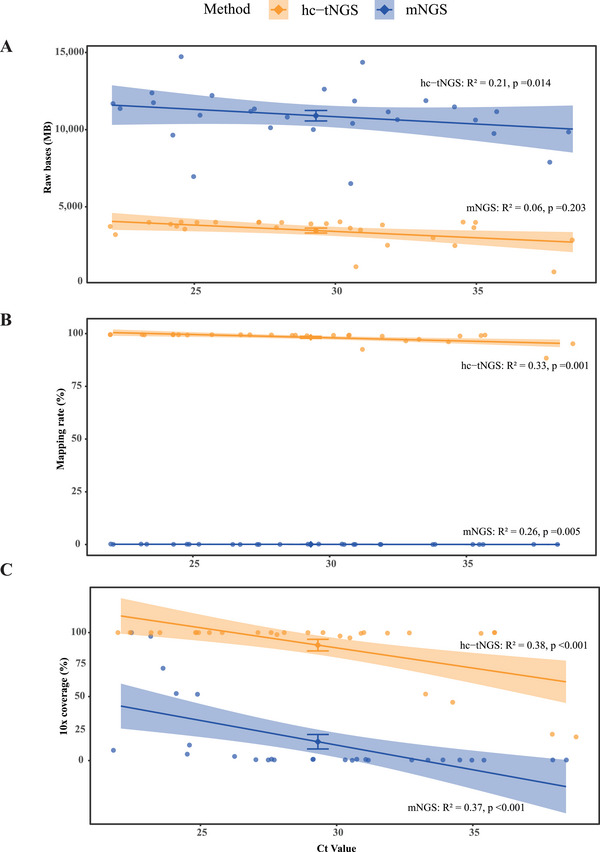
*M. pneumoniae* rawbases, mapping rate and 10 × genome coverage obtained using mNGS and hc‐tNGS of MP‐positive samples with different Ct values. (A) Rawbases generated by mNGS and hc‐tNGS. (B) Mapping rate obtained when aligned to the *M. pneumoniae* reference genome. (C) Genome coverage with depth ≥ 10. Data are presented as individual points (jittered) with mean ± standard error (SEM, *n* = 28). Trend lines represent linear regression with 95% confidence intervals.

In this study, we selected 28 representative *M. pneumoniae* positive samples with varying Ct values (range, 22.08–38.42) to assess the efficiency of whole‐genome sequencing using metagenomic next‐generation sequencing (mNGS) and hc‐tNGS. The median raw bases (Mb) generated were 11,156.37 (range, 6,504.18–14,725.89) for mNGS and 3780.37 (range, 781.42–4022.4) for hc‐tNGS (Figure 2A, Table S2). Despite producing a larger data volume, mNGS lagged behind hc‐tNGS in mapping rate. When aligned to the *M. pneumoniae* reference genome, hc‐tNGS exhibited a median mapping rate of 99.27% (range, 88.37%–99.61%), significantly higher than mNGS's median mapping rate of 0.06% (range, 0.01%–0.22%) (Figure 2B, Table S2). Consequently, hc‐tNGS achieved superior genome coverage at 10x depth compared to mNGS. Additionally, sequencing quality, particularly genome coverage, appeared strongly linked to the pathogen load in the samples. Only two samples with Ct values of 22.08 and 23.56 sequenced by mNGS showed better sequencing data quality, whereas samples with Ct values ≤ 32 sequenced by hc‐tNGS demonstrated superior data quality (Figure 2C, Table S2). Therefore, hc‐tNGS proved more efficient than mNGS, prompting us to use hc‐tNGS for sequencing whole genomes of selected samples with Ct values ≤ 32, avoiding those with a low likelihood of positivity.

### 2.3 Phylogenetic Analysis of Global *M. pneumoniae* Collections Including Chinese Isolates

**FIGURE 3:**
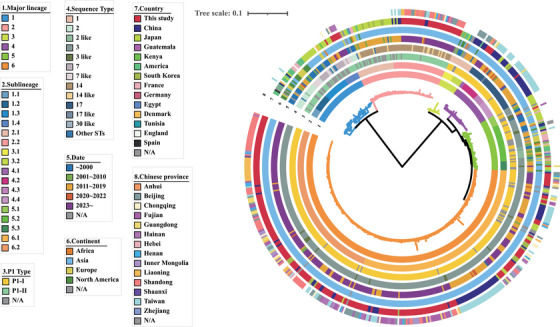
Population structure of the global *M. pneumoniae* collections in this study. Maximum likelihood phylogeny was performed on 562 global *M. pneumoniae* genomes, including the 271 Chinese strains in this study and 291 public genomes. The population structure was estimated using hierBAPS, and lineages or sublineages are labeled with different colors as LX or LX.Y, where X and Y are the lineage and sublineage numbers, respectively. The rings, from inner to outer, labeled with different colors indicate the lineages, sublineages, P1 genotypes, sequence types, isolation date, continent, country, and Chinese province.

In this study, we selected 294 samples with Ct values ≤ 32 for whole‐genome sequencing using the hc‐tNGS method and obtained 271 genomes with sequencing depth ≥ 10x and coverage ≥ 99%, representing various regions of China (Table S3, Figure S1). We then constructed a global genome dataset comprising 562 *M. pneumoniae* strains, which included 271 genomes from this study and 291 genomes downloaded from NCBI GenBank (Table S3, Figure S1). A maximum likelihood (ML) phylogenetic tree was constructed based on 3879 non‐repetitive core SNPs identified from these 562 genomes. The global collection was divided into six distinct lineages (lineages L1 to L6) and further subdivided into 17 sublineages through hierBAPS analysis, with L6 being the largest lineage (*n* = 318, 56.58%) and L3 the smallest (*n* = 18, 3.2%) (Figure 3). P1 genotyping classified 389 isolates (69.22%) as P1‐1 and 88 (15.66%) as P1‐2, while 85 isolates (15.12%) could not be definitively classified. However, as shown in Figure 3, among the 85 unclassified isolates, 47 were distributed within the P1‐2 genotype and 38 within the P1‐1 genotype. Isolates in lineages L1‐L2 were identified as P1‐2, whereas those in lineages L3‐L6 were identified as P1‐1. Based on these analyses, we concluded that all isolates in lineages L1‐L2 belonged to P1‐2, while those in lineages L3‐L6 belonged to P1‐1. Multilocus sequence typing (MLST) analysis further divided the global *M. pneumoniae* populations into 18 sequence types (STs), with ST3 (*n* = 342, 60.85%) being the most prevalent, followed by ST14 (*n* = 60, 10.68%), ST17 (*n* = 40, 7.12%), ST2 (*n* = 26, 4.63%), ST1 (*n* = 18, 3.20%) and ST7 (*n* = 17, 3.02%). Isolates in lineages L5–L6 were mostly composed of ST3, those in lineage L1 were mainly ST2 and ST7, while lineages L2–L4 consisted of ST14, ST1, and ST17, respectively.

Among the six lineages, L1–L3 and L5 showed a global distribution, while L2, L4, and L6 consisted solely of strains from Asia, specifically China, Japan, and South Korea (Figure 3). Strains from North America and Europe were primarily found in lineages L1 and L5, with North American strains appearing ancestral to current *M. pneumoniae* populations. Asian strains, particularly from Japan and China, were spread across all six lineages. Japanese strains were mainly in lineages L2 (*n* = 23, 30.26%), L1 (*n* = 20, 26.32%), L5 (*n* = 17, 22.37%), and L6 (*n* = 12, 15.79%). In contrast, Chinese strains were predominantly in L6 (*n* = 283, 73.70%), L2 (*n* = 43, 11.20%), and L4 (*n* = 36, 9.38%), forming three main Chinese clades (MCCs). These included a distinct Taiwanese clade (L4) and two MCCs in L2 and L6 that co‐circulated in various regions of mainland China (Figure 3, Figure S2). Additionally, four isolates from Hainan, two from Chongqing, and one from Beijing clustered within sublineage L1.4 alongside six isolates from Japan (Figure 3). Furthermore, Chinese strains exhibited a closer relationship to Japanese strains within each lineage. These findings suggest that ancestors from North America and Japan may significantly influence the prevalence and transmission of contemporary *M. pneumoniae* populations. The locally established and prevalent Chinese MCCs likely trace back to Japanese ancestors and may have been introduced into China through multiple transmission events.

As shown in Figure 3, earlier isolated *M. pneumoniae* strains are generally located at the root of each phylogenetic lineage, whereas the newly sequenced genomes from this study often cluster at the base, forming emerging epidemic clades. After evaluating various BEAST settings, we identified GTR+F+Γ/UCED as the optimal configuration (Table S4). By removing aberrant temporal signals through time regression (Figure S3), Bayesian evolutionary analysis estimated that the P1‐1 population is descended from an MRCA that existed circa 1887 (95% confidence interval [CI]: 1812–1941), and the MRCA of the P1‐2 population may have emerged circa 1850 (95% CI: 1731–1927). Lineages L2 and L6 appear to have diverged around 1968 (95% CI: 1949–1982) and 1977 (95% CI: 1971–1980), respectively. These two lineages experienced rapid expansion in 1980 and 2000, respectively (Figure S4), suggesting they may have evolved into recently emerged clones undergoing contemporary localized clonal expansion, which could have significant public health implications.

### 2.4 Macrolide Resistance of Global *M. pneumoniae* Collections

**FIGURE 4:**
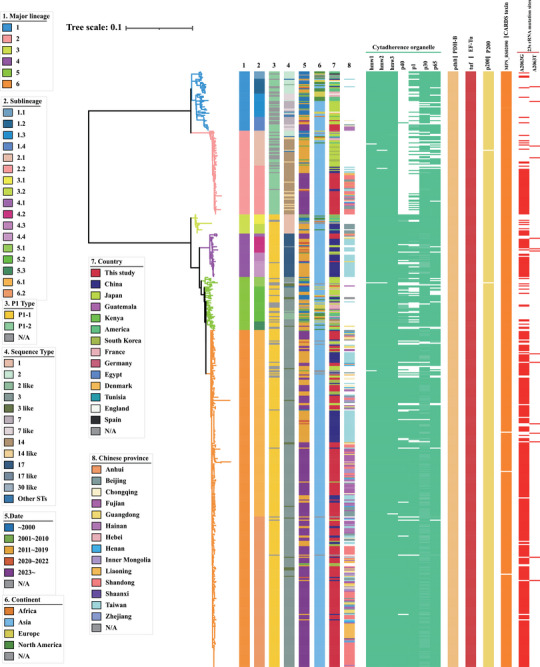
Phylogeny of the global *M. pneumoniae* collections with a heatmap showing the distribution of virulence factors and point mutations of 23S rRNA. The different bands at the left of the heatmap indicate the information of the global *M. pneumoniae* collections. The stripes marked in different colors represent the distribution of different types of virulence genes, and the point mutations of 23S rRNA, particularly the A2063G and A2063T, are separately indicated in red color.

Mutations in the V domain of the 23S rRNA of *M. pneumoniae* can influence macrolide resistance phenotypes. We examined point mutations in this domain and found that 73.31% of global *M. pneumoniae* samples carried a mutation. The most prevalent was A2063G (70.11%), followed by A2063T (2.49%), A2063C (0.18%), A2064C (0.18%), A2064G (0.18%), and C2617G (0.18%) (Figure 4; Figure S5). Isolates from Asia, especially China, exhibited a higher frequency of mutations compared to other regions. Specifically, 96.31% of Chinese isolates in our study had a mutation, predominantly A2063G (95.94%). Moreover, isolates of the P1‐1 genotype showed a higher mutation rate than those of the P1‐2 genotype. However, almost all Chinese isolates in lineage L2 belonging to the P1‐2 genotype and those in L4 and L6 belonging to the P1‐1 genotype contained the A2063G point mutation (Figure 4). Furthermore, the proportion of global, Asian, and Chinese collections with these mutations has increased over time (Figure S5). These findings underscore the emergence of three distinct Chinese clades (L2, L4, and L6) with macrolide resistance, suggesting that the resistant MCCs (L2 and L6) may have evolved from macrolide‐susceptible strains.

### 2.5 Virulence Factors of Global *M. pneumoniae* Collections

A total of 11 virulence factors were identified from the VFDB database. The majority of global *M. pneumoniae* collections included the key virulence factors, especially those linked to the cytadherence organelle and the community‐acquired respiratory distress syndrome (CARDS) toxin. Notably, the p40 gene was primarily found in isolates of the P1‐1 genotype, while it was almost absent in those of the P1‐2 genotype (Figure 4).

### 2.6 Pangenome Analysis

**FIGURE 5:**
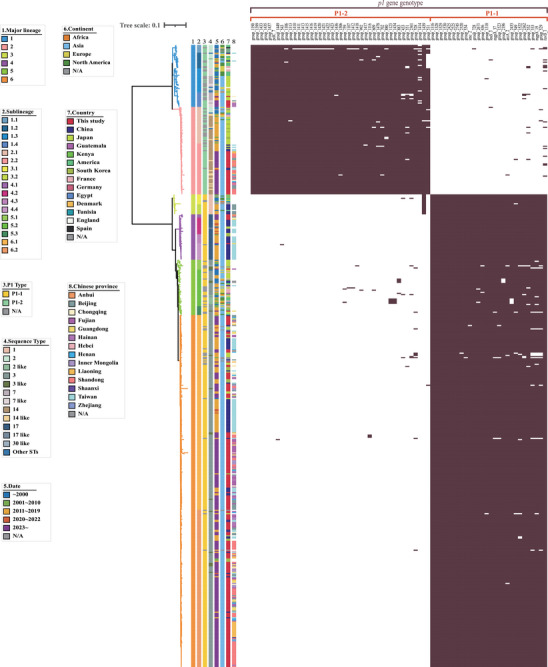
Accessory genes identified by pangenome analysis that might be significantly associated with P1 genotypes of *M. pneumoniae*. A total of 71 specific genes that are significantly associated with the P1 genotypes are screened by the chi‐square test whose *p* value was lower than 1e‐100.

The pangenome analysis of 562 isolates revealed a total of 3053 genes, comprising 1091 core genes, 105 soft core genes, 311 shell genes, and 1546 cloud genes (Figure S6). Among the accessory genes, 71 specific genes were identified as significantly associated with the P1 genotypes with *p* values below 1e‐100. Of the specific genes, the majority (91.55%) were hypothetical protein genes, with 28 genes found in the P1‐1 genotype and 43 in the P1‐2 genotype (Figure 5, Table S5).

## 3 Discussion and Conclusion

In this study, we conducted a nationwide, 1‐year prospective epidemiological surveillance of *M. pneumoniae* in China, highlighting the resurgence of *M. pneumoniae* infections during the 2023–2024 season. The most recent *M. pneumoniae* epidemic occurred simultaneously in several countries, primarily in Europe and Asia, during the 2019–2020 cold season, just before the COVID‐19 pandemic began [1, 4]. During the 2020–2022 COVID‐19 pandemic, widespread adoption of NPIs led to a dramatic reduction in *M. pneumoniae* prevalence across Asia, Oceania, the Americas, and Europe [4, 11, 23]. A national surveillance study in China showed that the positive rates of most pathogens tested significantly decreased in 2020 compared to the 2009–2019 period, with *M. pneumoniae* showing the largest drop [23]. Following the COVID‐19 pandemic, a re‐emergence of *M. pneumoniae* was observed in China and other countries from autumn 2023 [1, 8, 11, 18, 22, 24]. Our study demonstrated a large‐scale, nationwide epidemic of *M. pneumoniae* in China during the 2023–2024 season. These analyses indicated a global resurgence of *M. pneumoniae* infections in 2023–2024 after a 3‐year decline during the COVID‐19 pandemic. The decreased prevalence of *M. pneumoniae* infections likely led to the accumulation of a susceptible population and a subsequent population immunity debt due to long‐term NPIs implemented during the COVID‐19 pandemic, resulting in *M. pneumoniae* epidemics after the COVID‐19 pandemic [25]. Such long‐term NPIs have also caused the resurgence of other respiratory pathogens such as *respiratory syncytial virus*, *influenza virus*, *enterovirus*, *Streptococcus pneumoniae*, and *Group A Streptococcus* due to immunity debt [16, 26]. Additionally, the resurgence of *M. pneumoniae* in China was mainly driven by MRMP strains, differing from other countries. For instance, the increased incidence of *M. pneumoniae* in Denmark in 2023–2024 was not primarily due to MRMP strains, as macrolide resistance was detected in less than 2% of samples tested [22, 26]. This finding suggests that the emergence and transmission of MRMP strains is not the main reason for the current global resurgence of *M. pneumoniae* infections after the COVID‐19 pandemic.

*M. pneumoniae* infections are typically identified using serological or molecular methods. Although *M. pneumoniae* culture is the gold standard for clinical diagnosis, it is unsuitable for rapid diagnosis due to its specific culture requirements and slow growth. Serological tests and PCR‐based molecular methods, such as RT‐PCR, offer rapid and cost‐effective diagnosis of *M. pneumoniae* due to their high sensitivity and specificity [26]. However, these traditional methods fall short in monitoring antimicrobial resistance, genotypes, lineages, sources, transmission, and genomic variation of *M. pneumoniae*. To enhance prevention and treatment, developing cost‐effective, high‐quality whole‐genome sequencing strategies for *M. pneumoniae* is essential. Currently, mNGS sequencing directly from clinical samples and culture‐dependent NGS are the primary strategies for whole‐genome sequencing of clinically relevant microorganisms, including *M. pneumoniae*. Nevertheless, challenges such as high human host nucleic acid background, difficulty in culturing, and high costs hinder the clinical application and suitability of NGS in various scenarios. In this context, targeted NGS (tNGS) has emerged as a cost‐effective alternative to mNGS [27, 28, 29, 30]. Here, we developed a rapid, accurate, cost‐effective, and culture‐free tNGS for whole‐genome sequencing of *M. pneumoniae*. For the first time, we obtained 271 high‐quality genomes of *M. pneumoniae* directly from 294 clinical samples using hc‐tNGS, which reliably generates unbiased coverage of the entire *M. pneumoniae* genome. These genomes are valuable for subsequent genomic epidemiological studies, including macrolide resistance mutations, genotyping, phylogeny, and genomic variation analyses, demonstrating superior sequencing quality of hc‐tNGS compared to mNGS presented in other studies [20, 24]. These findings suggest that culture‐free tNGS can provide accurate sequencing results for detecting and characterizing clinically relevant microorganisms, with significant potential to aid clinical decision‐making. A practical consideration for applying this approach is that obtaining high‐quality genomes, as demonstrated in this study, is currently most reliable from samples with lower Ct values (≤ 32), which may under‐represent lineages associated with very low bacterial loads.

The rise of MRMP poses significant concerns about treatment failures, the need for alternative antibiotics, and prolonged disease duration, making it a major public health threat with far‐reaching implications. Due to the difficulty in culturing *M. pneumoniae*, detecting macrolide resistance is challenging. Previous research has strongly linked point mutations in the 23S rRNA gene of *M. pneumoniae* to macrolide resistance phenotypes. Specifically, mutations at positions 2063 and 2064 are associated with high resistance levels, while mutations at positions 2067 and 2617 correspond to lower resistance levels [31]. Therefore, we identified point mutations in the 23S rRNA gene to determine macrolide resistance phenotypes. Here, we found that 95.94% of Chinese strains isolated from various regions in 2023 carried the A2063G mutation, indicating high levels of macrolide resistance among current epidemic strains in China. Although clinical outcomes were not assessed, the high prevalence of these mutations suggests widespread macrolide ineffectiveness. Therefore, prospectively defining the clinical impact of these genotypes is crucial for future research. Our present study, together with previous studies, has shown that macrolide resistance varies globally and changes over time. The highest resistance prevalence has been observed in Asia, particularly in China, with lower prevalence in Europe and North America [11, 32]. Since the first MRMP strain was reported in Japan in 2000, MRMP prevalence has rapidly increased worldwide, exceeding 90% in some areas of China and Japan. However, MRMP prevalence has decreased in many regions or countries in recent years, especially during the COVID‐19 pandemic. A systematic review and meta‐analysis showed that global MRMP prevalence has changed over the last 20 years, from 27% before 2008% to 50% in 2009–2011, with peaking at 51% in 2012–2014, then declining to 39% in 2015–2017 and 23% in 2018–2020 [32]. A global survey indicated a decrease in MRMP rates from 23.10% between April 2017 and March 2020 to 4.55% between April 2020 and March 2021 [4]. In Japan, the MRMP detection rate rose to 90% during the 2011–2012 outbreak but fell to 14.3% in 2018 [33]. In Taiwan, MRMP prevalence increased from 12.3% to 24% before 2017 to 54% to 88% during 2017–2020, then decreased to 18.2% in 2021 and 0% in 2022–2023 [33]. Conversely, in China, the prevalence of macrolide resistance remained high before, during, and after the COVID‐19 pandemic and showed an apparent increasing trend over time, with resistance rates exceeding 90% during and after the COVID‐19 pandemic [14, 17, 34]. This sustained high prevalence in China, contrasting sharply with the decline in Japan and Taiwan where strict antibiotic stewardship is in place, likely reflects differences in macrolide usage and antimicrobial management policies. Antibiotic use is complex and widespread, with cephalosporins, macrolides, and fluoroquinolones being among the most commonly used antibiotics in Asia, particularly in China and Japan [35, 36, 37, 38, 39, 40]. As reported by Hsia, azithromycin is the most commonly prescribed antibiotic for hospitalized children in China, Japan, and South Korea, with a prescription rate of 11.8%, higher than in Europe, America, Africa, and Southeast Asia [35]. In vivo and in vitro studies have shown that macrolide resistance‐associated mutations, including A2063G and A2064G, can be induced by macrolide use [3, 41, 42]. Furthermore, as shown in Figure 4, two distinct MCCs (L2 and L6) circulating in different regions of China and a distinct Taiwanese lineage (L4) were observed, all commonly harboring macrolide resistance‐associated mutations, particularly A2063G. These findings suggest that the heavy use of macrolides may have resulted in a sufficiently high and sustained selective pressure for the emergence, spread, and maintenance of MRMPs from diverse genetic backgrounds of *M. pneumoniae*, rather than the clonal spread of a single MRMP strain.

This study offers an overview of the global phylogeny of *M. pneumoniae*, focusing particularly on the newly sequenced Chinese collections within this global context. The global collections were divided into six distinct lineages, with four lineages evident within the P1‐1 genotype and two within the P1‐2 genotype. The P1‐1, ST3, and L6 clade emerged as the predominant genotype within the global *M. pneumoniae* collections, followed by the P1‐2, ST14, and L2 clade as the second most common. Additionally, strains with similar origins, such as isolation time or geographical location, tended to cluster together. We identified a distinct clade, P1‐1, ST17, and L4, localized in Taiwan, along with two MCCs belonging to the above two genotypes that co‐circulated in different regions of China during the 2023–2024 epidemic season. Although the lack of national pre‐2023 genomic baseline data prevents us from completely ruling out sampling influences, the strong geographical clustering observed is more likely due to localized transmission than site selection artifacts, given our surveillance network's extensive geographic coverage across China. The recent emergence of these distinct clades and their geographical distribution differences may indicate ongoing divergent evolution within the species or suggest transmission patterns. It has been speculated that cyclic *M. pneumoniae* epidemics, which tend to occur every few years, may be related to genomic variations (such as genotype shifts from one P1 subtype to another) and changes in human population immunity [3, 18, 26, 43, 44]. Genotype shifts from P1‐1 to another have occurred repeatedly at 10 years intervals. In Japan, it has been reported that clinically prevalent *M. pneumoniae* shifted from P1‐1 to P1‐2 over the last decade [43]. However, the P1 genotype is not always the sole determinant of cyclic outbreaks as co‐circulation of both P1 genotypes and multiple variants has been documented in epidemic and endemic settings [3, 43, 44], as observed in this study. Here, we identified some genetic signatures significantly associated with the P1 genotype, including accessory genes of unknown function and a virulence gene *p40* involved in cytadherence [3]. Most genotype‐associated genes identified in the pangenome analysis were annotated as hypothetical proteins, and their potential biological roles require further exploration. The genotype shift or co‐circulation of both P1 genotypes may alter herd immunity levels, leading to a population immunity debt due to the long‐term NPIs implemented during the COVID‐19 pandemic. However, the absence of pre‐ and post‐pandemic seroprevalence data for *M. pneumoniae* in our study means that the role of immunity debt, while consistent with broader epidemiological trends, remains to be definitively confirmed.

This study has several limitations. First, our present study lacked national epidemiological data on *M. pneumoniae* before and during the COVID‐19 pandemic, making it challenging to analyze its epidemiological characteristics across these periods, despite numerous single‐center studies. Second, we did not culture *M. pneumoniae* strains due to their challenging nature, preventing us from obtaining antimicrobial susceptibility data, but we determined the macrolide resistance phenotype by examining macrolide resistance‐associated mutations from the obtained genomes. Third, the findings of this study should be interpreted in light of its demographic and geographic scope. The cohort is skewed toward children and adolescents, who have the highest infection incidence, potentially limiting the generalizability of our estimates to adult and elderly populations. Furthermore, since all sentinel hospitals were in urban areas and the western region of China was undersampled, the results may not fully represent the epidemiological situation in rural communities or the western part of the country.

In conclusion, we conducted a nationwide, 1‐year prospective epidemiological surveillance of *M. pneumoniae* in China, highlighting the resurgence of *M. pneumoniae* infections during the 2023–2024 season. For the first time, we developed a rapid, accurate, cost‐effective, and culture‐free tNGS method, which allowed us to obtain high‐quality genomes of *M. pneumoniae* for subsequent genomic epidemiological studies. The resurgence of *M. pneumoniae* observed in China was mainly driven by MRMP strains with macrolide resistance‐associated mutations. Phylogenetic analysis revealed two distinct MCCs that recently emerged and co‐circulated across different regions of China during the 2023–2024 epidemic season. We hypothesize that the resurgence and current epidemic of *M. pneumoniae* may be influenced by factors such as genotype shifts or the co‐circulation of both P1 genotypes, potentially exacerbated by a population immunity debt following the COVID‐19 pandemic. Continued surveillance of *M. pneumoniae* infections, along with studies on macrolide resistance and genomic epidemiology, is urgently needed to assess its prevalence and the consequent risk to global public health.

## 4 Materials and Methods

### 4.1 Sample Collection and Detection

We conducted a national surveillance of patients of all ages with acute respiratory tract infection (ARTI) during the *M. pneumoniae* epidemic in China, spanning from November 1, 2023, to December 31, 2024. We selected 43 general hospitals from 40 cities across 24 provinces to actively monitor the prevalence of *M. pneumoniae* (Figure 1A). These sentinel hospitals were part of the national surveillance network for respiratory pathogens, chosen to ensure geographic diversity and include hospitals of various levels for a representative sample. Samples were collected from patients with ARTI based on clinical symptoms, and demographic and clinical data were gathered from all participants. Given the fastidious nature of *M. pneumoniae*, culturing is typically not feasible, making real‐time polymerase chain reaction (RT‐PCR) assays the most reliable diagnostic method. In this study, throat swabs were taken from patients at enrollment, detected by RT‐PCR at local hospitals, and positive samples were sent to our laboratory for further confirmation. Details of the sample detection process are provided in the Supporting Information. Brief oral informed consent was obtained from all adult participants and from a parent or legal guardian of every participant under the age of 18. The provision of this oral consent was contemporaneously documented by the attending physician in the standardized study questionnaire. All procedures performed in studies involving human participants were in accordance with the ethical standards of the institutional research committee and with the 1964 Helsinki declaration and its later amendments or comparable ethical standards.

### 4.2 hc‐tNGS and Analysis

Using the AIdesign platform (iGeneTech, Beijing, China), we designed overlapping 100‐nucleotide (nt) RNA probes that are complementary to and span the entire length of 80 complete genomes of *M. pneumoniae* sourced from the National Center for Biotechnology Information (NCBI) GenBank. This process yielded a total of 27,183 RNA probes. We then selected representative PCR‐positive samples with varying Ct values for hybrid capture‐based enrichment and targeted sequencing. The DNA library from each sample was used for hybrid capture‐based enrichment of *M. pneumoniae* with two rounds of hybridization (IGT Enzyme Plus Library Prep Kit V3, TargetSeq One Hyb & Wash Kit v2.0; iGeneTech, Beijing, China) according to the manufacturer's instructions. Sequencing was conducted on the Illumina NovaSeq 6000 platform (Illumina, San Diego, CA, USA), producing 150 bp paired‐end reads. Quality control of the raw reads was carried out using Trimmomatic version 0.39 [45], and the cleaned reads were mapped to the *M. pneumoniae* reference genome M129 (GenBank accession: GCA_910574535.1) or FH (GenBank accession: GCA_001272835.1) to generate the *M. pneumoniae* consensus sequences using BWA version 0.7.17 [46] and SAMtools version 1.19.2 [47]. *M. pneumoniae* consensus sequences with high sequencing depth (≥ 10x) and coverage (≥ 99%) were utilized for further analysis.

### 4.3 mNGS and Analysis

To evaluate the sequencing performance of hc‐tNGS on *M. pneumoniae*, 28 PCR‐positive samples with varying Ct values (range, 22.08–38.42) were selected for simultaneous sequencing by mNGS. The DNA library for these samples was prepared with the TruSeq DNA PCR‐Free Library Preparation Kit (Illumina) following the manufacturer's guidelines. Sequencing was conducted on the NovaSeq X platform (Illumina), producing 150 bp paired‐end reads. Clean reads were generated by removing sequencing adapters and low‐quality reads with fastp version 0.23.1 [48]. The remaining reads were then aligned to the human reference (hg38) using BWA version 0.7.17, and human reads were subsequently filtered out. *M. pneumoniae* consensus sequences were obtained by processing the reads with Trimmomatic, BWA, and Samtools as previously described. Finally, the results were assessed using qualimap version 2.3 to ensure the quality and reliability of the sequencing data.

### 4.4 SNP Calling and Phylogenetic Analysis

We constructed an ML phylogenetic tree using 271 native genomes sequenced in this study, along with 291 public genomes from the NCBI assembly database. Initially, we performed core genome alignment and variant calling with Snippy v4.6.0 (https://github.com/tseemann/snippy) using *M. pneumoniae* M129 as the reference genome. Considering the influence of horizontal gene transfer on mycoplasma evolution, we identified and masked recombination events through iterative polishing with Gubbins v2.4.1 (https://github.com/nickjcroucher/gubbins), applying default recombination detection thresholds for bacterial pathogen [49]. We then extracted a non‐repetitive core SNP alignment consisting of 3879 invariant sites using SNP‐sites v2.5.1 [49]. Subsequently, we inferred the ML phylogenetic tree from the clean core alignment with FastTree v2.1.11 (http://www.microbesonline.org/fasttree), employing the GTR+CAT model [50, 51]. Population structure was estimated using tree‐independent hierarchical Bayesian clustering through hierarchical Bayesian analysis of population structure (hierBAPS) [52]. The phylogenetic tree was then edited on the iTOL website (https://itol.embl.de/) to enhance visual display and data presentation. Population structure was independently delineated using hierBAPS with hierarchical max.depth set to 2 and n.pops set to 20 to capture fine‐scale clustering patterns. Final tree visualization and annotation were refined using iTOL's advanced features.

### 4.5 Bayesian Phylogeny and Estimation of Demographic History

To address the challenges of reconstructing the evolutionary history of *M. pneumoniae*, a multi‐stage molecular clock calibration strategy was employed. Initially, temporal signals were assessed using TreeTime v0.11.1 (https://github.com/neherlab/treetime), which led to the exclusion of 17 regression‐based outliers and 152 redundant sequences from outbreak clusters. We than created highly curated core SNP alignments with significant temporal signals for lineages P1‐1, P1‐2, L2, and L6. These lineage‐specific SNP sets were analyzed in BEAST v1.10.4 to estimate the most recent common ancestor (MRCA) date and reconstruct historical population dynamics [53, 54]. To determine the most suitable model configuration, we systematically compared various parameter combinations, including substitution models (HKY and GTR), base frequency treatments (empirical and estimated), molecular clock types (strict and relaxed), and coalescent tree priors (constant, exponential, and log‐normal). Model performance was quantitatively assessed using stepping‐stone sampling to estimate marginal likelihoods, incorporating gamma‐distributed rate heterogeneity. Based on these comparisons, and following the canonical framework for demographic inference, the Bayesian skyline model was selected as the coalescent prior. Ultimately, the general time reversible (GTR) substitution model with estimated base frequencies and an uncorrelated relaxed exponential clock provided the best fit to the data [55, 56]. For demographic reconstruction, we applied a Bayesian skyline coalescent prior. The Markov chain Monte Carlo (MCMC) analyses were run for 1 × 10^9^ iterations, with parameters sampled every 1000 steps. A maximum clade credibility (MCC) tree was summarized using TreeAnnotator, excluding the initial 10% of samples as burn‐in. Node support and inferred ancestral locations were represented by posterior probabilities. The MCC tree was visualized in FigTree v1.4.4 (http://tree.bio.ed.ac.uk/software/Figtree). Tracer v1.7.2 (https://github.com/beast‐dev/tracer) was used to examine parameter convergence, summarize the median and 95% highest posterior density (HPD) intervals, and estimate the time to the most recent common ancestor (tMRCA) as well as demographic changes through Bayesian skyline plots. All parameters exhibited effective sample sizes (ESS) greater than 200, indicating sufficient MCMC convergence.

### 4.6 Identification of *p1* Gene Genotype

The two major genotypes of *M. pneumoniae*, known as P1‐1 and P1‐2, are distinguished by variations in the RepMP2/3 and RepMP4 repetitive elements within the P1 protein gene [31, 57, 58]. To identify the P1 genotypes of the strains, we employed MAFFT v7.520 (https://mafft.cbrc.jp/alignment/software/) for multiple sequence alignment and used LAST v1542 (https://gitlab.com/mcfrith/last) to extract the specific region of the *p1* gene. *M. pneumoniae* M129 served as the reference for the P1‐1 genotype, while *M. pneumoniae* FH was used for the P1‐2 genotype [43]. We applied predefined thresholds of ≥ 95% coverage for the RepMP2/3 and RepMP4 segments and > 50% overall coverage of the p1 gene. This method enabled us to accurately determine the P1 genotype of the strains, effectively complementing the traditional phylogenetic tree approach used for P1 protein typing.

### 4.7 MLST

MLST was employed for the characterization of *M. pneumoniae* strain. Targeting eight essential housekeeping genes (*ppa*, *pgm*, *gyrB*, *gmk*, *glyA*, *atpA*, *arcC*, and *adk*), allele calling was performed using MLST v2.23.0 (https://github.com/tseemann/mlst) with the species‐specific *M. pneumoniae* PubMLST scheme [59].

### 4.8 Gene Content Analyses

In this study, the genomes obtained were submitted for the detection of virulence genes and macrolide resistance mutations. The virulence genes of *M. pneumoniae* were identified using ABRicate (https://github.com/tseemann/abricate) with the Virulence Factors Database (VFDB) [60], applying a minimum coverage and identity threshold of 80% for gene detection. Previous studies have demonstrated a significant association between point mutations in the V region of the *M. pneumoniae* 23S rRNA gene and macrolide resistance phenotypes [10, 61, 62]. Consequently, we examined point mutations in the V region of the 23S rRNA using Snippy v4.6.0, which utilized Freebayes v1.3.6 (https://github.com/freebayes/freebayes) for variant calling [63].

### 4.9 Pangenome Analysis

Genome annotation was conducted using Prokka [64], and the annotations from all 562 isolates were used to create a pangenome matrix in Roary [65]. To identify p1‐genotype‐associated accessory genes, we employed a chi‐squared statistical framework, selecting genes with *p*‐values < 1 × 10^−^^1^ ^0^ ^0^. A gene was deemed genotype‐lineage‐enriched if its presence frequency exceeded 75% in one genotype and under 25% in the other, or vice versa. We also assessed the potential impact of lineage structure using the same χ^2^ framework, which revealed only weak signals (10 genes across all lineages, all hypothetical). Functional categorization of these enriched genes was performed by assigning them to Clusters of Orthologous Groups (COG) through BLAST‐based mapping against the COG database. The resulting COG distributions were visualized and summarized in R.

### 4.10 Statistical Analysis

We used logistic regression to examine the relationship between potential risk factors (sex, age, province, and month of infection) and *M. pneumoniae* PCR‐positive. Categorical variables were summarized using proportions with 95% confidence intervals, while continuous variables were described using medians and interquartile ranges. A *p*‐value of less than 0.05 was considered statistically significant. All statistical analyses were conducted using R (v4.3.1), and visualizations were created with ggplot2 (v3.4.4).

## Author Contributions

Guohong Deng, Fusheng Wang, Hongbin Song, and Shaofu Qiu conceived, designed, and supervised the study. Hongbo Liu, Xinying Du, Yele Wang, Yunjie Dan, Huiling Li, Libo Tong, Guohong Deng, and Shaofu Qiu contributed to the acquisition of data. Hongbo Liu, Xinying Du, Yele Wang, Ying Xiang, Qi Wang, Sai Tian, Hongbo Liu, Hui Wang, Chao Wang, Mingjuan Yang, Huiqun Jia, and Xiaoying Li contributed to the experiments. Hongbo Liu, Xiaoyi Zheng, Yule Wang, Yufan Xian, Wenbin Chen, and Shaofu Qiu analyzed the data. Hongbo Liu, Xiaoyi Zheng, Hongbin Song, and Shaofu Qiu interpreted the findings. Hongbo Liu, Xiaoyi Zheng, and Shaofu Qiu wrote the drafts of the manuscript. Hongbo Liu, Xiaoyi Zheng, Huiling Li, and Shaofu Qiu revised the manuscript. All authors read and approved the final report. All authors had full access to all the data in the study and had final responsibility for the decision to submit for publication.

## Funding

This work was supported by the National Key R&D Program of China (Grant Nos. 2022YFC2305303 and 2021YFC2301102).

## Ethics Statement

This study is approved by the Ethics Committee of the First Affiliated Hospital of Army Medical University, PLA ((BA)QX2023028). Informed consent was obtained from all participants in the study.

## Conflicts of Interest

The authors declare no conflicts of interest.

## Supporting information




**Table S1**: *M. Pneumoniae* PCR‐positivity by epidemiological characteristics in mainland China, 2023‐2024.
**Table S2**: Rawbases, mapping rate and genome coverage obtained using mNGS and hc‐tNGS.
**Table S3**: The genome‐sequenced M. pneumoniae strains used in this study.
**Table S4‐A**: Marginal likelihood estimates and pairwise differences between BEAST models.
**Table S4‐B**: Parameter configurations of corresponding BEAST models.
**Table S5**: The accessory genes identified in this study that were associated with *M. pneumoniae* P1 genotypes.
**Figure S1**: Distribution of the 562 global *M. pneumoniae* strains and regional differences in P1 genotypes used in this study.
**Figure S2**: Phylogeny of the two lineages (L2 and L6) of *M. pneumoniae*.
**Figure S3**: Root‐to‐tip regression curves demonstrating temporal signal for P1 genotype and L2/L6 lineages following outlier removal.
**Figure S4**: Bayesian phylogenetic tree and estimation of demographic history of P1 genotype and L2/L6 lineages.
**Figure S5**: The proportion of global *M. pneumoniae* strains carrying the point mutations of 23S rRNA.
**Figure S6**: Pangenome analysis of the global *M. pneumoniae* strains.

## Data Availability

All the sequence data in this study have been deposited in PRJNA1215594, and accession numbers are available in Table S3.
