# Supplementary material for: Genomic‐Based Epidemiological Analysis of the Post‐Pandemic Mycoplasma pneumoniae Resurgence
Source: MedComm (2020). 2026 Jan 22;7(2):e70617. doi: 10.1002/mco2.70617 (PMC12828172; doi:10.1002/mco2.70617)
Supplement: Supplementary file 1 — Table S1: M. Pneumoniae PCR‐positivity by epidemiological characteristics in mainland China, 2023‐2024. Table S2: Rawbases, mapping rate and genome coverage obtained using mNGS and hc‐tNGS. Table S3: The genome‐sequenced M. pneumoniae strains used in this study. Table S4‐A: Marginal likelihood estimates and pairwise differences between BEAST models. Table S4‐B: Parameter configurations of corresponding BEAST models. Table S5: The accessory genes identified in this study that were associated with M. pneumoniae P1 genotypes. Figure S1: Distribution of the 562 global M. pneumoniae strains and regional differences in P1 genotypes used in this study. Figure S2: Phylogeny of the two lineages (L2 and L6) of M. pneumoniae. Figure S3: Root‐to‐tip regression curves demonstrating temporal signal for P1 genotype and L2/L6 lineages following outlier removal. Figure S4: Bayesian phylogenetic tree and estimation of demographic history of P1 genotype and L2/L6 lineages. Figure S5: The proportion of global M. pneumoniae strains carrying the point mutations of 23S rRNA. Figure S6: Pangenome analysis of the global M. pneumoniae strains. [file MCO2-7-e70617-s001.pdf]

Title page

Genomic-Based Epidemiological Analysis of the Post-Pandemic *Mycoplasma Pneumoniae* Resurgence

Hongbo Liu[1, #] (ORCID: 0000-0002-5744-760X), Xiaoyi Zheng[1,2, #], Xinying Du[1, #], Yule Wang[1, #], Ying Xiang[1], Qi Wang[1], Sai Tian[1], Yufan Xian[1], Wenbin Chen[1], Hongbo Liu[1](ORCID: 0000-0002-2320-3682), Hui Wang[1], Chao Wang[1], Mingjuan Yang[1], Huiqun Jia[1], Xiaoying Li[1], Yunjie Dan[3], Libo Tong[4], Guohong Deng[3,5, *], Huiling Li[6,*], Fusheng Wang[7, *], Hongbin Song [1, *], and Shaofu Qiu,[1, *].

[1]Chinese PLA Center for Disease Control and Prevention, Beijing, China.

[2]College of Life Science and Technology, Beijing University of Chemical Technology, Beijing, China.

[3]Department of Infectious Diseases, Southwest Hospital, Third Military Medical University (Army Medical University), Chongqing, China.

[4] The 960th Hospital of the PLA Joint Logistics Support Force, Jinan, China.

[5] Yu-Yue Center for Pathology Research, Chongqing, China.

[6] Hainan Hospital of Chinese People's Liberation Army General Hospital, Sanya, China.

[7] Senior Department of Infectious Diseases, The Fifth Medical Center of Chinese PLA General Hospital, Beijing, China.

#Co-first authors: Hongbo Liu, Xiaoyi Zheng, Xinying Du, Yule Wang.

*Corresponding authors: Guohong Deng, gh_deng@hotmail.com; Huiling Li, 15692538076@126.com; Fusheng Wang, fswang302@163.com; Hongbin Song, hongbinsong@263.net; Shaofu Qiu, qiushf0613@hotmail.com.

SUPPLEMENTARY METHODS

Sample detection

The MagaBio plus Viral DNA/RNA Extraction Kit (Hangzhou Bioer Technology Co. Ltd, Hangzhou, China) was used for nucleic acid extraction according to the manufacturer's instructions. Each nucleic acid extract was tested using either a commercial kit (MABSKY BIO-TECH CO., LTD, Shenzhen, China) or our in-house RT-PCR assay targeting the RepMP1 gene of *M. pneumoniae*. The PCR primers and probe used for our in-house RT-PCR assays are as follows: forward primer qMP-F1 (TCTTTACGCGTTACGTATTC), reverse primer qMP-R1 (AGTGTGGAATTCTCTGGCA) and probe qMP-P1 (FAM-TTCACTGGTATAACCGGTTTGTTAAG-BHQ1). Each reaction of qPCR was conducted in a final volume of 25 μL, comprising 12.5 μL of 2 × Probe qPCR Mix, 0.5 μL of each primer (10 μM concentration), 1.0 μL of probe (10 μM concentration), 5 μL of template DNA, and 5.5 μL of ddH_2O. The amplification protocol consisted of an initial denaturation step at 95°C for 30 seconds, followed by 40 cycles of denaturation at 95°C for 5 seconds, and a combined annealing and extension step at 60°C for 30 seconds. A Ct value of less than 35 indicates a positive result for *M. pneumoniae*. If the Ct value falls within the range of 35 to 40, the sample should undergo retesting. A repeated Ct value within this range confirms a positive result, whereas a value outside this range suggests a negative result.

SUPPLEMENTARY TABLES

Table S1. ***M. Pneumoniae*** **PCR-positivity by epidemiological characteristics in mainland China, 2023-2024.**

	N	MP Positive	OR (95% CI)	p value
All cases with ARTI	98623	9907 (10.05%)		
Sex				
Female	40295	4540 (11.27%)	1 (ref)	
Male	58328	5367 (9.20%)	0.80 (0.76-0.83)	<0.0001
Age group				
Children,⩽5 years	23705	2366 (9.98%)	1 (ref)	
Adolescents,6–17 years	25117	5632 (22.42%)	2.61 (2.48-2.75)	<0.0001
Adults,18–60 years	37249	1718 (4.61%)	0.44 (0.41-0.47)	<0.0001
Older adults, ⩾61 years	12552	191 (1.52%)	0.14 (0.12-0.16)	<0.0001
Province of infection				
Anhui	1719	238 (13.85%)	1 (ref)	
Beijing	9190	1113 (12.11%)	0.86 (0.74-1.00)	0.045
Chongqing	2698	228 (8.45%)	0.57 (0.47-0.70)	<0.0001
Fujian	6641	891(13.42%)	0.96 (0.83-1.13)	0.643
Gansu	1973	130 (6.59%)	0.44 (0.35-0.55)	<0.0001
Guangdong	672	25 (3.72%)	0.24 (0.16-0.37)	<0.0001
Guangxi	4407	163 (3.70%)	0.24 (0.20-0.29)	<0.0001
Hainan	5924	923 (15.58%)	1.15 (0.99-1.34)	0.078
Hebei	5876	1029 (17.51%)	1.32 (1.13-1.54)	<0.0001
Henan	2879	404 (14.03%)	1.02 (0.86-1.21)	0.895
Hubei	3583	153 (4.27%)	0.28 (0.23-0.34)	<0.0001
Hunan	359	56 (15.60%)	1.15 (0.84-1.58)	0.386
Inner Mongolia	2012	389 (19.33%)	1.49 (1.25-1.78)	<0.0001
Liaoning	7262	683 (9.41%)	0.65 (0.55-0.76)	<0.0001
Shaanxi	471	161 (34.18)	3.23 (2.56-4.09)	<0.0001
Shandong	14548	1826 (12.55%)	0.89 (0.77-1.03)	0.128
Sichuan	1407	132 (9.38%)	0.64 (0.51-0.81)	<0.0001
Tianjin	2508	44 (1.75%)	0.11 (0.08-0.15)	<0.0001
Xinjiang	1898	317 (16.70%)	1.25 (1.04-1.50)	0.017
Xizang	1921	65 (3.38%)	0.22 (0.16-0.29)	<0.0001
Yunnan	16123	682 (4.23%)	0.28 (0.24-0.32)	<0.0001
Zhejiang	4552	255 (5.60%)	0.37 (0.31-0.45)	<0.0001
Month of infection				
Nov,2023	11904	1751(14.71%)	1 (ref)	
Dec,2023	18803	1909(10.15%)	0.66 (0.61-0.70)	<0.0001
Jan,2024	17055	1299(7.62%)	0.48 (0.44-0.52)	<0.0001
Feb,2024	7510	463(6.17%)	0.38 (0.34-0.42)	<0.0001
Mar,2024	6771	328(4.84%)	0.30 (0.26-0.33)	<0.0001
Apr,2024	4545	342(7.52%)	0.47 (0.42-0.53)	<0.0001
May,2024	5047	405(8.02%)	0.51 (0.45-0.57)	<0.0001

Jun,2024	4444	427(9.61%)	0.62 (0.55-0.69)	<0.0001
July,2024	4139	469(11.33%)	0.74 (0.67-0.83)	<0.0001
Aug,2024	4200	522(12.43%)	0.82 (0.74-0.91)	<0.0001
Sep,2024	2794	319(11.42%)	0.75 (0.66-0.85)	<0.0001
Oct,2024	2976	543(18.25%)	1.29 (1.16-1.44)	<0.0001
Nov,2024	3631	574(15.81%)	1.09 (0.98-1.21)	0.104
Dec,2024	4804	556(11.57%)	0.76 (0.69-0.84)	<0.0001

Table S2. Rawbases, mapping rate and genome coverage obtained using mNGS and hc-tNGS

		Rawbases (MB)		Mapping rate (%)		10×coverage (%)	
Sample	CT value	mNGS	hc-tNGS	mNGS	hc-tNGS	mNGS	hc-tNGS
90362	27.22	11,341.88	4008.79	0.03	99.47	1.05	100
90363	24.61	14,725.89	3985.85	0.12	99.45	52.38	100
90364	33.44	11,876.82	2996.96	0.01	97.31	0.31	51.94
90365	29.20	10,003.90	3904.99	0.15	99.24	0.99	99.99
90366	28.19	10,812.30	3984.29	0.05	99.16	0.5	99.99
90367	27.71	10,121.87	3649.84	0.02	99.34	0.46	98.41
90368	30.58	10,401.73	3494.55	0.02	99.19	0.83	99.41
90369	31.59	11,151.12	3823.46	0.01	98.77	0.3	99.6
90370	35.40	9,747.10	3647.77	0.01	99.05	0.27	100
90371	30.56	6,504.18	4022.4	0.01	99.31	0.19	97.33
90372	38.42	9,848.07	2844.95	0.04	95.19	0.4	18.45
90373	35.41	11,161.61	3988.27	0.01	99.3	0.29	99.99
90374	27.01	11,186.84	3993	0.02	99.44	0.65	100
90375	34.92	10,622.72	4007.01	0.04	98.86	0.43	99.39
90384	22.08	11,366.49	3194.98	0.22	99.55	99.91	100
90386	34.08	11,474.97	2481.57	0.07	96.12	0.48	45.53
90387	37.71	7,888.90	781.42	0.06	88.37	0.44	20.53
90388	30.80	14,363.26	1114.8	0.06	92.52	0.53	95.88
90394	24.99	6,956.97	3554.15	0.14	99.3	4.97	99.98
90395	23.56	11,747.74	3991.23	0.2	99.52	97.11	100
90396	30.63	11,856.46	3616.7	0.19	99.06	1	100
90397	23.69	12,379.40	3874.09	0.15	99.39	72.1	99.99
90398	32.34	10,643.41	2507.55	0.06	96.56	0.41	99.76
90399	29.12	12,621.11	3894.94	0.2	98.98	0.87	100
90400	26.04	12,215.50	4002.48	0.04	99.49	3.21	99.98
90401	24.75	10,935.04	4016.26	0.11	99.61	51.8	100
90402	24.38	9,642.32	3737.28	0.08	99.41	12.07	100
90403	22.29	11,685.29	3725.57	0.07	99.48	8	100

Table S3. The genome-sequenced *M. pneumoniae* strains used in this study.

Assembly Accession	Assembly Name	Affiliation	Sequencing quality			Year	Continent	Country	Location Detail	Sequence Type	P1 Type*	Lineage		Sample for BEAST	Antibiotic of Antibiotic resistance genes
			Coverage rate (%)	Target mean depth	10X coverage rate (%)							level1	level2		
CP178923	87233	This study	100	94.12	99.96	2023	Asia	China	Beijing	14	P1-2	2	2.2	L2	
CP178924	87309	This study	100	100.93	99.97	2023	Asia	China	Beijing	3	P1-1	6	6.1	p1-1, L6	
CP178925	87310	This study	100	230.27	99.99	2023	Asia	China	Beijing	3	P1-1	6	6.2	p1-1, L6	
CP178926	87311	This study	100	160.54	100	2023	Asia	China	Beijing	3	P1-1	6	6.1	p1-1, L6	
CP178927	87313	This study	100	63.1	99.97	2023	Asia	China	Beijing	3	P1-1	6	6.2	p1-1, L6	
CP178928	87314	This study	100	179.48	100	2023	Asia	China	Beijing	3	P1-1	6	6.2	p1-1, L6	
CP178929	87315	This study	100	380.96	100	2023	Asia	China	Beijing	3	P1-1	6	6.2	p1-1, L6	
CP178930	87320	This study	100	1870.63	100	2023	Asia	China	Beijing	17	P1-1	4	4.1	p1-1	
CP178931	87323	This study	100	101.02	100	2023	Asia	China	Beijing	3	P1-1	6	6.1	p1-1, L6	
CP178932	87324	This study	100	245.47	100	2023	Asia	China	Beijing	3	P1-1	6	6.1	p1-1, L6	
CP178933	87325	This study	100	200.55	99.99	2023	Asia	China	Beijing	3	P1-1	6	6.1	p1-1, L6	
CP178934	87326	This study	100	721.47	100	2023	Asia	China	Beijing	3	P1-1	6	6.1	p1-1, L6	
CP178935	87329	This study	100	1399.65	100	2023	Asia	China	Beijing	3	P1-1	6	6.1	p1-1, L6	
CP178936	87334	This study	99.99	115.51	99.98	2023	Asia	China	Beijing	7 like	P1-2	1	1.4		
CP178937	87335	This study	100	280.53	100	2023	Asia	China	Beijing	3	P1-1	6	6.1	p1-1, L6	
CP178938	87336	This study	100	375.35	99.98	2023	Asia	China	Beijing	14	P1-2	2	2.2	p1-2, L2	
CP178939	87340	This study	100	1190.89	100	2023	Asia	China	Beijing	3	P1-1	6	6.1	p1-1, L6	
CP178940	87341	This study	100	1494.25	100	2023	Asia	China	Beijing	3	P1-1	6	6.1	p1-1, L6	
CP178941	87344	This study	100	292.43	99.98	2023	Asia	China	Beijing	14	P1-2	2	2.2	p1-2, L2	
CP178942	87347	This study	100	125.52	99.98	2023	Asia	China	Beijing	3	P1-1	6	6.2	p1-1, L6	
CP178943	87373	This study	100	1089.28	99.97	2023	Asia	China	Chongqing	7 like	P1-2	1	1.4		
CP178944	87375	This study	100	1141.3	99.99	2023	Asia	China	Chongqing	7	P1-2	1	1.4		
CP178945	87378	This study	100	570.74	100	2023	Asia	China	Chongqing	3	P1-1	6	6.2	p1-1, L6	
CP178946	87382	This study	100	1591.46	100	2023	Asia	China	Chongqing	3	P1-1	6	6.2	p1-1, L6	
CP178947	87428	This study	100	2341.03	100	2023	Asia	China	Fujian	3	P1-1	6	6.1	p1-1, L6	
CP178948	87429	This study	100	1535.49	100	2023	Asia	China	Fujian	3	P1-1	6	6.1	p1-1, L6	
CP178949	87434	This study	100	1779.44	100	2023	Asia	China	Fujian	3	P1-1	6	6.1	p1-1, L6	
CP178950	87439	This study	100	2299.57	99.99	2023	Asia	China	Fujian	14	P1-2	2	2.2	p1-2, L2	
CP178951	87445	This study	100	2298.41	100	2023	Asia	China	Fujian	3	P1-1	6	6.1	p1-1, L6	
CP178952	87446	This study	100	1479.02	99.99	2023	Asia	China	Fujian	3	P1-1	6	6.1	p1-1, L6	
CP178953	87447	This study	100	1673	100	2023	Asia	China	Fujian	3	P1-1	6	6.1	p1-1, L6	
CP178954	87452	This study	100	1955.77	100	2023	Asia	China	Fujian	3	P1-1	6	6.1	p1-1, L6	
CP178955	87454	This study	100	661.21	100	2023	Asia	China	Fujian	3	P1-1	6	6.1	L6	
CP178956	87457	This study	100	2532.38	100	2023	Asia	China	Fujian	3	P1-1	6	6.2	L6	
CP178957	87458	This study	100	1754.34	100	2023	Asia	China	Fujian	3	P1-1	6	6.1	L6	
CP178958	87612	This study	100	1855.32	100	2023	Asia	China	Chongqing	3	P1-1	6	6.1	L6	
CP178959	87615	This study	99.99	40.81	99.3	2023	Asia	China	Chongqing	14	P1-2	2	2.2	L2	
CP178960	87624	This study	100	666.77	100	2023	Asia	China	Chongqing	3	P1-1	6	6.2	L6	
CP178961	87639	This study	100	89.43	99.99	2023	Asia	China	Chongqing	3	P1-1	6	6.1	L6	
CP178962	87657	This study	100	1786.3	100	2023	Asia	China	Fujian	3	P1-1	6	6.1	L6	
CP178963	87659	This study	99.99	47.47	99.29	2023	Asia	China	Fujian	3	P1-1	6	6.2	L6	
CP178964	87660	This study	100	147.69	99.99	2023	Asia	China	Fujian	3	P1-1	6	6.1	L6	
CP178965	87662	This study	100	221.01	99.99	2023	Asia	China	Fujian	3	P1-1	6	6.1	L6	
CP178966	87663	This study	100	59.88	99.81	2023	Asia	China	Fujian	3	P1-1	6	6.1	L6	
CP178967	87664	This study	100	269.8	100	2023	Asia	China	Fujian	3	P1-1	6	6.1	L6	
CP178968	87665	This study	100	75.72	99.88	2023	Asia	China	Fujian	3	P1-1	6	6.1	L6	
CP178969	87666	This study	100	130.3	100	2023	Asia	China	Fujian	3	P1-1	6	6.1	L6	
CP178970	87667	This study	100	155.95	100	2023	Asia	China	Fujian	3	P1-1	6	6.2	L6	
CP178971	87668	This study	100	2298.06	100	2023	Asia	China	Fujian	3	P1-1	6	6.1	L6	
CP178972	87671	This study	99.97	46.82	99.28	2023	Asia	China	Fujian	3	P1-1	6	6.1	L6	
CP178973	87672	This study	100	2322.05	100	2023	Asia	China	Fujian	3	P1-1	6	6.1	L6	
CP178974	87673	This study	99.98	83.49	99.28	2023	Asia	China	Fujian	3	P1-1	6	6.2	L6	
CP178975	87674	This study	100	122.42	99.99	2023	Asia	China	Fujian	3	P1-1	6	6.2	L6	
CP178976	87676	This study	99.99	48.24	99.56	2023	Asia	China	Fujian	3	P1-1	6	6.2	L6	

CP178977	87677	This study	100	1028.34	100	2023	Asia	China	Fujian	3	P1-1	6	6.1	p1-1, L6
CP178978	87679	This study	100	166.16	99.98	2023	Asia	China	Fujian	3	P1-1	6	6.1	p1-1, L6
CP178979	87747	This study	100	220.85	100	2023	Asia	China	Shandong	3	P1-1	6	6.1	p1-1, L6
CP178980	87750	This study	100	113.33	99.97	2023	Asia	China	Shandong	14	P1-2	2	2.2	p1-2, L2
CP178981	87754	This study	100	1872.9	100	2023	Asia	China	Shandong	3	P1-1	6	6.2	p1-1, L6
CP178982	87756	This study	99.99	286.54	99.75	2023	Asia	China	Shandong	3	P1-1	6	6.2	p1-1, L6
CP178983	87758	This study	100	744.17	100	2023	Asia	China	Shandong	3	P1-1	6	6.2	p1-1, L6
CP178984	87761	This study	100	126.23	100	2023	Asia	China	Shandong	3	P1-1	6	6.2	p1-1, L6
CP178985	87765	This study	100	185.91	99.97	2023	Asia	China	Shandong	14	P1-2	2	2.2	p1-2, L2
CP178986	87766	This study	100	844.06	99.98	2023	Asia	China	Shandong	14	P1-2	2	2.2	p1-2, L2
CP178987	87768	This study	100	117.6	100	2023	Asia	China	Shandong	3	P1-1	6	6.1	p1-1, L6
CP178988	87811	This study	100	462.09	100	2023	Asia	China	Chongqing	3	P1-1	6	6.1	p1-1, L6
CP178989	87812	This study	100	1189.09	100	2023	Asia	China	Chongqing	3	P1-1	6	6.1	p1-1, L6
CP178990	87813	This study	100	92.73	99.98	2023	Asia	China	Chongqing	17	P1-1	4	4.2	p1-1
CP178991	87814	This study	100	497.48	100	2023	Asia	China	Chongqing	3 like	P1-1	6	6.1	p1-1, L6
CP178992	87870	This study	100	751.88	99.99	2023	Asia	China	Chongqing	3	P1-1	6	6.1	p1-1, L6
CP178993	87873	This study	100	57.9	99.83	2023	Asia	China	Chongqing	3	P1-1	6	6.1	p1-1, L6
CP178994	87874	This study	100	694.07	100	2023	Asia	China	Chongqing	3	P1-1	6	6.1	p1-1, L6
CP178995	87908	This study	100	582.21	99.98	2023	Asia	China	Anhui	14 like	P1-2	2	2.2	p1-2, L2
CP178996	87916	This study	100	807.77	100	2023	Asia	China	Anhui	3	P1-1	6	6.1	p1-1, L6
CP178997	87929	This study	100	1297.59	99.98	2023	Asia	China	Henan	14	P1-2	2	2.2	p1-2, L2
CP178998	87933	This study	100	1811.14	100	2023	Asia	China	Henan	3	P1-1	6	6.2	p1-1, L6
CP178999	87937	This study	100	889.47	100	2023	Asia	China	Henan	3	P1-1	6	6.2	p1-1, L6
CP179000	87938	This study	100	1556.87	100	2023	Asia	China	Henan	3	P1-1	6	6.1	p1-1, L6
CP179001	87943	This study	100	469.91	100	2023	Asia	China	Henan	3	P1-1	6	6.2	p1-1, L6
CP179002	87951	This study	100	175.03	100	2023	Asia	China	Henan	14 like	P1-2	2	2.2	L2
CP179003	87956	This study	100	1245.36	100	2023	Asia	China	Henan	3	P1-1	6	6.1	p1-1, L6
CP179004	87958	This study	100	477.46	100	2023	Asia	China	Henan	3	P1-1	6	6.2	p1-1, L6
CP179005	87961	This study	100	156.89	100	2023	Asia	China	Henan	3	P1-1	6	6.2	p1-1, L6
CP179006	87971	This study	100	231.94	99.98	2023	Asia	China	Henan	3 like	P1-1	6	6.1	p1-1, L6
CP179007	87975	This study	100	632.53	100	2023	Asia	China	Henan	3	P1-1	6	6.1	p1-1, L6
CP179008	87976	This study	100	202.02	100	2023	Asia	China	Henan	3	P1-1	6	6.1	L6
CP179009	87977	This study	100	294.52	100	2023	Asia	China	Henan	3	P1-1	6	6.1	L6
CP179010	87986	This study	100	1880.01	99.93	2023	Asia	China	Hainan	3	P1-1	6	6.1	L6
CP179011	87988	This study	100	1020.07	100	2023	Asia	China	Hainan	3	P1-1	6	6.1	L6
CP179012	88026	This study	100	1676.66	100	2023	Asia	China	Hainan	3	P1-1	6	6.2	L6
CP179013	88028	This study	100	775.76	99.98	2023	Asia	China	Hainan	14	P1-2	2	2.2	p1-2, L2
CP179014	88029	This study	100	2868.19	100	2023	Asia	China	Hainan	3	P1-1	6	6.2	L6
CP179015	88032	This study	100	894.88	100	2023	Asia	China	Hainan	3	P1-1	6	6.2	L6
CP179016	88034	This study	100	1389.14	100	2023	Asia	China	Hainan	3	P1-1	6	6.2	L6
CP179017	88035	This study	100	660.15	100	2023	Asia	China	Hainan	3	P1-1	6	6.1	L6
CP179018	88053	This study	100	3073.69	100	2023	Asia	China	Hainan	3	P1-1	6	6.2	L6
CP179019	88057	This study	100	859.28	100	2023	Asia	China	Hainan	3	P1-1	6	6.1	L6
CP179020	88059	This study	100	1718.66	100	2023	Asia	China	Hainan	3	P1-1	6	6.1	L6
CP179021	88065	This study	100	2462.97	100	2023	Asia	China	Hainan	3	P1-1	6	6.2	L6
CP179022	88067	This study	100	1978.75	100	2023	Asia	China	Hainan	14	P1-2	2	2.2	p1-2, L2
CP179023	88068	This study	100	763.5	100	2023	Asia	China	Hainan	3	P1-1	6	6.1	L6
CP179024	88071	This study	100	1701.46	100	2023	Asia	China	Hainan	3	P1-1	6	6.1	L6
CP179025	88074	This study	100	957.15	100	2023	Asia	China	Hainan	3	P1-1	6	6.1	L6
CP179026	88076	This study	100	1217.77	100	2023	Asia	China	Hainan	3	P1-1	6	6.1	L6
CP179027	88083	This study	100	1063.72	100	2023	Asia	China	Shandong	3	P1-1	6	6.2	L6
CP179028	88103	This study	100	928.39	100	2023	Asia	China	Shandong	3	P1-1	6	6.2	L6
CP179029	88181	This study	99.96	1045.58	99.93	2023	Asia	China	Hainan	3	P1-1	6	6.1	L6
CP179030	88184	This study	100	1090.8	100	2023	Asia	China	Hainan	3 like	P1-1	6	6.2	L6
CP179031	88186	This study	100	2539.46	100	2023	Asia	China	Hainan	3	P1-1	6	6.1	L6
CP179032	88190	This study	100	961	100	2023	Asia	China	Hainan	3	P1-1	6	6.2	L6
CP179033	88192	This study	100	602.35	100	2023	Asia	China	Hainan	3	P1-1	6	6.2	L6
CP179034	88194	This study	100	2272.2	100	2023	Asia	China	Hainan	3	P1-1	6	6.1	L6
CP179035	88198	This study	100	864.23	100	2023	Asia	China	Hainan	3	P1-1	6	6.2	L6
CP179036	88205	This study	100	1152.32	99.99	2023	Asia	China	Hainan	3	P1-1	6	6.1	L6

CP179037	88223	This study	100	1371.76	100	2023	Asia	China	Hainan	3 like	P1-1	6	6.2	L6
CP179038	88241	This study	100	1213.63	100	2023	Asia	China	Liaoning	3	P1-1	6	6.2	L6
CP179039	88242	This study	100	531.1	100	2023	Asia	China	Liaoning	3	P1-1	6	6.2	L6
CP179040	88245	This study	100	697.62	100	2023	Asia	China	Liaoning	3	P1-1	6	6.2	L6
CP179041	88247	This study	100	741.24	100	2023	Asia	China	Liaoning	3	P1-1	6	6.2	L6
CP179042	88249	This study	100	1994.07	100	2023	Asia	China	Liaoning	3	P1-1	6	6.2	L6
CP179043	88255	This study	100	938.8	100	2023	Asia	China	Liaoning	3	P1-1	6	6.2	L6
CP179044	88257	This study	100	722.32	99.98	2023	Asia	China	Liaoning	14	P1-2	2	2.2	p1-2, L2
CP179045	88368	This study	100	1160.59	99.99	2023	Asia	China	Liaoning	3	P1-1	6	6.2	L6
CP179046	88375	This study	100	1310.39	100	2023	Asia	China	Liaoning	3	P1-1	6	6.2	L6
CP179047	88423	This study	100	197.39	100	2023	Asia	China	Shandong	14	P1-2	2	2.2	p1-2, L2
CP179048	88424	This study	100	779.52	99.98	2023	Asia	China	Shandong	14	P1-2	2	2.2	p1-2, L2
CP179049	88425	This study	99.99	522.65	99.98	2023	Asia	China	Shandong	14 like	P1-2	2	2.2	p1-2, L2
CP179050	88429	This study	100	1834.24	100	2023	Asia	China	Shandong	3	P1-1	6	6.2	L6
CP179051	88509	This study	100	301.59	100	2023	Asia	China	Shandong	3	P1-1	6	6.2	L6
CP179052	88540	This study	100	201.22	100	2023	Asia	China	Shandong	3	P1-1	6	6.2	L6
CP179053	88548	This study	100	297.13	100	2023	Asia	China	Shandong	3	P1-1	6	6.2	L6
CP179054	88551	This study	100	635.65	99.98	2023	Asia	China	Shandong	14	P1-2	2	2.2	p1-2, L2
CP179055	88552	This study	100	2141.25	99.98	2023	Asia	China	Shandong	14	P1-2	2	2.2	p1-2, L2
CP179056	88559	This study	100	262.53	99.99	2023	Asia	China	Shandong	3	P1-1	6	6.2	L6
CP179057	88567	This study	100	413.47	100	2023	Asia	China	Shandong	3	P1-1	6	6.1	L6
CP179058	88582	This study	100	640.39	99.99	2023	Asia	China	Shandong	3	P1-1	6	6.2	L6
CP179059	88585	This study	100	1160.59	99.98	2023	Asia	China	Shandong	14	P1-2	2	2.2	p1-2, L2
CP179060	88587	This study	100	2563.03	100	2023	Asia	China	Shandong	3	P1-1	6	6.1	L6
CP179061	88591	This study	100	1432.63	100	2023	Asia	China	Shandong	3	P1-1	6	6.2	L6
CP179062	88595	This study	100	588.38	100	2023	Asia	China	Shandong	3	P1-1	6	6.2	L6
CP179063	88598	This study	100	2176.63	100	2023	Asia	China	Shandong	3	P1-1	6	6.2	L6
CP179064	88604	This study	99.99	2130.61	99.98	2023	Asia	China	Shandong	14	P1-2	2	2.2	p1-2, L2
CP179065	88612	This study	100	833.91	99.98	2023	Asia	China	Shandong	14	P1-2	2	2.2	p1-2, L2
CP179066	88624	This study	100	1140.7	100	2023	Asia	China	Shandong	3	P1-1	6	6.2	L6
CP179067	88626	This study	100	673.49	100	2023	Asia	China	Shandong	3	P1-1	6	6.2	L6
CP179068	88630	This study	100	1234.63	100	2023	Asia	China	Shandong	3	P1-1	6	6.2	L6
CP179069	88635	This study	100	2009.33	100	2023	Asia	China	Shandong	3	P1-1	6	6.2	L6
CP179070	88636	This study	99.99	468.3	99.98	2023	Asia	China	Shandong	14	P1-2	2	2.2	p1-2, L2
CP179071	88638	This study	100	687.94	100	2023	Asia	China	Shandong	3	P1-1	6	6.2	L6
CP179072	88639	This study	100	2957.1	99.98	2023	Asia	China	Shandong	14	P1-2	2	2.2	p1-2, L2
CP179073	88642	This study	100	1059.38	100	2023	Asia	China	Shandong	3	P1-1	6	6.2	L6
CP179074	88698	This study	100	459.92	100	2023	Asia	China	Shandong	3	P1-1	6	6.2	L6
CP179075	88726	This study	99.99	76.77	99.94	2023	Asia	China	Shandong	14 like	P1-2	2	2.2	p1-2, L2
CP179076	88778	This study	100	2688.93	100	2023	Asia	China	Shandong	3	P1-1	6	6.2	L6
CP179077	88780	This study	100	2385.11	100	2023	Asia	China	Shandong	3	P1-1	6	6.2	L6
CP179078	88790	This study	100	1448.77	100	2023	Asia	China	Shandong	3	P1-1	6	6.2	L6
CP179079	88797	This study	100	1914.07	100	2023	Asia	China	Shandong	3	P1-1	6	6.2	L6
CP179080	88804	This study	100	1766.83	100	2023	Asia	China	Shandong	3	P1-1	6	6.2	L6
CP179081	88841	This study	100	925.89	100	2023	Asia	China	Shandong	3	P1-1	6	6.2	L6
CP179082	88843	This study	100	1957.79	100	2023	Asia	China	Shandong	3	P1-1	6	6.2	L6
CP179083	88900	This study	100	1272.64	100	2023	Asia	China	Shandong	14	P1-2	2	2.2	p1-2, L2
CP179084	88912	This study	100	1179.22	100	2023	Asia	China	Shandong	3	P1-1	6	6.2	L6
CP179085	89077	This study	100	128.31	100	2023	Asia	China	Hebei	3	P1-1	6	6.1	L6
CP179086	89078	This study	100	807.55	100	2023	Asia	China	Hebei	3	P1-1	6	6.2	L6
CP179087	89174	This study	100	1358.32	100	2023	Asia	China	Shandong	3	P1-1	6	6.2	L6
CP179088	89176	This study	100	2078.54	100	2023	Asia	China	Shandong	3	P1-1	6	6.2	L6
CP179089	89178	This study	100	796.16	100	2023	Asia	China	Guangdong	3	P1-1	6	6.2	L6
CP179090	89180	This study	100	1597.46	100	2023	Asia	China	Guangdong	3	P1-1	6	6.2	L6
CP179091	89184	This study	100	779.39	100	2023	Asia	China	Guangdong	3	P1-1	6	6.2	L6
CP179092	89185	This study	100	2310.89	100	2023	Asia	China	Guangdong	3	P1-1	6	6.1	L6
CP179093	89187	This study	100	2599.9	100	2023	Asia	China	Guangdong	3	P1-1	6	6.2	L6
CP179094	89190	This study	100	1313.91	100	2023	Asia	China	Guangdong	3	P1-1	6	6.1	L6
CP179095	89192	This study	100	1421.78	100	2023	Asia	China	Guangdong	3	P1-1	6	6.2	L6
CP179096	89193	This study	100	825.16	100	2023	Asia	China	Guangdong	17	P1-1	4	4.2	

CP179097	89197	This study	100	3057.24	100	2023	Asia	China	Guangdong	3	P1-1	6	6.2	L6
CP179098	89203	This study	100	2498.61	100	2023	Asia	China	Guangdong	3	P1-1	6	6.2	L6
CP179099	89205	This study	100	569.64	99.99	2023	Asia	China	Guangdong	3	P1-1	6	6.2	L6
CP179100	89206	This study	100	485.23	100	2023	Asia	China	Guangdong	3	P1-1	6	6.1	L6
CP179101	89209	This study	100	1959.01	100	2023	Asia	China	Guangdong	3	P1-1	6	6.1	L6
CP179102	89214	This study	100	630.98	100	2023	Asia	China	Guangdong	3	P1-1	6	6.1	L6
CP179103	89216	This study	99.85	911	99.71	2023	Asia	China	Guangdong	3 like	P1-1	6	6.2	L6
CP179104	89225	This study	100	2643.38	99.99	2023	Asia	China	Guangdong	3	P1-1	6	6.1	L6
CP179105	89226	This study	100	2697.68	100	2023	Asia	China	Guangdong	17	P1-1	4	4.4	
CP179106	89229	This study	100	812.82	100	2023	Asia	China	Guangdong	3 like	P1-1	6	6.2	L6
CP179107	89230	This study	100	799.22	100	2023	Asia	China	Guangdong	3	P1-1	6	6.1	L6
CP179108	89233	This study	100	875.96	100	2023	Asia	China	Guangdong	3 like	P1-1	5	5.3	
CP179109	89248	This study	100	2488.18	100	2023	Asia	China	Beijing	3	P1-1	6	6.1	L6
CP179110	89261	This study	99.99	1213.7	99.98	2023	Asia	China	Beijing	14 like	P1-2	2	2.2	p1-2, L2
CP179111	89262	This study	100	2024.25	100	2023	Asia	China	Beijing	3	P1-1	6	6.2	L6
CP179112	89264	This study	100	2663.36	100	2023	Asia	China	Beijing	3	P1-1	6	6.2	L6
CP179113	89277	This study	100	3402.24	99.98	2023	Asia	China	Beijing	14	P1-2	2	2.2	p1-2, L2
CP179114	89286	This study	100	1482.99	100	2023	Asia	China	Beijing	3	P1-1	6	6.1	L6
CP179115	89293	This study	100	2027.41	99.98	2023	Asia	China	Beijing	14 like	P1-2	2	2.2	p1-2, L2
CP179116	89294	This study	100	1070.43	100	2023	Asia	China	Beijing	3	P1-1	6	6.2	L6
CP179117	89307	This study	100	1439.51	100	2023	Asia	China	Beijing	3	P1-1	6	6.2	L6
CP179118	89317	This study	99.99	2016.58	99.99	2023	Asia	China	Beijing	3	P1-1	6	6.1	L6
CP179119	89322	This study	100	1201.84	100	2023	Asia	China	Beijing	3	P1-1	6	6.2	L6
CP179120	89324	This study	100	1768.73	99.99	2023	Asia	China	Beijing	3	P1-1	6	6.1	L6
CP179121	89332	This study	100	1066.32	100	2023	Asia	China	Beijing	3	P1-1	6	6.1	L6
CP179122	89336	This study	100	1558.47	100	2023	Asia	China	Beijing	14	P1-2	2	2.2	p1-2, L2
CP179123	89339	This study	100	1963.49	100	2023	Asia	China	Beijing	3	P1-1	6	6.2	L6
CP179124	89349	This study	100	321.31	100	2023	Asia	China	Zhejiang	3	P1-1	6	6.2	L6
CP179125	89389	This study	99.99	170.92	99.97	2023	Asia	China	Anhui	14	P1-2	2	2.2	p1-2, L2
CP179126	89403	This study	100	109.04	99.97	2023	Asia	China	Anhui	3	P1-1	6	6.1	L6
CP179127	89422	This study	100	418.6	100	2023	Asia	China	Anhui	17	P1-1	4	4.1	
CP179128	89432	This study	100	212.95	100	2023	Asia	China	Shandong	3	P1-1	6	6.2	L6
CP179129	89440	This study	100	1637.03	100	2023	Asia	China	Shandong	3	P1-1	6	6.1	L6
CP179130	89468	This study	100	1201.19	99.99	2023	Asia	China	Shandong	17	P1-1	4	4.1	
CP179131	89541	This study	100	2071.48	100	2023	Asia	China	Shandong	3	P1-1	6	6.2	L6
CP179132	89557	This study	100	972.39	100	2023	Asia	China	Shandong	3	P1-1	6	6.2	L6
CP179133	89599	This study	100	1280.59	100	2023	Asia	China	Shandong	3	P1-1	6	6.1	L6
CP179134	89620	This study	100	3051.64	100	2023	Asia	China	Shandong	3	P1-1	6	6.2	L6
CP179135	89624	This study	100	1832.05	100	2023	Asia	China	Shandong	3	P1-1	6	6.1	L6
CP179136	89627	This study	100	2274.62	100	2023	Asia	China	Shandong	3	P1-1	6	6.1	L6
CP179137	89630	This study	100	2232.5	100	2023	Asia	China	Shandong	3	P1-1	6	6.2	L6
CP179138	89657	This study	100	1381.48	99.98	2023	Asia	China	Shandong	14	P1-2	2	2.2	p1-2, L2
CP179139	89685	This study	100	1470.76	100	2023	Asia	China	Shandong	3	P1-1	6	6.2	L6
CP179140	89690	This study	100	1971.84	100	2023	Asia	China	Shandong	3	P1-1	6	6.1	L6
CP179141	89697	This study	100	1405.17	100	2023	Asia	China	Shandong	3	P1-1	6	6.2	L6
CP179142	89804	This study	100	2153.05	100	2023	Asia	China	Liaoning	3	P1-1	6	6.2	L6
CP179143	89807	This study	100	3242.51	100	2023	Asia	China	Liaoning	3	P1-1	6	6.2	L6
CP179144	89814	This study	100	2887.85	100	2023	Asia	China	Liaoning	3	P1-1	6	6.2	L6
CP179145	89816	This study	100	1254.01	99.99	2023	Asia	China	Liaoning	3	P1-1	6	6.2	L6
CP179146	89823	This study	100	1059.77	100	2023	Asia	China	Liaoning	3	P1-1	6	6.2	L6
CP179147	89825	This study	100	877.34	100	2023	Asia	China	Liaoning	14	P1-2	2	2.2	p1-2, L2
CP179148	89830	This study	100	2103.85	100	2023	Asia	China	Liaoning	3	P1-1	6	6.2	L6
CP179149	89831	This study	100	2253.31	100	2023	Asia	China	Liaoning	3	P1-1	6	6.1	L6
CP179150	89832	This study	100	1527.15	100	2023	Asia	China	Liaoning	3	P1-1	6	6.2	L6
CP179151	89836	This study	100	1736.97	100	2023	Asia	China	Liaoning	3	P1-1	6	6.2	L6
CP179152	89841	This study	100	2826.41	100	2023	Asia	China	Liaoning	3	P1-1	6	6.2	L6
CP179153	89842	This study	100	2523.04	99.99	2023	Asia	China	Liaoning	14 like	P1-2	2	2.2	p1-2, L2
CP179154	89845	This study	100	3324.96	100	2023	Asia	China	Liaoning	3 like	P1-1	6	6.1	L6
CP179155	89848	This study	100	995.97	100	2023	Asia	China	Liaoning	3	P1-1	6	6.2	L6
CP179156	89862	This study	99.99	62.48	99.97	2023	Asia	China	Inner Mongolia	3	P1-1	6	6.1	L6

CP179157	89864	This study	100	1378.68	100	2023	Asia	China	Inner Mongolia	3	P1-1	6	6.2	L6
CP179158	89896	This study	100	720.74	100	2023	Asia	China	Fujian	3	P1-1	6	6.2	L6
CP179159	89905	This study	100	2574.27	100	2023	Asia	China	Fujian	3	P1-1	6	6.1	p1-1, L6
CP179160	89925	This study	100	1334.85	100	2023	Asia	China	Fujian	3 like	P1-1	5	5.1	p1-1
CP179161	89928	This study	100	654.91	99.72	2023	Asia	China	Fujian	3 like	P1-1	6	6.2	L6
CP179162	89934	This study	100	825.11	100	2023	Asia	China	Fujian	3	P1-1	6	6.1	L6
CP179163	89953	This study	100	1295	99.98	2023	Asia	China	Fujian	3	P1-1	6	6.1	p1-1, L6
CP179164	89975	This study	100	939.31	100	2023	Asia	China	Fujian	3	P1-1	6	6.2	p1-1, L6
CP179165	90034	This study	100	1104.39	100	2023	Asia	China	Fujian	3	P1-1	6	6.2	p1-1, L6
CP179166	90041	This study	100	800.51	100	2023	Asia	China	Fujian	3	P1-1	6	6.2	p1-1, L6
CP179167	90045	This study	100	1120.41	99.99	2023	Asia	China	Fujian	3	P1-1	6	6.1	p1-1, L6
CP179168	90145	This study	100	1805.46	100	2023	Asia	China	Shaanxi	3	P1-1	6	6.2	p1-1, L6
CP179169	90362	This study	100	303.94	100	2023	Asia	China	Inner Mongolia	3	P1-1	6	6.1	p1-1, L6
CP179170	90363	This study	100	521.66	100	2023	Asia	China	Inner Mongolia	3	P1-1	6	6.1	p1-1, L6
CP179171	90365	This study	100	132.95	99.99	2023	Asia	China	Inner Mongolia	3	P1-1	6	6.2	p1-1, L6
CP179172	90366	This study	100	188.43	99.99	2023	Asia	China	Inner Mongolia	3	P1-1	6	6.1	p1-1, L6
CP179173	90368	This study	99.99	46.87	99.41	2023	Asia	China	Inner Mongolia	3	P1-1	6	6.1	p1-1, L6
CP179174	90369	This study	100	50.89	99.6	2023	Asia	China	Inner Mongolia	3 like	P1-1	6	6.1	L6
CP179175	90370	This study	100	130.95	100	2023	Asia	China	Inner Mongolia	3	P1-1	6	6.1	p1-1, L6
CP179176	90373	This study	100	120.56	99.99	2023	Asia	China	Inner Mongolia	3	P1-1	6	6.2	p1-1, L6
CP179177	90374	This study	100	295.93	100	2023	Asia	China	Inner Mongolia	3	P1-1	6	6.2	p1-1, L6
CP179178	90375	This study	100	51.17	99.39	2023	Asia	China	Inner Mongolia	3	P1-1	6	6.2	p1-1, L6
CP179179	90384	This study	100	1451.69	100	2023	Asia	China	Inner Mongolia	3	P1-1	6	6.2	p1-1, L6
CP179180	90394	This study	100	1417.26	99.98	2023	Asia	China	Shandong	14	P1-2	2	2.2	p1-2, L2
CP179181	90395	This study	100	2621.88	100	2023	Asia	China	Shandong	3	P1-1	6	6.2	p1-1, L6
CP179182	90396	This study	100	132.67	100	2023	Asia	China	Shandong	3	P1-1	6	6.2	p1-1, L6
CP179183	90397	This study	100	2525.84	99.99	2023	Asia	China	Shandong	3	P1-1	6	6.1	p1-1, L6
CP179184	90398	This study	100	61.07	99.76	2023	Asia	China	Shandong	14 like	P1-2	2	2.2	L2
CP179185	90399	This study	100	229.89	100	2023	Asia	China	Shandong	3	P1-1	6	6.1	p1-1, L6
CP179186	90400	This study	100	836.56	99.98	2023	Asia	China	Shandong	14	P1-2	2	2.2	p1-2, L2
CP179187	90401	This study	100	1584.5	100	2023	Asia	China	Shandong	3	P1-1	6	6.2	p1-1, L6
CP179188	90402	This study	100	2088.24	100	2023	Asia	China	Shandong	3	P1-1	6	6.2	p1-1, L6
CP179189	90403	This study	100	3012.69	100	2023	Asia	China	Shandong	3	P1-1	6	6.2	p1-1, L6
CP179190	A2	This study	100	2998.01	99.99	2023	Asia	China	Hainan	7 like	P1-2	1	1.4	p1-2
CP179191	B2	This study	100	3544.01	99.99	2023	Asia	China	Hainan	7 like	P1-2	1	1.4	p1-2
CP179192	F2	This study	100	3231.99	99.99	2023	Asia	China	Hainan	7 like	P1-2	1	1.4	p1-2
CP179193	G2	This study	100	193.4	99.99	2023	Asia	China	Hainan	7	P1-2	1	1.4	p1-2
GCA_000027345.1	GCA_000027345_1_ASM2734v1	NCBI								1	P1-1	3	3.1	
GCA_000143945.1	GCA_000143945_1_ASM14394v1	NCBI								2	P1-2	1	1.1	
GCA_000283755.1	GCA_000283755_1_ASM28375v1	NCBI								2	P1-2	2	2.1	L2
GCA_000319655.2	GCA_000319655_2_ASM31965v2	NCBI				1980	North America	America	USA: AL, University of Alabama at	2	P1-2	1	1.1	p1-2
GCA_000319675.2	GCA_000319675_2_ASM31967v2	NCBI					North America	America	USA	3	P1-1	5	5.2	
GCA_000331085.2	GCA_000331085_2_ASM33108v2	NCBI					North America	America	USA	1	P1-1	3	3.1	
GCA_000387745.2	GCA_000387745_2_ASM38774v2	NCBI				1994	North America	America	USA: OH	7 like	P1-2	1	1.3	p1-2
GCA_000733995.1	GCA_000733995_1_ASM73399v1	NCBI				2005	Asia	China	China: Beijing	3	P1-1	6	6.1	p1-1, L6
GCA_001272715.1	GCA_001272715_1_ASM127271v1	NCBI				1999	North America	America	USA:AL	2	P1-2	1	1.2	p1-2
GCA_001272735.1	GCA_001272735_1_ASM127273v1	NCBI				2006	North America	America	USA:CO	2 like	P1-1	5	5.2	p1-1
GCA_001272755.1	GCA_001272755_1_ASM127275v1	NCBI				2009	North America	America	USA:AL	2 like	P1-1	5	5.2	p1-1
GCA_001272775.1	GCA_001272775_1_ASM127277v1	NCBI				2009	North America	America	USA:AL	2 like	P1-1	5	5.2	p1-1
GCA_001272795.1	GCA_001272795_1_ASM127279v1	NCBI				1985	Asia	China	China	3 like	P1-1	5	5.2	p1-1
GCA_001272815.1	GCA_001272815_1_ASM127281v1	NCBI				1985	Asia	China	China	3 like	P1-1	5	5.2	p1-1
GCA_001272835.1	GCA_001272835_1_ASM127283v1	NCBI				1954	North America	America	USA:MA	2	P1-2	1	1.1	p1-2
GCA_001272855.1	GCA_001272855_1_ASM127285v1	NCBI				1981	Europe	England	UK	4	P1-2	1	1.3	p1-2
GCA_001272875.1	GCA_001272875_1_ASM127287v1	NCBI				1982	Europe	England	UK	7 like	P1-2	1	1.3	p1-2
GCA_001272895.1	GCA_001272895_1_ASM127289v1	NCBI				1982	Europe	England	UK	2	P1-2	1	1.2	p1-2
GCA_001272915.1	GCA_001272915_1_ASM127291v1	NCBI				1944	North America	America	USA:CA	2	P1-2	1	1.3	p1-2
GCA_001296485.1	GCA_001296485_1_ASM129648v1	NCBI				1999	Europe	France	France	2 like	not available	5	5.2	p1-1
GCA_001296505.1	GCA_001296505_1_ASM129650v1	NCBI				1996	Europe	France	France	3	not available	5	5.2	p1-1

GCA_001296515.1	GCA_001296515_1_ASM129651v1	NCBI	2001	Europe	France	France	7 like	not available	1	1.3	p1-2
GCA_001296525.1	GCA_001296525_1_ASM129652v1	NCBI	1999	Europe	Spain	Spain	2	not available	1	1.1	p1-2
GCA_001296565.1	GCA_001296565_1_ASM129656v1	NCBI	2005	Europe	France	France	14	not available	2	2.1	p1-2, L2
GCA_001296585.1	GCA_001296585_1_ASM129658v1	NCBI	2011	Europe	France	France	3	P1-1	5	5.2	p1-1
GCA_001296605.1	GCA_001296605_1_ASM129660v1	NCBI		Asia	Japan		7 like	not available	1	1.3	
GCA_001296615.1	GCA_001296615_1_ASM129661v1	NCBI	2006	Africa	Tunisia	Tunisia	1	not available	3	3.1	p1-1
GCA_001296625.1	GCA_001296625_1_ASM129662v1	NCBI	2008	Europe	France	France	2	not available	1	1.2	p1-2
GCA_001296665.1	GCA_001296665_1_ASM129666v1	NCBI	1993	Europe	Germany	Germany	3	not available	5	5.2	p1-1
GCA_001296685.1	GCA_001296685_1_ASM129668v1	NCBI	1991	Europe	Germany	Germany	2	P1-2	2	2.1	p1-2, L2
GCA_001296705.1	GCA_001296705_1_ASM129670v1	NCBI	2011	Europe	France	France	2 like	not available	5	5.2	p1-1
GCA_001296725.1	GCA_001296725_1_ASM129672v1	NCBI	2011	Europe	France	France	3	not available	5	5.2	p1-1
GCA_001296735.1	GCA_001296735_1_ASM129673v1	NCBI		Europe	France	France	3	not available	5	5.2	
GCA_001296765.1	GCA_001296765_1_ASM129676v1	NCBI	2011	Europe	France	France	14 like	not available	2	2.1	p1-2, L2
GCA_001296785.1	GCA_001296785_1_ASM129678v1	NCBI	2011	Europe	France	France	2	not available	1	1.2	p1-2
GCA_001296805.1	GCA_001296805_1_ASM129680v1	NCBI	1967	Europe	Denmark	Denmark	2	not available	1	1.3	p1-2
GCA_001296815.1	GCA_001296815_1_ASM129681v1	NCBI	2005	Europe	France	France	3	not available	5	5.3	p1-1
GCA_001296825.1	GCA_001296825_1_ASM129682v1	NCBI	2003	Asia	Japan	Japan	7 like	not available	1	1.3	p1-2
GCA_001296855.1	GCA_001296855_1_ASM129685v1	NCBI	1988	Asia	Japan	Japan	1	not available	3	3.1	p1-1
GCA_001296885.1	GCA_001296885_1_ASM129688v1	NCBI	2011	Europe	France	France	3	not available	5	5.2	p1-1
GCA_001296895.1	GCA_001296895_1_ASM129689v1	NCBI	2011	Europe	France	France	3	not available	5	5.2	p1-1
GCA_001296905.1	GCA_001296905_1_ASM129690v1	NCBI	1979	Europe	France	France	3	not available	5	5.2	p1-1
GCA_001455605.1	GCA_001455605_1_ASM145560v1	NCBI	2010				3	not available	6	6.1	p1-1, L6
GCA_001455625.1	GCA_001455625_1_ASM145562v1	NCBI	2012				3	not available	6	6.2	p1-1, L6
GCA_001455635.1	GCA_001455635_1_ASM145563v1	NCBI	2018	Asia	China	China:Taiwan	3	not available	6	6.1	p1-1, L6
GCA_001455675.1	GCA_001455675_1_ASM145567v1	NCBI	1980	Asia	Japan	Japan:Kanagawa	3	not available	6	6.1	p1-1, L6
GCA_001455685.1	GCA_001455685_1_ASM145568v1	NCBI	2012				3	not available	6	6.2	p1-1, L6
GCA_001455695.1	GCA_001455695_1_ASM145569v1	NCBI	2012				3	not available	6	6.1	p1-1, L6
GCA_001455735.1	GCA_001455735_1_ASM145573v1	NCBI	2012				3	not available	6	6.1	p1-1, L6
GCA_001455745.1	GCA_001455745_1_ASM145574v1	NCBI	2012				3	not available	6	6.1	p1-1, L6
GCA_001455775.1	GCA_001455775_1_ASM145577v1	NCBI	2012				3	not available	6	6.1	p1-1, L6
GCA_001455795.1	GCA_001455795_1_ASM145579v1	NCBI	2012				3 like	not available	5	5.1	p1-1
GCA_001509195.1	GCA_001509195_1_ASM150919v1	NCBI	2012	Asia	China	China	3	P1-1	6	6.1	p1-1, L6
GCA_001558175.1	GCA_001558175_1_ASM155817v1	NCBI	2012	Asia	China	China: Beijing	3	P1-1	6	6.1	p1-1, L6
GCA_001901705.1	GCA_001901705_1_ASM190170v1	NCBI	1954	North America	America	USA: Massachusetts	2	P1-2	1	1.1	p1-2
GCA_002090215.1	GCA_002090215_1_ASM209021v1	NCBI	2016	Asia	China	China: Beijing	1	P1-1	3	3.2	p1-1
GCA_002090235.1	GCA_002090235_1_ASM209023v1	NCBI	2016	Asia	China	China: Beijing	1	P1-1	3	3.2	p1-1
GCA_002090275.1	GCA_002090275_1_ASM209027v1	NCBI	2015	Asia	China	China: Beijing	1	P1-1	3	3.2	p1-1
GCA_002090295.1	GCA_002090295_1_ASM209029v1	NCBI	2015	Asia	China	China: Beijing	1	P1-1	3	3.2	p1-1
GCA_002090315.1	GCA_002090315_1_ASM209031v1	NCBI	2016	Asia	China	China: Beijing	1	P1-1	3	3.2	p1-1
GCA_002095995.1	GCA_002095995_1_ASM209599v1	NCBI	2016	Asia	China	China: Beijing	1	P1-1	3	3.2	p1-1
GCA_002096015.1	GCA_002096015_1_ASM209601v1	NCBI	2016	Asia	China	China: Beijing	1	P1-1	3	3.2	p1-1
GCA_002096035.1	GCA_002096035_1_ASM209603v1	NCBI	2016	Asia	China	China: Beijing	1	P1-1	3	3.2	p1-1
GCA_002127985.1	GCA_002127985_1_ASM212798v1	NCBI	1988	Europe	Denmark	Denmark	19	P1-1	5	5.2	p1-1
GCA_002128005.1	GCA_002128005_1_ASM212800v1	NCBI	2009	Africa	Egypt	Egypt	14 like	P1-2	2	2.1	p1-2, L2
GCA_002128025.1	GCA_002128025_1_ASM212802v1	NCBI	1965	North America	America	USA: Washington	1	P1-1	3	3.2	p1-1
GCA_002128045.1	GCA_002128045_1_ASM212804v1	NCBI	2012	North America	America	USA: Florida	3	P1-1	5	5.3	p1-1
GCA_002128065.1	GCA_002128065_1_ASM212806v1	NCBI	2010	Africa	Egypt	Egypt	2 like	P1-1	5	5.2	p1-1
GCA_002128085.1	GCA_002128085_1_ASM212808v1	NCBI	2007	North America	America	USA: Rhode Island	2	P1-2	1	1.2	p1-2
GCA_002128105.1	GCA_002128105_1_ASM212810v1	NCBI	2000	North America	America	JSA: Washington DC	2	P1-2	1	1.2	p1-2
GCA_002128125.1	GCA_002128125_1_ASM212812v1	NCBI	2014	North America	America	USA: Colorado	14	P1-2	2	2.1	p1-2, L2
GCA_002128145.1	GCA_002128145_1_ASM212814v1	NCBI	2012	North America	America	USA: Florida	2 like	P1-1	5	5.2	p1-1
GCA_002128165.1	GCA_002128165_1_ASM212816v1	NCBI	2013	North America	America	USA: Colorado	2	P1-2	1	1.2	p1-2
GCA_002128185.1	GCA_002128185_1_ASM212818v1	NCBI	2010	Africa	Kenya	Kenya	16	P1-2	1	1.3	p1-2
GCA_002128205.1	GCA_002128205_1_ASM212820v1	NCBI	1999	North America	America	USA: New York	7 like	P1-2	1	1.3	p1-2
GCA_002128235.1	GCA_002128235_1_ASM212823v1	NCBI	1999	North America	America	USA: Indiana	2	P1-2	1	1.2	p1-2
GCA_002128265.1	GCA_002128265_1_ASM212826v1	NCBI	1995	North America	America	USA: California	15	P1-2	2	2.1	p1-2, L2
GCA_002128285.1	GCA_002128285_1_ASM212828v1	NCBI	2012	North America	America	USA: Georgia	2	P1-2	1	1.2	p1-2
GCA_002147855.1	GCA_002147855_1_ASM214785v1	NCBI	1968	North America	America	USA: North Carolina	1	P1-1	3	3.1	p1-1
GCA_002355695.1	GCA_002355695_1_ASM235569v1	NCBI	2012	Asia	Japan	Japan	2 like	P1-2	1	1.2	p1-2
GCA_002355715.1	GCA_002355715_1_ASM235571v1	NCBI	2012	Asia	Japan	Japan	14	P1-2	2	2.1	p1-2, L2

GCA_002563345.1	GCA_002563345_1_ASM256334v1	NCBI	2013	North America	America	USA: Colorado	3	P1-1	5	5.3	p1-1
GCA_002563355.1	GCA_002563355_1_ASM256335v1	NCBI		Europe	Denmark	Denmark	2	P1-2	1	1.2	
GCA_002563365.1	GCA_002563365_1_ASM256336v1	NCBI	1999	North America	America	USA: New York	7 like	P1-2	1	1.3	p1-2
GCA_002563415.1	GCA_002563415_1_ASM256341v1	NCBI	1998	Africa	Kenya	Kenya	3	P1-1	5	5.2	p1-1
GCA_002563435.1	GCA_002563435_1_ASM256343v1	NCBI	2010	Africa	Kenya	Kenya	3	P1-1	5	5.2	p1-1
GCA_002563495.1	GCA_002563495_1_ASM256349v1	NCBI	1991	North America	America	USA: Alabama	1	P1-1	3	3.1	p1-1
GCA_002563515.1	GCA_002563515_1_ASM256351v1	NCBI	2010	North America	Guatemala	Guatemala	3	P1-1	5	5.2	p1-1
GCA_002563545.1	GCA_002563545_1_ASM256354v1	NCBI	1954	North America	America	USA: Massachusetts	2	P1-2	1	1.1	p1-2
GCA_009809995.1	GCA_009809995_1_ASM980999v1	NCBI	1976	Asia	Japan	Japan	7	not available	1	1.3	p1-2
GCA_009810015.1	GCA_009810015_1_ASM981001v1	NCBI	1984	Asia	Japan	Japan	7	not available	1	1.3	p1-2
GCA_009810035.1	GCA_009810035_1_ASM981003v1	NCBI	1983	Asia	Japan	Japan	7	not available	1	1.3	p1-2
GCA_009810055.1	GCA_009810055_1_ASM981005v1	NCBI	2012	Asia	Japan	Japan	7	not available	1	1.3	p1-2
GCA_009810075.1	GCA_009810075_1_ASM981007v1	NCBI	1988	Asia	Japan	Japan	30 like	not available	5	5.1	p1-1
GCA_009810095.1	GCA_009810095_1_ASM981009v1	NCBI	1989	Asia	Japan	Japan	3	not available	5	5.2	p1-1
GCA_009810115.1	GCA_009810115_1_ASM981011v1	NCBI	1992	Asia	Japan	Japan	3	P1-1	5	5.2	p1-1
GCA_009810135.1	GCA_009810135_1_ASM981013v1	NCBI	2010	Asia	Japan	Japan	7	not available	1	1.3	p1-2
GCA_009810155.1	GCA_009810155_1_ASM981015v1	NCBI	2011	Asia	Japan	Japan	14	not available	2	2.1	p1-2, L2
GCA_009810175.1	GCA_009810175_1_ASM981017v1	NCBI	2013	Asia	Japan	Japan	14	not available	2	2.1	p1-2, L2
GCA_009810195.1	GCA_009810195_1_ASM981019v1	NCBI	2013	Asia	Japan	Japan	16	not available	1	1.3	p1-2
GCA_009810235.1	GCA_009810235_1_ASM981023v1	NCBI	2011	Asia	Japan	Japan	3	P1-1	5	5.2	p1-1
GCA_009810255.1	GCA_009810255_1_ASM981025v1	NCBI	2011	Asia	Japan	Japan	14	not available	2	2.1	p1-2, L2
GCA_009810275.1	GCA_009810275_1_ASM981027v1	NCBI	2011	Asia	Japan	Japan	14	not available	2	2.1	p1-2, L2
GCA_009810295.1	GCA_009810295_1_ASM981029v1	NCBI	2008	Asia	Japan	Japan	3	P1-1	5	5.1	p1-1
GCA_009810315.1	GCA_009810315_1_ASM981031v1	NCBI	1979	Asia	Japan	Japan	7	not available	1	1.3	p1-2
GCA_009810335.1	GCA_009810335_1_ASM981033v1	NCBI	1980	Asia	Japan	Japan	7	not available	1	1.3	p1-2
GCA_009810355.1	GCA_009810355_1_ASM981035v1	NCBI	1983	Asia	Japan	Japan	3	P1-1	5	5.1	p1-1
GCA_009810375.1	GCA_009810375_1_ASM981037v1	NCBI	2005	Europe	France	France	7 like	not available	1	1.3	p1-2
GCA_009810395.1	GCA_009810395_1_ASM981039v1	NCBI	1985	Asia	Japan	Japan	3	P1-1	5	5.2	p1-1
GCA_009810415.1	GCA_009810415_1_ASM981041v1	NCBI	1987	Asia	Japan	Japan	7	P1-2	1	1.3	p1-2
GCA_009810435.1	GCA_009810435_1_ASM981043v1	NCBI	1988	Asia	Japan	Japan	3	P1-1	5	5.2	p1-1
GCA_009810455.1	GCA_009810455_1_ASM981045v1	NCBI	2019	Asia	China	China:Taiwan	3	P1-1	5	5.2	
GCA_009810475.1	GCA_009810475_1_ASM981047v1	NCBI	1990	Asia	Japan	Japan	3	not available	5	5.2	p1-1
GCA_009810495.1	GCA_009810495_1_ASM981049v1	NCBI	1991	Asia	Japan	Japan	2	not available	2	2.1	p1-2, L2
GCA_009810515.1	GCA_009810515_1_ASM981051v1	NCBI	1991	Asia	Japan	Japan	2	P1-2	2	2.1	p1-2, L2
GCA_009810535.1	GCA_009810535_1_ASM981053v1	NCBI	1993	Asia	Japan	Japan	2	not available	2	2.1	p1-2, L2
GCA_009810555.1	GCA_009810555_1_ASM981055v1	NCBI	1994	Asia	Japan	Japan	7	P1-2	1	1.3	p1-2
GCA_009810575.1	GCA_009810575_1_ASM981057v1	NCBI	2013	Asia	Japan	Japan	3	P1-1	6	6.1	p1-1, L6
GCA_009810595.1	GCA_009810595_1_ASM981059v1	NCBI	2008	Asia	Japan	Japan	3	P1-1	5	5.1	p1-1
GCA_009810615.1	GCA_009810615_1_ASM981061v1	NCBI	2009	Asia	Japan	Japan	14	P1-2	2	2.1	p1-2, L2
GCA_009810635.1	GCA_009810635_1_ASM981063v1	NCBI	2010	Asia	Japan	Japan	14	not available	2	2.1	p1-2, L2
GCA_009810655.1	GCA_009810655_1_ASM981065v1	NCBI	2011	Asia	Japan	Japan	14	P1-2	2	2.1	p1-2, L2
GCA_009810675.1	GCA_009810675_1_ASM981067v1	NCBI	2011	Asia	Japan	Japan	14	not available	2	2.1	p1-2, L2
GCA_009810695.1	GCA_009810695_1_ASM981069v1	NCBI	2012	Asia	Japan	Japan	3	P1-1	5	5.2	p1-1
GCA_009810715.1	GCA_009810715_1_ASM981071v1	NCBI	2012	Asia	Japan	Japan	14 like	P1-2	2	2.1	p1-2, L2
GCA_009810735.1	GCA_009810735_1_ASM981073v1	NCBI	2012	Asia	Japan	Japan	3	P1-1	6	6.1	p1-1, L6
GCA_009810755.1	GCA_009810755_1_ASM981075v1	NCBI	2012	Asia	Japan	Japan	3	P1-1	5	5.2	p1-1
GCA_009810775.1	GCA_009810775_1_ASM981077v1	NCBI	1985	Asia	Japan	Japan	3	P1-1	5	5.1	p1-1
GCA_009810795.1	GCA_009810795_1_ASM981079v1	NCBI	2016	Asia	Japan	Japan	3	not available	6	6.1	p1-1, L6
GCA_009810815.1	GCA_009810815_1_ASM981081v1	NCBI	2016	Asia	Japan	Japan	3	not available	6	6.1	p1-1, L6
GCA_009810835.1	GCA_009810835_1_ASM981083v1	NCBI	2016	Asia	Japan	Japan	3	P1-1	6	6.1	p1-1, L6
GCA_009810855.1	GCA_009810855_1_ASM981085v1	NCBI	2011	Asia	Japan	Japan	14	not available	2	2.1	p1-2, L2
GCA_009810875.1	GCA_009810875_1_ASM981087v1	NCBI	2011	Asia	Japan	Japan	14	not available	2	2.1	p1-2, L2
GCA_009810895.1	GCA_009810895_1_ASM981089v1	NCBI	2011	Asia	Japan	Japan	7	not available	1	1.4	p1-2
GCA_009810915.1	GCA_009810915_1_ASM981091v1	NCBI	2011	Asia	Japan	Japan	3	not available	5	5.1	p1-1
GCA_009810935.1	GCA_009810935_1_ASM981093v1	NCBI	2011	Asia	Japan	Japan	14	not available	2	2.1	p1-2, L2
GCA_009810955.1	GCA_009810955_1_ASM981095v1	NCBI	2011	Asia	Japan	Japan	3	not available	6	6.1	p1-1, L6
GCA_009810975.1	GCA_009810975_1_ASM981097v1	NCBI	2011	Asia	Japan	Japan	3	not available	5	5.3	p1-1
GCA_009810995.1	GCA_009810995_1_ASM981099v1	NCBI	2011	Asia	Japan	Japan	14	not available	2	2.2	p1-2, L2
GCA_009811015.1	GCA_009811015_1_ASM981101v1	NCBI	2011	Asia	Japan	Japan	3 like	not available	6	6.1	p1-1, L6
GCA_009811035.1	GCA_009811035_1_ASM981103v1	NCBI	2011	Asia	Japan	Japan	14 like	not available	2	2.1	p1-2, L2

GCA_009811055.1	GCA_009811055_1_ASM981105v1	NCBI	2011	Asia	Japan	Japan	3	not available	6	6.1	p1-1, L6
GCA_009811075.1	GCA_009811075_1_ASM981107v1	NCBI	2011	Asia	Japan	Japan	14	not available	2	2.1	p1-2, L2
GCA_009811095.1	GCA_009811095_1_ASM981109v1	NCBI	2011	Asia	Japan	Japan	3	not available	6	6.1	p1-1, L6
GCA_009811115.1	GCA_009811115_1_ASM981111v1	NCBI	2011	Asia	Japan	Japan	14	not available	2	2.1	p1-2, L2
GCA_009811135.1	GCA_009811135_1_ASM981113v1	NCBI	2011	Asia	Japan	Japan	3 like	not available	5	5.1	p1-1
GCA_009811155.1	GCA_009811155_1_ASM981115v1	NCBI	2011	Asia	Japan	Japan	7	not available	1	1.4	p1-2
GCA_009811175.1	GCA_009811175_1_ASM981117v1	NCBI	2011	Asia	Japan	Japan	14	not available	2	2.1	p1-2, L2
GCA_009811195.1	GCA_009811195_1_ASM981119v1	NCBI	2011	Asia	Japan	Japan	14	not available	2	2.2	p1-2, L2
GCA_009811215.1	GCA_009811215_1_ASM981121v1	NCBI	2011	Asia	Japan	Japan	7	not available	1	1.4	p1-2
GCA_009811235.1	GCA_009811235_1_ASM981123v1	NCBI	2011	Asia	Japan	Japan	7	not available	1	1.4	p1-2
GCA_009811255.1	GCA_009811255_1_ASM981125v1	NCBI	2011	Asia	Japan	Japan	3	not available	5	5.3	p1-1
GCA_009811275.1	GCA_009811275_1_ASM981127v1	NCBI	2011	Asia	Japan	Japan	3	not available	6	6.2	p1-1, L6
GCA_009811295.1	GCA_009811295_1_ASM981129v1	NCBI	2011	Asia	Japan	Japan	7	not available	1	1.4	p1-2
GCA_009939745.1	GCA_009939745_1_ASM993974v1	NCBI	2016	Asia	South Korea	South Korea: Seoul	14	P1-2	2	2.2	p1-2, L2
GCA_009939765.1	GCA_009939765_1_ASM993976v1	NCBI	2016	Asia	South Korea	South Korea: Seoul	3	P1-1	6	6.1	p1-1, L6
GCA_009939785.1	GCA_009939785_1_ASM993978v1	NCBI	2016	Asia	South Korea	South Korea: Seoul	3	P1-1	6	6.1	p1-1, L6
GCA_009939805.1	GCA_009939805_1_ASM993980v1	NCBI	2016	Asia	South Korea	South Korea: Seoul	3	P1-1	6	6.1	p1-1, L6
GCA_009939825.1	GCA_009939825_1_ASM993982v1	NCBI	2016	Asia	South Korea	South Korea: Seoul	3	P1-1	6	6.1	p1-1, L6
GCA_009939845.1	GCA_009939845_1_ASM993984v1	NCBI	2016	Asia	South Korea	South Korea: Seoul	3	P1-1	6	6.1	p1-1, L6
GCA_009939975.1	GCA_009939975_1_ASM993997v1	NCBI	2016	Asia	South Korea	South Korea: Seoul	3	P1-1	6	6.1	p1-1, L6
GCA_009940325.1	GCA_009940325_1_ASM994032v1	NCBI	2015	Asia	South Korea	South Korea: Seoul	3	P1-1	6	6.2	p1-1, L6
GCA_009940965.1	GCA_009940965_1_ASM994096v1	NCBI	2015	Asia	South Korea	South Korea: Seoul	3	P1-1	6	6.1	p1-1, L6
GCA_009941325.1	GCA_009941325_1_ASM994132v1	NCBI	2015	Asia	South Korea	South Korea: Seoul	3	P1-1	6	6.2	p1-1, L6
GCA_009941705.1	GCA_009941705_1_ASM994170v1	NCBI	2015	Asia	South Korea	South Korea: Seoul	3	P1-1	6	6.2	p1-1, L6
GCA_009942155.1	GCA_009942155_1_ASM994215v1	NCBI	2014	Asia	South Korea	South Korea: Seoul	14	P1-2	2	2.1	p1-2, L2
GCA_009942395.1	GCA_009942395_1_ASM994239v1	NCBI	2012	Asia	South Korea	South Korea: Seoul	3	P1-1	6	6.1	p1-1, L6
GCA_009942655.1	GCA_009942655_1_ASM994265v1	NCBI	2012	Asia	South Korea	South Korea: Seoul	3	P1-1	6	6.1	p1-1, L6
GCA_009942915.1	GCA_009942915_1_ASM994291v1	NCBI	2011	Asia	South Korea	South Korea: Seoul	33	P1-2	2	2.1	p1-2, L2
GCA_009943205.1	GCA_009943205_1_ASM994320v1	NCBI	2011	Asia	South Korea	South Korea: Seoul	3	P1-1	5	5.3	p1-1
GCA_009943505.1	GCA_009943505_1_ASM994350v1	NCBI	2011	Asia	South Korea	South Korea: Seoul	14	P1-2	2	2.1	p1-2, L2
GCA_009943805.1	GCA_009943805_1_ASM994380v1	NCBI	2011	Asia	South Korea	South Korea: Seoul	3	P1-1	6	6.2	p1-1, L6
GCA_009944075.1	GCA_009944075_1_ASM994407v1	NCBI	2011	Asia	South Korea	South Korea: Seoul	3	P1-1	5	5.2	p1-1
GCA_009944335.1	GCA_009944335_1_ASM994433v1	NCBI	2011	Asia	South Korea	South Korea: Seoul	3	P1-1	6	6.1	p1-1, L6
GCA_009944725.1	GCA_009944725_1_ASM994472v1	NCBI	2011	Asia	South Korea	South Korea: Seoul	14	P1-2	2	2.1	p1-2, L2
GCA_009945165.1	GCA_009945165_1_ASM994516v1	NCBI	2011	Asia	South Korea	South Korea: Seoul	17	P1-1	4	4.3	p1-1
GCA_009945535.1	GCA_009945535_1_ASM994553v1	NCBI	2011	Asia	South Korea	South Korea: Seoul	3	P1-1	5	5.3	p1-1
GCA_009945865.1	GCA_009945865_1_ASM994586v1	NCBI	2010	Asia	South Korea	South Korea: Seoul	14	P1-2	2	2.1	p1-2, L2
GCA_009946285.1	GCA_009946285_1_ASM994628v1	NCBI	2010	Asia	South Korea	South Korea: Seoul	3	P1-1	5	5.2	p1-1
GCA_009946845.1	GCA_009946845_1_ASM994684v1	NCBI	2010	Asia	South Korea	South Korea: Seoul	3	P1-1	6	6.2	p1-1, L6
GCA_009947205.1	GCA_009947205_1_ASM994720v1	NCBI	2010	Asia	South Korea	South Korea: Seoul	1	P1-1	3	3.1	p1-1
GCA_009947575.1	GCA_009947575_1_ASM994757v1	NCBI	2010	Asia	South Korea	South Korea: Seoul	17	P1-1	4	4.3	p1-1
GCA_009947985.1	GCA_009947985_1_ASM994798v1	NCBI	2010	Asia	South Korea	South Korea: Seoul	3	P1-1	6	6.2	p1-1, L6
GCA_009948395.1	GCA_009948395_1_ASM994839v1	NCBI	2010	Asia	South Korea	South Korea: Seoul	1	P1-1	3	3.1	p1-1
GCA_030159355.1	GCA_030159355_1_ASM3015935v1	NCBI	2019	Asia	China	China:Taiwan	8	not available	1	1.2	p1-2
GCA_030159375.1	GCA_030159375_1_ASM3015937v1	NCBI	2020	Asia	Japan	Japan:Saitama	17 like	P1-1	4	4.2	p1-1
GCA_030159395.1	GCA_030159395_1_ASM3015939v1	NCBI	2020	Asia	Japan	Japan:Saitama	2	not available	1	1.2	p1-2
GCA_030159415.1	GCA_030159415_1_ASM3015941v1	NCBI	2020	Asia	Japan	Japan:Saitama	7	not available	1	1.4	p1-2
GCA_030159435.1	GCA_030159435_1_ASM3015943v1	NCBI	2020	Asia	Japan	Japan:Osaka	17	P1-1	4	4.2	p1-1
GCA_030159455.1	GCA_030159455_1_ASM3015945v1	NCBI	2020	Asia	Japan	Japan:Osaka	3	P1-1	6	6.1	p1-1, L6
GCA_030923165.1	GCA_030923165_1_ASM3092316v1	NCBI	2019	Asia	China	China:Taiwan	3	P1-1	6	6.1	p1-1, L6
GCA_030923175.1	GCA_030923175_1_ASM3092317v1	NCBI	2019	Asia	China	China:Taiwan	14	not available	2	2.2	p1-2, L2
GCA_030923185.1	GCA_030923185_1_ASM3092318v1	NCBI	1980	Asia	China	China:Taiwan	17	not available	4	4.4	p1-1
GCA_030923225.1	GCA_030923225_1_ASM3092322v1	NCBI	2019	Asia	China	China:Taiwan	3	P1-1	6	6.1	p1-1, L6
GCA_030923245.1	GCA_030923245_1_ASM3092324v1	NCBI	1992	Asia	Japan	Japan	17	P1-1	4	4.2	p1-1
GCA_030923285.1	GCA_030923285_1_ASM3092328v1	NCBI	2019	Asia	China	China:Taiwan	3	P1-1	6	6.2	p1-1, L6
GCA_030923305.1	GCA_030923305_1_ASM3092330v1	NCBI	2019	Asia	China	China:Taiwan	3	P1-1	6	6.1	p1-1, L6
GCA_030923315.1	GCA_030923315_1_ASM3092331v1	NCBI	2019	Asia	China	China:Taiwan	17	P1-1	4	4.3	p1-1
GCA_030923325.1	GCA_030923325_1_ASM3092332v1	NCBI	2019	Asia	China	China:Taiwan	3	P1-1	6	6.1	p1-1, L6
GCA_030923335.1	GCA_030923335_1_ASM3092333v1	NCBI	2019	Asia	China	China:Taiwan	17	P1-1	4	4.3	p1-1
GCA_030923395.1	GCA_030923395_1_ASM3092339v1	NCBI	2019	Asia	China	China:Taiwan	3	P1-1	6	6.1	p1-1, L6

GCA_030923415.1	GCA_030923415_1_ASM3092341v1	NCBI	2019	Asia	China	China:Taiwan	3	P1-1	6	6.1	p1-1, L6
GCA_030923435.1	GCA_030923435_1_ASM3092343v1	NCBI	2018	Asia	China	China:Taiwan	17	P1-1	4	4.4	p1-1
GCA_030923455.1	GCA_030923455_1_ASM3092345v1	NCBI	1985	Asia	Japan	Japan	14	not available	2	2.1	p1-2, L2
GCA_030923475.1	GCA_030923475_1_ASM3092347v1	NCBI	2018	Asia	China	China:Taiwan	17	P1-1	4	4.4	p1-1
GCA_030923495.1	GCA_030923495_1_ASM3092349v1	NCBI	2018	Asia	China	China:Taiwan	3	P1-1	6	6.1	p1-1, L6
GCA_030923515.1	GCA_030923515_1_ASM3092351v1	NCBI	2018	Asia	China	China:Taiwan	3	P1-1	6	6.1	p1-1, L6
GCA_030923535.1	GCA_030923535_1_ASM3092353v1	NCBI	2019	Asia	China	China:Taiwan	3	P1-1	6	6.1	p1-1, L6
GCA_030923555.1	GCA_030923555_1_ASM3092355v1	NCBI	2019	Asia	China	China:Taiwan	3	P1-1	6	6.2	p1-1, L6
GCA_030923575.1	GCA_030923575_1_ASM3092357v1	NCBI	2019	Asia	China	China:Taiwan	3	P1-1	6	6.1	p1-1, L6
GCA_030923585.1	GCA_030923585_1_ASM3092358v1	NCBI	2019	Asia	China	China:Taiwan	17	P1-1	4	4.4	p1-1
GCA_030923675.1	GCA_030923675_1_ASM3092367v1	NCBI	2019	Asia	China	China:Taiwan	17	P1-1	4	4.2	p1-1
GCA_030923695.1	GCA_030923695_1_ASM3092369v1	NCBI	2019	Asia	China	China:Taiwan	3	P1-1	6	6.1	p1-1, L6
GCA_030923755.1	GCA_030923755_1_ASM3092375v1	NCBI	2019	Asia	China	China:Taiwan	3	P1-1	6	6.1	p1-1, L6
GCA_030923795.1	GCA_030923795_1_ASM3092379v1	NCBI	2019	Asia	China	China:Taiwan	3	P1-1	6	6.1	p1-1, L6
GCA_030923825.1	GCA_030923825_1_ASM3092382v1	NCBI	2019	Asia	China	China:Taiwan	3	P1-1	6	6.1	p1-1, L6
GCA_030923835.1	GCA_030923835_1_ASM3092383v1	NCBI	2019	Asia	China	China:Taiwan	3	P1-1	6	6.1	p1-1, L6
GCA_030923875.1	GCA_030923875_1_ASM3092387v1	NCBI	2019	Asia	China	China:Taiwan	3	P1-1	6	6.1	p1-1, L6
GCA_030923885.1	GCA_030923885_1_ASM3092388v1	NCBI	2019	Asia	China	China:Taiwan	17	P1-1	4	4.2	p1-1
GCA_030924015.1	GCA_030924015_1_ASM3092401v1	NCBI	2019	Asia	China	China:Taiwan	3	P1-1	6	6.1	p1-1, L6
GCA_030924025.1	GCA_030924025_1_ASM3092402v1	NCBI	2019	Asia	China	China:Taiwan	3	P1-1	6	6.1	p1-1, L6
GCA_030924065.1	GCA_030924065_1_ASM3092406v1	NCBI	2019	Asia	China	China:Taiwan	3	P1-1	6	6.1	p1-1, L6
GCA_030924095.1	GCA_030924095_1_ASM3092409v1	NCBI	2019	Asia	China	China:Taiwan	17	P1-1	4	4.2	p1-1
GCA_030924105.1	GCA_030924105_1_ASM3092410v1	NCBI	2019	Asia	China	China:Taiwan	17	P1-1	4	4.3	p1-1
GCA_030924135.1	GCA_030924135_1_ASM3092413v1	NCBI	2019	Asia	China	China:Taiwan	3	not available	6	6.1	p1-1, L6
GCA_030924155.1	GCA_030924155_1_ASM3092415v1	NCBI	2020	Asia	China	China:Taiwan	3	P1-1	6	6.1	p1-1, L6
GCA_030924175.1	GCA_030924175_1_ASM3092417v1	NCBI	2019	Asia	China	China:Taiwan	3	P1-1	6	6.1	p1-1, L6
GCA_030924195.1	GCA_030924195_1_ASM3092419v1	NCBI	2019	Asia	China	China:Taiwan	3	P1-1	6	6.1	p1-1, L6
GCA_030924205.1	GCA_030924205_1_ASM3092420v1	NCBI	2019	Asia	China	China:Taiwan	17	P1-1	4	4.2	p1-1
GCA_030924235.1	GCA_030924235_1_ASM3092423v1	NCBI	2020	Asia	China	China:Taiwan	17	P1-1	4	4.2	p1-1
GCA_030924255.1	GCA_030924255_1_ASM3092425v1	NCBI	2020	Asia	China	China:Taiwan	3	P1-1	6	6.1	p1-1, L6
GCA_030924265.1	GCA_030924265_1_ASM3092426v1	NCBI	2020	Asia	China	China:Taiwan	3	P1-1	6	6.1	p1-1, L6
GCA_030924275.1	GCA_030924275_1_ASM3092427v1	NCBI	2020	Asia	China	China:Taiwan	17	P1-1	4	4.2	p1-1
GCA_030924315.1	GCA_030924315_1_ASM3092431v1	NCBI	2020	Asia	China	China:Taiwan	14	P1-2	2	2.2	p1-2, L2
GCA_030924335.1	GCA_030924335_1_ASM3092433v1	NCBI	2019	Asia	China	China:Taiwan	3	P1-1	6	6.2	p1-1, L6
GCA_030924355.1	GCA_030924355_1_ASM3092435v1	NCBI	2019	Asia	China	China:Taiwan	3	P1-1	6	6.2	p1-1, L6
GCA_030924375.1	GCA_030924375_1_ASM3092437v1	NCBI	2019	Asia	China	China:Taiwan	17	P1-1	4	4.4	p1-1
GCA_030924395.1	GCA_030924395_1_ASM3092439v1	NCBI	2019	Asia	China	China:Taiwan	3	P1-1	6	6.1	p1-1, L6
GCA_030924405.1	GCA_030924405_1_ASM3092440v1	NCBI	2019	Asia	China	China:Taiwan	3	P1-1	6	6.1	p1-1, L6
GCA_030924435.1	GCA_030924435_1_ASM3092443v1	NCBI	2019	Asia	China	China:Taiwan	17	P1-1	4	4.2	p1-1
GCA_030924455.1	GCA_030924455_1_ASM3092445v1	NCBI	2019	Asia	China	China:Taiwan	3	P1-1	6	6.2	p1-1, L6
GCA_030924475.1	GCA_030924475_1_ASM3092447v1	NCBI	2019	Asia	China	China:Taiwan	3	P1-1	6	6.2	p1-1, L6
GCA_030924495.1	GCA_030924495_1_ASM3092449v1	NCBI	2019	Asia	China	China:Taiwan	17	P1-1	4	4.4	p1-1
GCA_030924505.1	GCA_030924505_1_ASM3092450v1	NCBI	2019	Asia	China	China:Taiwan	3	P1-1	6	6.2	p1-1, L6
GCA_030924535.1	GCA_030924535_1_ASM3092453v1	NCBI	2019	Asia	China	China:Taiwan	17	P1-1	4	4.4	p1-1
GCA_030924545.1	GCA_030924545_1_ASM3092454v1	NCBI	2019	Asia	China	China:Taiwan	14	P1-2	2	2.2	p1-2, L2
GCA_030924575.1	GCA_030924575_1_ASM3092457v1	NCBI	2019	Asia	China	China:Taiwan	17	P1-1	4	4.4	p1-1
GCA_030924595.1	GCA_030924595_1_ASM3092459v1	NCBI	2019	Asia	China	China:Taiwan	3	P1-1	6	6.1	p1-1, L6
GCA_030924615.1	GCA_030924615_1_ASM3092461v1	NCBI	2019	Asia	China	China:Taiwan	3	P1-1	6	6.1	p1-1, L6
GCA_030924635.1	GCA_030924635_1_ASM3092463v1	NCBI	2019	Asia	China	China:Taiwan	3 like	P1-1	6	6.2	p1-1, L6
GCA_030924655.1	GCA_030924655_1_ASM3092465v1	NCBI	2019	Asia	China	China:Taiwan	14	not available	2	2.2	p1-2, L2
GCA_030924675.1	GCA_030924675_1_ASM3092467v1	NCBI	2019	Asia	China	China:Taiwan	3	P1-1	6	6.1	p1-1, L6
GCA_030924695.1	GCA_030924695_1_ASM3092469v1	NCBI	2019	Asia	China	China:Taiwan	17	P1-1	4	4.4	p1-1
GCA_030924705.1	GCA_030924705_1_ASM3092470v1	NCBI	2019	Asia	China	China:Taiwan	17	P1-1	4	4.2	p1-1
GCA_030924715.1	GCA_030924715_1_ASM3092471v1	NCBI	2019	Asia	China	China:Taiwan	3	P1-1	6	6.1	p1-1, L6
GCA_030924755.1	GCA_030924755_1_ASM3092475v1	NCBI	2019	Asia	China	China:Taiwan	3	P1-1	6	6.1	p1-1, L6
GCA_030924775.1	GCA_030924775_1_ASM3092477v1	NCBI	2019	Asia	China	China:Taiwan	3	P1-1	6	6.1	p1-1, L6
GCA_030924795.1	GCA_030924795_1_ASM3092479v1	NCBI	2019	Asia	China	China:Taiwan	3	P1-1	6	6.1	p1-1, L6
GCA_030924815.1	GCA_030924815_1_ASM3092481v1	NCBI	2019	Asia	China	China:Taiwan	3	P1-1	6	6.1	p1-1, L6
GCA_030924825.1	GCA_030924825_1_ASM3092482v1	NCBI	2019	Asia	China	China:Taiwan	3	P1-1	6	6.1	p1-1, L6
GCA_030924835.1	GCA_030924835_1_ASM3092483v1	NCBI	2019	Asia	China	China:Taiwan	14	P1-2	2	2.2	p1-2, L2

GCA_030924875.1	GCA_030924875_1_ASM3092487v1	NCBI	2019	Asia	China	China:Taiwan	3	P1-1	6	6.1	p1-1, L6
GCA_030924895.1	GCA_030924895_1_ASM3092489v1	NCBI	2019	Asia	China	China:Taiwan	3	P1-1	6	6.1	p1-1, L6
GCA_030924915.1	GCA_030924915_1_ASM3092491v1	NCBI	2019	Asia	China	China:Taiwan	17	P1-1	4	4.3	p1-1
GCA_030924925.1	GCA_030924925_1_ASM3092492v1	NCBI	2019	Asia	China	China:Taiwan	2	not available	1	1.2	p1-2
GCA_030924935.1	GCA_030924935_1_ASM3092493v1	NCBI	2019	Asia	China	China:Taiwan	3	P1-1	6	6.1	p1-1, L6
GCA_030924945.1	GCA_030924945_1_ASM3092494v1	NCBI	2019	Asia	China	China:Taiwan	17	P1-1	4	4.4	p1-1
GCA_030924995.1	GCA_030924995_1_ASM3092499v1	NCBI	2019	Asia	China	China:Taiwan	3	P1-1	6	6.1	p1-1, L6
GCA_030925015.1	GCA_030925015_1_ASM3092501v1	NCBI	2019	Asia	China	China:Taiwan	17	P1-1	4	4.2	p1-1
GCA_030925035.1	GCA_030925035_1_ASM3092503v1	NCBI	2018	Asia	China	China:Taiwan	3	P1-1	6	6.1	p1-1, L6
GCA_030925055.1	GCA_030925055_1_ASM3092505v1	NCBI	2018	Asia	China	China:Taiwan	3	P1-1	6	6.1	p1-1, L6
GCA_030925075.1	GCA_030925075_1_ASM3092507v1	NCBI	2018	Asia	China	China:Taiwan	14	P1-2	2	2.2	p1-2, L2
GCA_030925085.1	GCA_030925085_1_ASM3092508v1	NCBI	2018	Asia	China	China:Taiwan	17	P1-1	4	4.4	p1-1
GCA_030925115.1	GCA_030925115_1_ASM3092511v1	NCBI	2018	Asia	China	China:Taiwan	17	P1-1	4	4.4	p1-1
GCA_030925135.1	GCA_030925135_1_ASM3092513v1	NCBI	2019	Asia	China	China:Taiwan	17	P1-1	4	4.3	p1-1
GCA_030925155.1	GCA_030925155_1_ASM3092515v1	NCBI	2018	Asia	China	China:Taiwan	17	P1-1	4	4.4	p1-1
GCA_030925165.1	GCA_030925165_1_ASM3092516v1	NCBI	2018	Asia	China	China:Taiwan	17	P1-1	4	4.4	p1-1
GCA_030925175.1	GCA_030925175_1_ASM3092517v1	NCBI	2018	Asia	China	China:Taiwan	3	P1-1	6	6.1	p1-1, L6
GCA_030925215.1	GCA_030925215_1_ASM3092521v1	NCBI	2020	Asia	China	China:Taiwan	3	P1-1	6	6.2	p1-1, L6
GCA_030925235.1	GCA_030925235_1_ASM3092523v1	NCBI	2019	Asia	China	China:Taiwan	17	P1-1	4	4.3	p1-1
GCA_030925245.1	GCA_030925245_1_ASM3092524v1	NCBI	2019	Asia	China	China:Taiwan	3	P1-1	6	6.1	p1-1, L6
GCA_030925255.1	GCA_030925255_1_ASM3092525v1	NCBI	2020	Asia	China	China:Taiwan	17	P1-1	4	4.2	p1-1
GCA_030925295.1	GCA_030925295_1_ASM3092529v1	NCBI	2019	Asia	China	China:Taiwan	3	P1-1	6	6.1	p1-1, L6
GCA_030925315.1	GCA_030925315_1_ASM3092531v1	NCBI	2019	Asia	China	China:Taiwan	3	P1-1	6	6.1	p1-1, L6
GCA_030925335.1	GCA_030925335_1_ASM3092533v1	NCBI	2019	Asia	China	China:Taiwan	3	P1-1	6	6.2	p1-1, L6
GCA_030925355.1	GCA_030925355_1_ASM3092535v1	NCBI	2018	Asia	China	China:Taiwan	3	P1-1	6	6.1	p1-1, L6
GCA_030925375.1	GCA_030925375_1_ASM3092537v1	NCBI	2019	Asia	China	China:Taiwan	3	P1-1	6	6.1	p1-1, L6
GCA_030925385.1	GCA_030925385_1_ASM3092538v1	NCBI	2019	Asia	China	China:Taiwan	3	P1-1	6	6.2	p1-1, L6
GCA_030928045.1	GCA_030928045_1_ASM3092804v1	NCBI	2019	Asia	China	China:Taiwan	3	P1-1	6	6.1	p1-1, L6
GCA_900660465.1	GCA_900660465_1_50648_A01-3	NCBI	1967				2	P1-2	1	1.1	p1-2
GCA_910574535.1	GCA_910574535_1_mpn_CV2	NCBI					1	P1-1	3	3.1	

23s rRNA mutation sites						Function	NUM_FOUND	dherenc	dherenc	dherenc Cytadherence organelle	\dherenc	dherenc	dherenc	dheren	\dherenc PDH-B	dherenc EF-	Invasio P200	Exotoxin ARDS toxin
A2063G	A2063C	A2063T	A2064 C	A2064 G	C2617 G	Virulence factor		hmw1	hmw2	hmw3	/p90/mg	MgPa/g	p30/p32	p65	pdhB	tuf	p200	?N_RS02090
100	0	0	0	0	0		8	100	100	100	0	0	100	100	100	100	100	100
100	0	0	0	0	0		10	100	100	100	100	100	100	100	100	100	100	100
100	0	0	0	0	0		10	100	100	100	100	100	100	100	100	100	100	100
100	0	0	0	0	0		10	100	100	100	100	100	89.7	100	100	100	100	100
100	0	0	0	0	0		10	100	100	100	100	100	100	100	100	100	100	100
100	0	0	0	0	0		10	100	100	100	100	100	89.7	100	100	100	100	100
100	0	0	0	0	0		10	100	100	100	100	100	100	100	100	100	100	100
0	0	0	0	0	0		10	100	100	100	100	99.92	89.7	100	100	100	100	100
100	0	0	0	0	0		10	100	100	100	100	100	89.7	100	100	100	100	100
100	0	0	0	0	0		10	100	100	100	100	100	89.7	100	100	100	100	100
100	0	0	0	0	0		10	100	100	100	100	100	89.7	100	100	100	100	100
100	0	0	0	0	0		10	100	100	100	100	100	89.7	100	100	100	100	100
100	0	0	0	0	0		10	100	100	100	100	100	89.7	100	100	100	100	100
0	0	0	0	0	0		9	100	100	100	0	83.11	100	100	100	100	100	100
100	0	0	0	0	0		10	100	100	100	100	100	89.7	100	100	100	100	100
100	0	0	0	0	0		8	100	100	100	0	0	100	100	100	100	100	100
100	0	0	0	0	0		10	100	100	100	100	100	89.7	100	100	100	100	100
100	0	0	0	0	0		10	100	100	100	100	100	89.7	100	100	100	100	100
100	0	0	0	0	0		8	100	100	100	0	0	100	100	100	100	100	100
100	0	0	0	0	0		10	100	100	100	100	100	100	100	100	100	100	100
0	0	0	0	0	0		9	100	100	100	0	83.11	100	100	100	100	100	100
0	0	0	0	0	0		9	100	100	100	0	83.11	100	100	100	100	100	100
100	0	0	0	0	0		10	100	100	100	100	100	89.7	100	100	100	100	100
100	0	0	0	0	0		10	100	100	100	100	100	89.7	100	100	100	100	100
100	0	0	0	0	0		10	100	100	100	100	100	100	100	100	100	100	100
100	0	0	0	0	0		10	100	100	100	100	100	89.7	100	100	100	100	100
100	0	0	0	0	0		10	100	100	100	100	100	89.7	100	100	100	100	100
100	0	0	0	0	0		9	100	100	100	0	99.61	100	100	100	100	100	100
100	0	0	0	0	0		10	100	100	100	100	100	89.7	100	100	100	100	100
100	0	0	0	0	0		10	100	100	100	100	99.94	100	100	100	100	100	100
100	0	0	0	0	0		10	100	100	100	100	100	89.7	100	100	100	100	100
100	0	0	0	0	0		10	100	100	100	100	100	89.7	100	100	100	100	100
100	0	0	0	0	0		10	100	100	100	100	100	89.7	100	100	100	100	100
100	0	0	0	0	0		10	100	100	100	100	100	89.7	100	100	100	100	100
100	0	0	0	0	0		10	100	100	100	100	100	100	100	100	100	100	100
100	0	0	0	0	0		10	100	100	100	100	100	89.7	100	100	100	100	100
100	0	0	0	0	0		8	100	100	100	0	99.22	100	0	100	100	100	100
100	0	0	0	0	0		10	100	100	100	100	100	100	100	100	100	100	100
100	0	0	0	0	0		10	100	100	100	100	100	89.7	100	100	100	100	100
100	0	0	0	0	0		10	100	100	100	100	100	100	100	100	100	100	100
100	0	0	0	0	0		10	100	100	100	100	100	100	100	100	100	100	100
100	0	0	0	0	0		10	100	100	100	100	100	100	100	100	100	100	100
100	0	0	0	0	0		10	100	100	100	100	100	100	100	100	100	100	100
100	0	0	0	0	0		10	100	100	100	100	100	100	100	100	100	100	100
100	0	0	0	0	0		10	100	100	100	100	100	89.7	100	100	100	100	100
100	0	0	0	0	0		10	100	100	100	100	100	89.7	100	100	100	100	100
100	0	0	0	0	0		10	100	100	100	100	100	100	100	100	100	100	100
100	0	0	0	0	0		10	100	100	100	100	100	89.7	100	100	100	100	100
100	0	0	0	0	0		10	100	100	100	100	100	89.7	100	100	100	100	100
100	0	0	0	0	0		10	100	100	100	100	100	100	100	100	100	100	100
100	0	0	0	0	0		10	100	100	100	100	100	89.7	100	100	100	100	100
100	0	0	0	0	0		10	100	100	100	100	100	89.7	100	100	100	100	100
100	0	0	0	0	0		10	100	100	100	100	100	89.7	100	100	100	100	100
100	0	0	0	0	0		10	100	100	100	100	99.94	100	100	100	100	100	100

100	0	0	0	0	0	10	100	100	100	100	100	89.7	100	100	100	100	100
100	0	0	0	0	0	10	100	100	100	100	99.94	100	100	100	100	100	100
100	0	0	0	0	0	10	100	100	100	100	100	100	100	100	100	100	100
100	0	0	0	0	0	8	100	100	100	0	0	100	100	100	100	100	100
100	0	0	0	0	0	10	100	100	100	100	100	89.7	100	100	100	100	100
100	0	0	0	0	0	10	100	100	100	100	100	89.7	100	100	100	100	100
100	0	0	0	0	0	10	100	100	100	100	100	89.7	100	100	100	100	100
100	0	0	0	0	0	10	100	100	100	100	100	89.7	100	100	100	100	100
100	0	0	0	0	0	8	100	100	100	0	0	100	100	100	100	100	100
100	0	0	0	0	0	9	100	100	100	0	99.61	100	100	100	100	100	100
100	0	0	0	0	0	10	100	100	100	100	100	100	100	100	100	100	100
100	0	0	0	0	0	10	100	100	100	100	100	89.7	100	100	100	100	100
100	0	0	0	0	0	10	100	100	100	100	100	89.7	100	100	100	100	100
0	0	0	0	0	0	10	100	100	100	100	100	100	100	100	100	100	100
100	0	0	0	0	0	10	100	100	100	100	99.94	89.7	100	100	100	100	100
100	0	0	0	0	0	10	100	100	100	100	99.94	100	100	100	100	100	100
100	0	0	0	0	0	10	100	100	100	100	100	100	100	100	100	100	100
100	0	0	0	0	0	10	100	100	100	100	100	89.7	100	100	100	100	100
100	0	0	0	0	0	8	100	100	100	0	0	100	100	100	100	100	100
100	0	0	0	0	0	10	100	100	100	100	100	89.7	100	100	100	100	100
100	0	0	0	0	0	8	100	100	100	0	0	100	100	100	100	100	100
100	0	0	0	0	0	10	100	100	100	100	100	100	100	100	100	100	100
100	0	0	0	0	0	10	100	100	100	100	100	89.7	100	100	100	100	100
100	0	0	0	0	0	10	100	100	100	100	99.94	89.7	100	100	100	100	100
100	0	0	0	0	0	10	100	100	100	100	100	89.7	100	100	100	100	100
100	0	0	0	0	0	9	100	100	100	0	99.61	100	100	100	100	100	100
100	0	0	0	0	0	10	100	100	100	100	100	89.7	100	100	100	100	100
100	0	0	0	0	0	10	100	100	100	100	100	89.7	100	100	100	100	100
100	0	0	0	0	0	10	100	100	100	100	100	89.7	100	100	100	100	100
100	0	0	0	0	0	10	100	100	100	100	100	100	100	100	100	100	100
100	0	0	0	0	0	10	100	100	100	100	100	89.7	100	100	100	100	100
100	0	0	0	0	0	10	100	100	100	100	100	89.7	100	100	100	100	100
100	0	0	0	0	0	10	100	100	100	100	100	100	100	100	100	100	100
100	0	0	0	0	0	10	100	100	100	100	100	89.7	100	100	100	100	100
100	0	0	0	0	0	10	100	100	100	100	99.94	89.7	100	100	100	100	100
100	0	0	0	0	0	10	100	100	100	100	100	89.7	100	100	100	100	100
100	0	0	0	0	0	8	100	100	100	0	0	100	100	100	100	100	100
100	0	0	0	0	0	10	100	100	100	100	100	89.7	100	100	100	100	100
100	0	0	0	0	0	10	100	100	100	100	100	89.7	100	100	100	100	100
100	0	0	0	0	0	10	100	100	100	100	100	89.7	100	100	100	100	100
100	0	0	0	0	0	10	100	100	100	100	100	89.7	100	100	100	100	100
100	0	0	0	0	0	10	100	100	100	100	100	89.7	100	100	100	100	100
100	0	0	0	0	0	10	100	100	100	100	100	89.7	100	100	100	100	100
100	0	0	0	0	0	10	100	100	100	100	100	89.7	100	100	100	100	100
100	0	0	0	0	0	10	100	100	100	100	100	89.7	100	100	100	100	100
100	0	0	0	0	0	9	100	100	100	0	99.61	100	100	100	100	100	100
100	0	0	0	0	0	10	100	100	100	100	100	89.7	100	100	100	100	100
100	0	0	0	0	0	10	100	100	100	100	100	100	100	100	100	100	100
100	0	0	0	0	0	10	100	100	100	100	100	89.7	100	100	100	100	100
100	0	0	0	0	0	10	100	100	100	100	100	89.7	100	100	100	100	100
100	0	0	0	0	0	10	100	100	100	100	100	89.7	100	100	100	100	100
100	0	0	0	0	0	10	100	100	100	100	100	89.7	100	100	100	100	100
100	0	0	0	0	0	10	100	100	100	100	100	89.7	100	100	100	100	100
100	0	0	0	0	0	10	100	100	100	100	100	89.7	100	100	100	100	100
100	0	0	0	0	0	10	100	100	100	100	99.92	89.7	100	100	100	100	100
100	0	0	0	0	0	10	100	100	100	100	99.94	89.7	100	100	100	100	100
100	0	0	0	0	0	10	100	100	100	100	100	89.7	100	100	100	100	100
100	0	0	0	0	0	10	100	100	100	100	100	100	100	100	100	100	100
100	0	0	0	0	0	10	100	100	100	100	100	89.7	100	100	100	100	100
100	0	0	0	0	0	10	100	100	100	100	100	100	100	100	100	100	100

100	0	0	0	0	0	10	100	100	100	100	100	89.7	100	100	100	100	100
100	0	0	0	0	0	10	100	100	100	100	100	100	100	100	100	100	100
100	0	0	0	0	0	10	100	100	100	100	100	89.7	100	100	100	100	100
100	0	0	0	0	0	10	100	100	100	100	100	100	100	100	100	100	100
100	0	0	0	0	0	10	100	100	100	100	100	89.7	100	100	100	100	100
100	0	0	0	0	0	10	100	100	100	100	100	89.7	100	100	100	100	100
100	0	0	0	0	0	10	100	100	100	100	100	100	100	100	100	100	100
100	0	0	0	0	0	8	100	100	100	0	0	100	100	100	100	100	100
100	0	0	0	0	0	10	100	100	100	100	100	100	100	100	100	100	100
100	0	0	0	0	0	10	100	100	100	100	100	89.7	100	100	100	100	100
100	0	0	0	0	0	9	100	100	100	0	99.61	100	100	100	100	100	100
100	0	0	0	0	0	8	100	100	100	0	0	100	100	100	100	100	100
100	0	0	0	0	0	8	100	100	100	0	0	100	100	100	100	100	100
100	0	0	0	0	0	10	100	100	100	100	100	100	100	100	100	100	100
100	0	0	0	0	0	10	100	100	100	100	100	89.7	100	100	100	100	100
100	0	0	0	0	0	10	100	100	100	100	100	89.7	100	100	100	100	100
100	0	0	0	0	0	10	100	100	100	100	100	89.7	100	100	100	100	100
100	0	0	0	0	0	8	100	100	100	0	0	100	100	100	100	100	100
100	0	0	0	0	0	8	100	100	100	0	0	100	100	100	100	100	100
100	0	0	0	0	0	10	100	100	100	100	100	100	100	100	100	100	100
100	0	0	0	0	0	10	100	100	100	100	100	89.7	100	100	100	100	100
100	0	0	0	0	0	10	100	100	100	100	100	100	100	100	100	100	100
100	0	0	0	0	0	8	100	100	100	0	0	100	100	100	100	100	100
100	0	0	0	0	0	10	100	100	100	100	100	89.7	100	100	100	100	100
100	0	0	0	0	0	10	100	100	100	100	100	89.7	100	100	100	100	100
100	0	0	0	0	0	10	100	100	100	100	100	100	100	100	100	100	100
100	0	0	0	0	0	10	100	100	100	100	100	89.7	100	100	100	100	100
100	0	0	0	0	0	8	100	100	100	0	0	100	100	100	100	100	100
100	0	0	0	0	0	8	100	100	100	0	0	100	100	100	100	100	100
100	0	0	0	0	0	10	100	100	100	100	100	100	100	100	100	100	100
100	0	0	0	0	0	10	100	100	100	100	100	100	100	100	100	100	100
100	0	0	0	0	0	10	100	100	100	100	100	89.7	100	100	100	100	100
100	0	0	0	0	0	10	100	100	100	100	100	89.7	100	100	100	100	100
100	0	0	0	0	0	8	100	100	100	0	0	100	100	100	100	100	100
100	0	0	0	0	0	10	100	100	100	100	100	100	100	100	100	100	100
100	0	0	0	0	0	8	100	100	100	0	0	100	100	100	100	100	100
100	0	0	0	0	0	10	100	100	100	100	100	89.7	100	100	100	100	100
100	0	0	0	0	0	10	100	100	100	100	100	100	100	100	100	100	100
100	0	0	0	0	0	8	100	100	100	0	0	100	100	100	100	100	100
100	0	0	0	0	0	10	100	100	100	100	100	89.7	100	100	100	100	100
100	0	0	0	0	0	10	100	100	100	100	100	89.7	100	100	100	100	100
100	0	0	0	0	0	10	100	100	100	100	100	89.7	100	100	100	100	100
100	0	0	0	0	0	10	100	100	100	100	100	89.7	100	100	100	100	100
100	0	0	0	0	0	10	100	100	100	100	100	89.7	100	100	100	100	100
100	0	0	0	0	0	10	100	100	100	100	100	100	100	100	100	100	100
100	0	0	0	0	0	10	100	100	100	100	100	89.7	100	100	100	100	100
100	0	0	0	0	0	9	100	100	100	0	99.28	100	100	100	100	100	100
100	0	0	0	0	0	10	100	100	100	100	100	89.7	100	100	100	100	100
100	0	0	0	0	0	10	100	100	100	100	100	100	100	100	100	100	100
100	0	0	0	0	0	10	100	100	100	100	100	100	100	100	100	100	100
100	0	0	0	0	0	10	100	100	100	100	100	100	100	100	100	100	100
100	0	0	0	0	0	10	100	100	100	100	100	100	100	100	100	100	100
100	0	0	0	0	0	10	100	100	100	100	100	89.7	100	100	100	100	100
100	0	0	0	0	0	10	100	100	100	100	100	89.7	100	100	100	100	100
100	0	0	0	0	0	10	100	100	100	100	100	100	100	100	100	100	100
100	0	0	0	0	0	10	100	100	100	100	100	100	100	100	100	100	100
100	0	0	0	0	0	10	100	100	100	100	100	89.7	100	100	100	100	100
100	0	0	0	0	0	10	100	100	100	100	99.98	89.7	100	100	100	100	100
100	0	0	0	0	0	10	100	100	100	100	100	89.7	100	100	100	100	100
0	0	100	0	0	0	10	100	100	100	100	100	89.7	100	100	100	100	100

100	0	0	0	0	0	10	100	100	100	100	100	100	100	100	100	100	100
100	0	0	0	0	0	10	100	100	100	100	100	100	100	100	100	100	100
100	0	0	0	0	0	10	100	100	100	100	100	100	100	100	100	100	100
100	0	0	0	0	0	10	100	100	100	100	100	89.7	100	100	100	100	100
100	0	0	0	0	0	10	100	100	100	100	99.94	89.7	100	100	100	100	100
100	0	0	0	0	0	10	100	100	100	100	100	89.7	100	100	100	100	100
100	0	0	0	0	0	10	100	100	100	100	100	100	100	100	100	100	100
100	0	0	0	0	0	10	100	100	100	100	99.94	100	100	100	100	100	100
100	0	0	0	0	0	10	100	100	100	100	100	89.7	100	100	100	100	100
100	0	0	0	0	0	10	100	100	100	100	100	100	100	100	100	100	100
100	0	0	0	0	0	10	100	100	100	100	100	89.7	100	100	100	100	100
0	0	0	0	0	0	10	100	100	100	100	100	100	100	100	100	100	100
100	0	0	0	0	0	10	100	100	100	100	100	89.7	100	100	100	100	100
100	0	0	0	0	0	8	100	100	100	0	0	100	100	100	100	100	100
100	0	0	0	0	0	10	100	100	100	100	100	89.7	100	100	100	100	100
100	0	0	0	0	0	10	100	100	100	100	100	89.7	100	100	100	100	100
100	0	0	0	0	0	8	100	100	100	0	0	100	100	100	100	100	100
100	0	0	0	0	0	10	100	100	100	100	100	89.7	100	100	100	100	100
100	0	0	0	0	0	8	100	100	100	0	0	100	100	100	100	100	100
100	0	0	0	0	0	10	100	100	100	100	100	89.7	100	100	100	100	100
100	0	0	0	0	0	10	100	100	100	100	100	100	100	100	100	100	100
100	0	0	0	0	0	9	100	100	0	100	100	89.7	100	100	100	100	100
100	0	0	0	0	0	10	100	100	100	100	100	89.7	100	100	100	100	100
100	0	0	0	0	0	10	100	100	100	100	99.94	100	100	100	100	100	100
100	0	0	0	0	0	10	100	100	100	100	100	89.7	100	100	100	100	100
100	0	0	0	0	0	9	100	100	100	0	99.22	100	100	100	100	100	100
100	0	0	0	0	0	10	100	100	100	100	100	89.7	100	100	100	100	100
100	0	0	0	0	0	10	100	100	100	100	100	89.7	100	100	100	100	100
100	0	0	0	0	0	8	100	100	100	0	0	100	100	100	100	100	100
100	0	0	0	0	0	10	100	100	100	100	100	100	100	100	100	100	100
100	0	0	0	0	0	10	100	100	100	100	99.92	89.7	100	100	100	100	100
100	0	0	0	0	0	10	100	100	100	100	100	100	100	100	100	100	100
100	0	0	0	0	0	10	100	100	100	100	100	89.7	100	100	100	100	100
100	0	0	0	0	0	10	100	100	100	100	99.88	100	100	100	100	100	100
100	0	0	0	0	0	10	100	100	100	100	100	89.7	100	100	100	100	100
100	0	0	0	0	0	10	100	100	100	100	100	89.7	100	100	100	100	100
100	0	0	0	0	0	10	100	100	100	100	100	89.7	100	100	100	100	100
100	0	0	0	0	0	10	100	100	100	100	100	100	100	100	100	100	100
100	0	0	0	0	0	10	100	100	100	100	100	89.7	100	100	100	100	100
100	0	0	0	0	0	10	100	100	100	100	100	89.7	100	100	100	100	100
100	0	0	0	0	0	10	100	100	100	100	100	89.7	100	100	100	100	100
100	0	0	0	0	0	8	100	100	100	0	0	100	100	100	100	100	100
100	0	0	0	0	0	10	100	100	100	100	100	89.7	100	100	100	100	100
100	0	0	0	0	0	10	100	100	100	100	100	89.7	100	100	100	100	100
100	0	0	0	0	0	10	100	100	100	100	100	89.7	100	100	100	100	100
100	0	0	0	0	0	10	100	100	100	100	100	100	100	100	100	100	100
100	0	0	0	0	0	10	100	100	100	100	100	89.7	100	100	100	100	100
100	0	0	0	0	0	10	100	100	100	100	100	89.7	100	100	100	100	100
100	0	0	0	0	0	10	100	100	100	100	100	100	100	100	100	100	100
100	0	0	0	0	0	10	100	100	100	100	100	89.7	100	100	100	100	100
100	0	0	0	0	0	9	100	100	100	0	99.61	100	100	100	100	100	100
100	0	0	0	0	0	10	100	100	100	100	100	100	100	100	100	100	100
100	0	0	0	0	0	10	100	100	100	100	100	89.7	100	100	100	100	100
100	0	0	0	0	0	10	100	100	100	100	100	100	100	100	100	100	100
100	0	0	0	0	0	10	100	100	100	100	100	100	100	100	100	100	100
100	0	0	0	0	0	10	100	100	100	100	100	100	100	100	100	100	100
100	0	0	0	0	0	8	100	100	100	0	0	100	100	100	100	100	100
100	0	0	0	0	0	10	100	100	100	100	99.88	100	100	100	100	100	100
100	0	0	0	0	0	10	100	100	100	100	100	89.7	100	100	100	100	100
100	0	0	0	0	0	10	100	100	100	100	100	100	100	100	100	100	100

100	0	0	0	0	0	10	100	100	100	100	100	89.7	100	100	100	100	100
100	0	0	0	0	0	10	100	100	100	100	100	100	100	100	100	100	100
100	0	0	0	0	0	10	100	100	100	100	100	100	100	100	100	100	100
100	0	0	0	0	0	10	100	100	100	100	100	89.7	100	100	100	100	100
100	0	0	0	0	0	10	100	100	100	100	100	89.7	100	100	100	100	100
100	0	0	0	0	0	10	100	100	100	100	100	89.7	100	100	100	100	100
100	0	0	0	0	0	10	100	100	100	100	100	89.7	100	100	100	100	100
100	0	0	0	0	0	10	100	100	100	100	100	89.7	100	100	100	100	100
100	0	0	0	0	0	10	100	100	100	100	100	100	100	100	100	100	100
100	0	0	0	0	0	10	100	100	100	100	100	89.7	100	100	100	100	100
100	0	0	0	0	0	10	100	100	100	100	100	100	100	100	100	100	100
100	0	0	0	0	0	10	100	100	100	100	100	89.7	100	100	100	100	100
100	0	0	0	0	0	10	100	100	100	100	100	100	100	100	100	100	100
100	0	0	0	0	0	10	100	100	100	100	100	89.7	100	100	100	100	0
100	0	0	0	0	0	10	100	100	100	100	100	100	100	100	100	100	100
100	0	0	0	0	0	10	100	100	100	100	100	100	100	100	100	100	100
100	0	0	0	0	0	10	100	100	100	100	100	100	100	100	100	100	100
100	0	0	0	0	0	10	100	100	100	100	100	89.7	100	100	100	100	100
100	0	0	0	0	0	10	100	100	100	100	100	89.7	100	100	100	100	100
100	0	0	0	0	0	10	100	100	100	100	100	100	100	100	100	100	100
100	0	0	0	0	0	10	100	100	100	100	100	89.7	100	100	100	100	100
100	0	0	0	0	0	10	100	100	100	100	100	100	100	100	100	100	100
100	0	0	0	0	0	10	100	100	100	100	100	89.7	100	100	100	100	0
100	0	0	0	0	0	8	100	100	100	0	0	100	100	100	100	100	100
100	0	0	0	0	0	10	100	100	100	100	100	89.7	100	100	100	100	100
100	0	0	0	0	0	10	100	100	100	100	100	100	100	100	100	100	100
100	0	0	0	0	0	10	100	100	100	100	100	100	100	100	100	100	100
100	0	0	0	0	0	9	100	100	100	0	99.55	100	100	100	100	100	100
100	0	0	0	0	0	10	100	100	100	100	100	89.7	100	100	100	100	100
100	0	0	0	0	0	8	100	100	100	0	0	100	100	100	100	100	100
100	0	0	0	0	0	10	100	100	100	100	100	89.7	100	100	100	100	100
100	0	0	0	0	0	10	100	100	100	100	100	100	100	100	100	100	100
100	0	0	0	0	0	10	100	100	100	100	100	100	100	100	100	100	100
0	0	0	0	0	0	9	100	100	100	0	83.11	100	100	100	100	100	100
0	0	0	0	0	0	9	100	100	100	0	99.63	100	100	100	100	100	100
0	0	0	0	0	0	9	100	100	100	0	99.67	100	100	100	100	100	100
0	0	0	0	0	0	9	100	100	100	0	83.11	100	100	100	100	100	100
0	0	0	0	0	0	10	100	100	100	100	100	100	100	100	100	100	100
0	0	0	0	0	0	9	100	99.98	100	0	99.55	99.88	100	100	100	100	100
0	0	0	0	0	0	9	100	100	100	0	99.28	100	100	100	100	100	100
100		0	0	0	0	9	100	100	100	0	99.55	100	100	100	100	100	100
	0																
0	0	0	0	0	0	10	100	100	100	100	100	100	100	100	100	100	100
0	0	0	0	0	0	10	100	100	100	100	100	100	100	100	100	100	100
100	0	0	0	0	0	9	100	100	100	0	99.61	100	100	100	100	100	100
0	0	0	0	0	0	10	100	100	100	100	100	89.7	100	100	100	100	100
0	0	0	0	0	0	9	100	100	100	0	99.61	100	100	100	100	100	100
0	0	0	0	0	0	10	100	100	100	100	100	100	100	100	100	100	100
0	0	0	0	0	0	10	100	100	100	100	99.88	100	100	100	100	100	100
0	0	0	0	0	0	10	100	100	100	100	100	100	100	100	100	100	100
0	0	0	0	0	0	10	100	100	100	100	100	100	100	100	100	100	100
0	0	0	0	0	0	10	100	100	100	100	100	100	100	100	100	100	100
0	0	0	0	0	0	9	100	100	100	0	99.55	100	100	100	100	100	100
0	0	0	0	0	0	9	100	100	100	0	99.61	100	100	100	100	100	100
0	0	0	0	0	0	9	100	100	100	0	99.61	100	100	100	100	100	100
0	0	0	0	0	0	9	100	100	100	0	99.61	100	100	100	100	100	100
0	0	0	0	0	0	9	100	100	100	0	99.51	100	100	100	100	100	100
0	0	0	0	0	0	9	100	100	100	80.48	0	100	100	100	100	100	100
0	0	0	0	100	0	9	100	100	100	80.48	0	100	100	100	100	100	100

0	0	0	0	0	0	8	100	100	100	0	0	100	100	100	100	100	100
0	0	0	0	0	0	8	100	100	100	0	0	100	100	100	100	100	100
0	0	0	0	0	0	8	100	100	100	0	0	100	100	100	100	100	100
0	0	0	0	0	0	10	100	100	100	80.48	82.78	100	100	100	100	100	100
0	0	0	0	0	100	8	100	100	100	0	0	100	100	100	100	100	100
0	0	0	0	0	0	9	100	100	100	80.48	0	100	100	100	100	100	100
0	0	0	0	0	0	8	100	100	100	0	0	100	100	100	100	100	100
100	0	0	0	0	0	9	100	100	100	80.48	0	100	100	100	100	100	100
0	0	0	0	0	0	8	100	100	100	0	0	100	100	100	100	100	100
0	0	0	0	0	0	9	100	100	100	80.48	0	100	100	100	100	100	100
0	0	0	0	0	0	9	100	100	100	80.48	0	100	100	100	100	100	100
0	0	0	0	0	0	9	100	100	100	80.48	0	100	100	100	100	100	100
0	100	0	0	0	0	8	100	100	100	0	0	100	100	100	100	100	100
0	0	0	0	0	0	8	100	100	100	0	0	100	100	100	100	100	100
0	0	0	0	0	0	8	100	100	100	0	0	100	100	100	100	100	100
0	0	0	0	0	0	9	100	100	100	80.48	0	100	100	100	100	100	100
0	0	0	0	0	0	8	100	100	100	0	0	100	100	100	100	100	100
100	0	0	0	0	0	9	100	100	100	80.48	0	100	100	100	100	100	100
100	0	0	0	0	0	9	100	100	100	80.48	0	100	100	100	100	100	100
100	0	0	0	0	0	9	100	100	100	80.48	0	100	100	100	100	100	00.00;95.50
100	0	0	0	0	0	9	100	100	100	80.48	0	100	100	100	100	100	100
100	0	0	0	0	0	6	0	100	100	0	0	98.42	0	100	100	100	100
100	0	0	0	0	0	8	100	100	100	0	0	89.7	88.42	100	100	100	100
100	0	0	0	0	0	7	100	100	100	0	0	89.7	0	100	100	100	100
100	0	0	0	0	0	8	100	100	100	0	0	89.7	100	100	100	100	100
100	0	0	0	0	0	8	100	100	100	0	0	89.7	88.42	100	100	100	100
100	0	0	0	0	0	8	100	100	100	0	0	80.97	100	100	100	100	100
100	0	0	0	0	0	8	100	100	100	0	0	89.7	88.42	100	100	100	100
100	0	0	0	0	0	8	100	100	100	0	0	98.42	88.42	100	100	100	100
0	0	0	0	0	0	8	100	100	100	0	0	89.7	88.42	100	100	100	100
100	0	0	0	0	0	8	100	100	100	0	0	80.97	100	100	100	100	100
0	0	0	0	0	0	10	100	100	100	100	99.2	100	100	100	100	100	100
100	0	0	0	0	0	10	100	100	100	100	100	100	100	100	100	100	100
100	0	0	0	0	0	9	100	100	100	0	99.55	100	100	100	100	100	100
100	0	0	0	0	0	10	100	100	100	100	100	100	100	100	100	100	100
100	0	0	0	0	0	10	100	100	100	100	100	100	100	100	100	100	100
100	0	0	0	0	0	10	100	100	100	100	100	100	100	100	100	100	100
100	0	0	0	0	0	10	100	100	100	100	100	100	100	100	100	100	100
100	0	0	0	0	0	10	100	100	100	100	100	100	100	100	100	100	100
0	0	0	0	0	0	10	100	100	100	100	100	100	100	100	100	100	100
0	0	0	0	0	0	10	100	100	100	100	100	100	100	100	100	100	100
0	0	0	0	0	0	10	100	100	100	100	100	100	100	100	100	100	100
0	0	0	0	0	0	10	100	100	100	100	100	100	100	100	100	100	100
0	0	0	0	0	0	9	100	100	100	0	99.22	100	100	100	100	100	100
0	0	0	0	0	0	10	100	100	100	100	99.94	100	100	100	100	100	100
0	0	0	0	0	0	10	100	99.98	100	100	100	100	100	100	100	100	100
0	0	0	0	0	0	10	100	100	100	100	99.94	100	100	100	100	100	100
0	0	0	0	0	0	9	100	100	100	0	99.32	100	100	100	100	100	100
0	0	0	0	0	0	9	99.97	100	100	0	99.32	100	100	100	100	100	100
100	0	0	0	0	0	9	100	100	100	0	99.28	100	100	100	100	100	100
0	0	0	0	0	0	10	100	100	100	100	100	100	100	100	100	100	100
0	0	0	0	0	0	9	100	100	100	0	99.32	100	100	100	100	100	100
0	0	0	0	0	0	9	100	100	100	0	99.61	100	99.01	100	100	100	100
0	0	0	0	0	0	9	100	100	100	0	99.61	100	100	100	100	100	100
0	0	0	0	0	0	9	100	100	100	0	99.61	100	100	100	100	100	100
0	0	0	0	0	0	9	100	100	100	0	99.22	100	100	100	100	100	100
0	0	0	0	0	0	9	100	100	100	0	99.32	100	100	100	100	100	100
0	0	0	0	0	0	10	100	100	100	100	100	100	100	100	100	100	100
0	0	0	0	0	0	9	100	100	100	0	99.32	100	100	100	100	100	100
0	0	0	0	0	0	9	100	100	100	0	99.18	100	100	100	100	100	100

0	0	0	0	0	0	10	100	100	100	100	100	100	100	100	100	100	100
0	0	0	0	0	0	9	100	100	100	0	99.61	100	100	100	100	100	100
0	0	0	0	0	0	9	100	100	100	0	99.61	100	100	100	100	100	100
0	0	0	0	0	0	10	100	100	100	100	100	95.64	100	100	100	100	100
0	0	0	0	0	0	10	100	100	100	100	100	100	100	100	100	100	00.00;100.00
0	0	0	0	0	0	10	100	100	100	99.97	100	100	100	100	100	100	100
0	0	0	0	0	0	10	100	100	100	100	100	100	100	100	100	100	100
0	0	0	0	0	0	9	100	100	100	0	99.55	100	100	100	100	100	100
0	0	0	0	0	0	8	100	100	100	0	0	100	100	100	100	100	100
0	0	0	0	0	0	8	100	100	100	0	0	100	100	100	100	100	100
0	0	0	0	0	0	8	100	100	100	0	0	100	100	100	100	100	88.74
0	0	0	0	0	0	8	100	100	100	0	0	100	100	100	100	100	100
0	0	0	0	0	0	5	0	0	100	0	0	100	100	100	100	0	100
0	0	0	0	0	0	7	100	100	100	0	0	0	100	100	100	100	100
0	0	0	0	0	0	8	100	100	100	0	0	100	100	100	100	100	100
0	0	100	0	0	0	8	100	100	100	0	0	100	100	100	100	100	100
0	0	0	0	0	0	8	100	100	100	0	0	100	100	100	100	100	100
0	0	0	0	0	0	7	100	100	100	0	0	0	100	100	100	100	100
0	0	0	0	0	0	7	100	100	100	0	0	100	0	100	100	100	100
0	0	0	0	0	0	9	100	100	100	100	96.42	100	0	100	100	100	100
0	0	0	0	0	0	6	100	100	100	0	0	0	0	100	100	100	100
0	0	0	0	0	0	7	100	100	100	0	0	0	100	100	100	100	100
0	0	0	0	0	0	9	100	100	100	100	87.37	100	0	100	100	100	100
0	0	0	0	0	0	7	100	100	100	0	0	0	100	100	100	100	100
0	0	0	0	0	0	6	100	100	100	0	0	0	0	100	100	100	100
0	0	0	0	0	0	10	100	100	100	100	96.44	100	100	100	100	100	100
0	0	0	0	0	0	7	100	100	100	0	0	100	0	100	100	100	100
0	0	0	0	0	0	8	100	100	100	100	0	100	0	100	100	100	100
0	0	0	0	0	0	7	100	100	100	0	0	0	100	100	100	100	100
0	0	0	0	0	0	8	100	100	100	100	0	100	0	100	100	100	100
0	0	0	0	0	0	9	100	100	100	100	96.42	100	0	100	100	100	100
0	0	0	0	0	0	8	100	100	100	0	0	100	100	100	100	100	100
100	0	0	0	0	0	6	100	100	97.03	0	0	0	0	100	100	100	100
0	0	0	0	0	0	7	100	100	97.03	0	0	100	0	100	100	100	100
0	0	0	0	0	0	7	100	100	100	0	0	0	100	100	100	100	100
0	0	0	0	0	0	6	100	100	100	0	0	0	0	100	100	100	100
0	0	0	0	0	0	7	100	100	100	0	0	82.79	0	100	100	100	100
0	0	0	0	0	0	10	100	100	100	100	100	100	100	100	100	100	100
0	0	100	0	0	0	7	100	100	100	0	0	100	0	100	100	100	100
0	0	0	0	0	0	6	100	100	100	0	0	0	0	100	100	100	100
100	0	0	0	0	0	6	100	100	100	0	0	0	0	100	100	100	100
0	0	100	0	0	0	7	100	100	100	0	0	100	0	100	100	100	100
0	0	0	0	0	0	9	100	100	100	100	98.42	100	0	100	100	100	100
100	0	0	0	0	0	8	100	100	100	0	0	100	100	100	100	100	100
100	0	0	0	0	0	9	100	100	100	100	0	100	100	100	100	100	100
100	0	0	0	0	0	7	100	100	100	0	0	100	0	100	100	100	100
0	0	0	0	0	0	10	100	100	100	100	100	100	100	100	100	100	100
0	0	0	0	0	0	7	100	100	100	0	0	100	0	100	100	100	100
0	0	0	0	0	0	7	100	100	100	0	0	100	0	100	100	100	100
100	0	0	0	0	0	7	100	100	100	0	0	100	0	100	100	100	100
0	0	0	0	0	0	7	100	100	100	0	0	100	0	100	100	100	100
100	0	0	0	0	0	7	100	100	100	0	0	100	0	100	100	100	100
0	0	0	0	0	0	7	100	100	100	0	0	100	0	100	100	100	100
100	0	0	0	0	0	8	100	100	100	80.48	0	100	0	100	100	100	99.04
100	0	0	0	0	0	7	100	100	100	0	0	0	100	100	100	100	100
0	0	0	0	0	0	6	100	100	100	0	0	0	0	100	100	100	100
100	0	0	0	0	0	6	100	100	100	0	0	0	0	100	100	100	100
0	0	0	0	0	0	5	100	0	100	0	0	0	0	100	100	100	100
100	0	0	0	0	0	7	100	100	100	0	0	100	0	100	100	100	100
0	0	0	0	0	0	6	100	0	100	0	0	80.24	100	100	100	0	100

100	0	0	0	0	0	7	100	100	100	0	0	100	0	100	100	100	100
0	0	0	0	0	0	6	100	100	100	0	0	0	0	100	100	100	100
100	0	0	0	0	0	6	100	100	100	0	0	0	0	100	100	100	100
0	0	0	0	0	0	5	0	100	100	0	0	0	0	100	100	100	100
0	0	0	0	0	0	6	100	100	100	0	0	0	0	100	100	100	100
100	0	0	0	0	0	8	100	100	100	98	0	100	0	100	100	100	100
0	0	0	100	0	0	7	100	100	100	0	0	100	0	100	100	100	100
100	0	0	0	0	0	6	100	100	100	0	0	0	0	100	100	100	100
0	0	0	0	0	0	6	100	100	100	0	0	0	0	100	100	100	100
0	0	0	0	0	0	7	100	100	100	0	0	100	0	100	100	100	100
100	0	0	0	0	0	7	100	100	100	0	0	100	0	100	100	100	100
100	0	0	0	0	0	7	100	100	100	0	0	100	0	100	100	100	100
100	0	0	0	0	0	7	100	100	100	0	0	100	0	100	100	100	100
100	0	0	0	0	0	9	100	100	100	0	99.18	100	100	100	100	100	100
100	0	0	0	0	0	10	100	100	100	100	100	100	100	100	100	100	100
100	0	0	0	0	0	10	100	100	100	100	100	100	100	100	100	100	100
100	0	0	0	0	0	10	100	100	100	100	100	100	100	100	100	100	100
100	0	0	0	0	0	10	100	100	100	100	100	100	100	100	100	100	100
100	0	0	0	0	0	10	100	100	100	100	99.94	100	100	100	100	100	100
100	0	0	0	0	0	10	100	100	100	100	99.94	100	100	100	100	100	100
0	0	0	0	0	0	10	100	100	100	100	100	100	100	100	100	100	100
100	0	0	0	0	0	10	100	100	100	100	99.94	100	100	100	100	100	100
100	0	0	0	0	0	10	100	100	100	100	100	100	100	100	100	100	100
0	0	0	0	0	0	10	100	100	100	100	100	100	100	100	100	100	100
0	0	0	0	0	0	9	100	100	100	0	99.18	100	100	100	100	100	100
100	0	0	0	0	0	10	100	100	100	100	100	100	100	100	100	100	100
100	0	0	0	0	0	10	100	100	100	100	100	100	100	100	100	100	100
0	0	0	0	0	0	9	100	100	100	0	99.28	100	100	100	100	100	100
100	0	0	0	0	0	10	100	100	100	100	100	100	100	100	100	100	100
0	0	0	0	0	0	9	100	100	100	0	99.28	100	100	100	100	100	100
0	0	0	0	0	0	10	100	100	100	100	100	100	100	100	100	100	100
0	0	0	0	0	0	10	100	100	100	100	100	100	100	100	100	100	100
0	0	0	0	0	0	10	100	100	100	100	100	100	100	100	100	100	100
0	0	0	0	0	0	9	100	100	100	0	99.18	100	100	100	100	100	100
100	0	0	0	0	0	10	100	100	100	100	100	100	100	100	100	100	100
0	0	0	0	0	0	10	100	100	100	100	100	100	100	100	100	100	100
0	0	0	0	0	0	9	100	100	100	0	99.12	100	100	100	100	100	100
100	0	0	0	0	0	10	100	100	100	100	99.94	100	100	100	100	100	100
0	0	0	0	0	0	10	100	100	100	100	100	100	100	100	100	100	100
0	0	0	0	0	0	10	100	100	100	100	100	100	100	100	100	100	100
0	0	0	0	0	0	10	100	100	100	100	100	100	100	100	100	100	100
100	0	0	0	0	0	10	100	100	100	100	100	100	100	100	100	100	100
0	0	0	0	0	0	10	100	100	100	100	100	100	100	100	100	100	100
0	0	0	0	0	0	8	100	100	100	0	0	100	100	100	100	100	100
100	0	0	0	0	0	9	100	100	100	100	100	89.7	0	100	100	100	100
100	0	0	0	0	0	7	100	100	100	0	0	100	0	100	100	100	100
0	0	0	0	0	0	7	100	100	100	0	0	100	0	100	100	100	100
0	0	0	0	0	0	7	100	100	100	0	0	89.7	0	100	100	100	100
100	0	0	0	0	0	9	100	100	100	100	100	0	85.47	100	100	100	100
0	0	100	0	0	0	8	100	100	100	100	0	0	100	100	100	100	100
100	0	0	0	0	0	8	100	100	100	0	0	82.79	100	100	100	100	100
100	0	0	0	0	0	8	100	100	100	0	0	80.73	100	100	100	100	100
100	0	0	0	0	0	9	100	100	100	0	99.94	81.09	100	100	100	100	100
100	0	0	0	0	0	9	100	100	100	100	0	88.24	100	100	100	100	100
0	0	0	0	0	0	10	100	100	100	100	100	100	100	100	100	100	100
100	0	0	0	0	0	10	100	100	100	100	100	100	100	100	100	100	100
100	0	0	0	0	0	10	100	100	100	100	85.05	82.06	100	100	100	100	100
0	0	0	0	0	0	10	100	100	100	100	100	100	100	100	100	100	100
0	0	0	0	0	0	10	100	100	100	100	100	100	100	100	100	100	100
0	0	0	0	0	0	10	100	100	100	100	100	81.94	100	100	100	100	100

100	0	0	0	0	0	10	100	100	100	100	100	100	100	100	100	100	100
100	0	0	0	0	0	10	100	100	100	100	99.94	82.18	100	100	100	100	100
100	0	0	0	0	0	7	100	100	100	0	0	0	100	100	100	100	100
100	0	0	0	0	0	8	100	100	100	0	0	82.18	100	100	100	100	100
100	0	0	0	0	0	10	100	100	100	100	100	100	100	100	100	100	100
0	0	0	0	0	0	10	100	100	100	100	100	81.94	100	100	100	100	100
0	0	100	0	0	0	10	100	100	100	100	100	80.85	100	100	100	100	100
100	0	0	0	0	0	10	100	100	100	100	100	84.85	100	100	100	100	100
100	0	0	0	0	0	10	100	100	100	100	100	100	100	100	100	100	100
100	0	0	0	0	0	10	100	100	100	100	100	81.58	100	100	100	100	100
100	0	0	0	0	0	10	100	100	100	100	85.57	100	100	100	100	100	100
100	0	0	0	0	0	9	100	100	100	0	100	81.7	100	100	100	100	100
100	0	0	0	0	0	10	100	100	100	100	100	80.24	100	100	100	100	100
0	0	100	0	0	0	10	100	100	100	100	100	100	100	100	100	100	100
100	0	0	0	0	0	10	100	100	100	100	100	100	100	100	100	100	100
100	0	0	0	0	0	10	100	100	100	100	100	81.33	100	100	100	100	100
100	0	0	0	0	0	10	100	100	100	100	100	82.67	100	100	100	100	100
0	0	100	0	0	0	10	100	100	100	100	100	100	100	100	100	100	100
0	0	0	0	0	0	10	100	100	100	100	100	80.36	100	100	100	100	100
100	0	0	0	0	0	10	100	100	100	100	100	100	100	100	100	100	100
100	0	0	0	0	0	10	100	100	100	100	100	84	100	100	100	100	100
100	0	0	0	0	0	10	100	100	100	100	85.05	80.12	100	100	100	100	100
100	0	0	0	0	0	9	100	100	100	0	88.76	100	100	100	100	100	100
0	0	100	0	0	0	7	100	100	100	0	0	0	100	100	100	100	100
0	0	100	0	0	0	10	100	100	100	100	99.94	100	100	100	100	100	100
100	0	0	0	0	0	10	100	100	100	100	100	100	100	100	100	100	100
100	0	0	0	0	0	9	100	100	100	0	85.05	100	100	100	100	100	100
0	0	100	0	0	0	10	100	100	100	100	100	100	100	100	100	100	100
100	0	0	0	0	0	10	100	100	100	100	100	100	100	100	100	100	100
100	0	0	0	0	0	10	100	100	100	100	100	100	100	100	100	100	100
100	0	0	0	0	0	10	100	100	100	100	100	100	100	100	100	100	100
100	0	0	0	0	0	10	100	100	100	100	100	100	100	100	100	100	100
100	0	0	0	0	0	8	100	100	100	0	0	100	100	100	100	100	100
100	0	0	0	0	0	10	100	100	100	100	100	100	100	100	100	100	100
0	0	100	0	0	0	10	100	100	100	100	100	100	100	100	100	100	100
100	0	0	0	0	0	10	100	100	100	100	99.88	100	100	100	100	100	100
100	0	0	0	0	0	10	100	100	100	100	100	81.33	100	100	100	100	100
100	0	0	0	0	0	7	100	100	100	0	0	0	100	100	100	100	100
100	0	0	0	0	0	8	100	100	100	0	0	81.94	100	100	100	100	100
100	0	0	0	0	0	10	100	100	100	100	100	81.45	100	100	100	100	100
0	0	0	0	0	0	9	100	100	100	0	100	82.67	100	100	100	100	100
100	0	0	0	0	0	10	100	100	100	100	100	100	100	100	100	100	100
100	0	0	0	0	0	10	100	100	100	100	100	100	100	100	100	100	100
100	0	0	0	0	0	10	100	100	100	100	100	100	100	100	100	100	100
100	0	0	0	0	0	8	100	100	100	0	0	100	100	100	100	100	100
100	0	0	0	0	0	10	100	100	100	100	84.93	81.33	100	100	100	100	100
100	0	0	0	0	0	10	100	100	100	100	100	100	100	100	100	100	100
100	0	0	0	0	0	10	100	100	100	100	100	100	100	100	100	100	100
0	0	100	0	0	0	10	100	100	100	100	100	90.42	100	100	100	100	100
100	0	0	0	0	0	8	100	100	100	0	0	90.18	100	100	100	100	100
100	0	0	0	0	0	10	100	100	100	100	100	100	100	100	100	100	100
100	0	0	0	0	0	10	100	100	100	100	100	100	100	100	100	100	100
100	0	0	0	0	0	10	100	100	100	100	100	100	100	100	100	100	100
100	0	0	0	0	0	10	100	100	100	100	100	100	100	100	100	100	100
100	0	0	0	0	0	10	100	100	100	100	100	100	100	100	100	100	100
0	0	0	0	0	0	10	100	100	100	100	100	100	100	100	100	100	100
100	0	0	0	0	0	10	100	100	100	100	100	100	100	100	100	100	100
100	0	0	0	0	0	10	100	100	100	100	100	100	100	100	100	100	100
0	0	0	0	0	0	10	100	100	100	100	100	100	100	100	100	100	100
0	0	0	0	0	0	8	100	100	100	0	0	100	100	100	100	100	100

100	0	0	0	0	0	10	100	100	100	100	100	100	100	100	100	100	100
100	0	0	0	0	0	10	100	100	100	100	100	100	100	100	100	100	100
100	0	0	0	0	0	10	100	100	100	100	85.63	82.67	100	100	100	100	100
0	0	100	0	0	0	8	100	100	100	0	0	100	100	100	100	100	100
100	0	0	0	0	0	10	100	100	100	100	100	100	100	100	100	100	100
100	0	0	0	0	0	10	100	100	100	100	85.63	100	100	100	100	100	100
0	0	0	0	0	0	10	100	100	100	100	100	100	100	100	100	100	100
100	0	0	0	0	0	10	100	100	100	100	99.94	100	100	100	100	100	100
100	0	0	0	0	0	10	100	100	100	100	100	100	100	100	100	100	100
100	0	0	0	0	0	9	100	100	100	0	100	100	100	100	100	100	100
100	0	0	0	0	0	8	100	100	100	0	0	100	100	100	100	100	100
100	0	0	0	0	0	10	100	100	100	100	100	100	100	100	100	100	100
100	0	0	0	0	0	10	100	100	100	100	100	100	100	100	100	100	100
100	0	0	0	0	0	10	100	100	100	100	100	84.97	100	100	100	100	100
100	0	0	0	0	0	9	100	100	100	100	0	83.15	100	100	100	100	100
100	0	0	0	0	0	9	100	100	100	100	0	82.3	100	100	100	100	100
100	0	0	0	0	0	10	100	100	100	100	100	100	100	100	100	100	100
100	0	0	0	0	0	10	100	100	100	100	100	100	100	100	100	100	100
100	0	0	0	0	0	10	100	100	100	100	100	100	100	100	100	100	100
100	0	0	0	0	0	9	100	100	100	100	0	80.85	100	100	100	100	100
100	0	0	0	0	0	10	100	100	100	100	100	100	100	100	100	100	100
100	0	0	0	0	0	10	100	100	100	100	100	100	100	100	100	100	100
100	0	0	0	0	0	10	100	100	100	100	100	100	100	100	100	100	100
100	0	0	0	0	0	9	100	100	100	0	100	82.55	100	100	100	100	100
0	0	0	0	0	0	10	100	100	100	100	100	89.09	100	100	100	100	100
0	0	0	0	0	0	10	100	100	100	100	100	80.36	100	100	100	100	100
0	0	0	0	0	0	10	100	100	100	100	100	80.73	100	100	100	100	100
0	0	0	0	0	0	10	100	100	100	100	100	83.15	100	100	100	100	0
0	0	0	0	0	0	9	100	100	100	0	99.55	100	100	100	100	100	
0	0	0	0	0	0	10	100	100	100	100	100	100	100	100	100	100	

Table S4-A. Marginal likelihood estimates and pairwise differences between BEAST models.

Sample group	Model number	Marginal likelihood	P1-1-A	P1-1-B	P1-1-C	P1-1-D	P1-1-E	P1-1-F	P1-1-G	P1-1-H	P1-1-I	P1-1-J
P1-1	**P1-1-A**	-16602.69	0.00	8.69	22.30	71.75	-55.77	-125.39	-121.23	-107.40	362.78	290.85
	**P1-1-B**	-16611.38	-8.68	0.00	13.62	63.06	-64.46	-134.07	-129.92	-116.09	354.09	282.17
	**P1-1-C**	-16624.99	-22.30	-13.62	0.00	49.45	-78.07	-147.69	-143.53	-129.71	340.48	268.55
	**P1-1-D**	-16674.44	-71.74	-63.06	-49.45	0.00	-127.52	-197.13	-192.98	-179.15	291.03	219.11
	**P1-1-E**	-16546.92	55.78	64.46	78.07	127.52	0.00	-69.61	-65.46	-51.63	418.55	346.63
	**P1-1-F***	-16477.30	125.39	134.07	147.69	197.13	69.61	0.00	4.15	17.98	488.16	416.24
	**P1-1-G**	-16481.46	121.24	129.92	143.53	192.98	65.46	-4.15	0.00	13.83	484.01	412.09
	**P1-1-H**	-16495.29	107.41	116.09	129.71	179.15	51.63	-17.98	-13.83	0.00	470.18	398.26
	**P1-1-I**	-16965.47	-362.77	-354.09	-340.48	-291.03	-418.55	-488.16	-484.01	-470.18	0.00	-71.92
	**P1-1-J**	-16893.54	-290.85	-282.17	-268.55	-219.11	-346.63	-416.24	-412.09	-398.26	71.92	0.00
	**P1-1-K**	-16899.42	-296.73	-288.05	-274.43	-224.98	-352.50	-422.12	-417.96	-404.14	66.05	-5.88
	**P1-1-L**	-16914.18	-311.49	-302.81	-289.19	-239.74	-367.26	-436.88	-432.72	-418.89	51.29	-20.64
	**P1-1-M**	-16759.93	-157.23	-148.55	-134.94	-85.49	-213.01	-282.62	-278.47	-264.64	205.54	133.62
	**P1-1-N**	-16695.87	-93.18	-84.50	-70.88	-21.44	-148.96	-218.57	-214.42	-200.59	269.59	197.67
	**P1-1-O**	-16694.99	-92.29	-83.61	-70.00	-20.55	-148.07	-217.68	-213.53	-199.70	270.48	198.56
	**P1-1-P**	-16708.11	-105.41	-96.73	-83.11	-33.67	-161.19	-230.80	-226.65	-212.82	257.36	185.44
P1-2	**P1-2-A**	-10243.92	0.00	7.81	15.73	-28.11	49.44	-77.44	-69.37	-61.02	279.26	229.10
	**P1-2-B**	-10251.73	-7.81	0.00	7.92	-35.92	41.63	-85.26	-77.18	-68.84	271.45	221.29
	**P1-2-C**	-10259.66	-15.73	-7.92	0.00	-43.84	33.70	-93.18	-85.10	-76.76	263.53	213.37
	**P1-2-D**	-10215.81	28.11	35.92	43.84	0.00	77.55	-49.33	-41.26	-32.91	307.37	257.21
	**P1-2-E**	-10293.36	-49.44	-41.63	-33.70	-77.55	0.00	-126.88	-118.81	-110.46	229.82	179.66
	**P1-2-F***	-10166.48	77.44	85.26	93.18	49.33	126.88	0.00	8.08	16.42	356.71	306.55
	**P1-2-G**	-10174.55	69.37	77.18	85.10	41.26	118.81	-8.08	0.00	8.34	348.63	298.47
	**P1-2-H**	-10182.90	61.02	68.84	76.76	32.91	110.46	-16.42	-8.34	0.00	340.29	290.13
	**P1-2-I**	-10523.18	-279.26	-271.45	-263.53	-307.37	-229.82	-356.71	-348.63	-340.29	0.00	-50.16
	**P1-2-J**	-10473.02	-229.10	-221.29	-213.37	-257.21	-179.66	-306.55	-298.47	-290.13	50.16	0.00
	**P1-2-K**	-10481.98	-238.06	-230.25	-222.32	-266.17	-188.62	-315.50	-307.43	-299.08	41.20	-8.96
	**P1-2-L**	-10488.41	-244.49	-236.68	-228.76	-272.60	-195.05	-321.94	-313.86	-305.52	34.77	-15.39
	**P1-2-M**	-10383.50	-139.57	-131.76	-123.84	-167.68	-90.14	-217.02	-208.94	-200.60	139.69	89.53
	**P1-2-N**	-10332.83	-88.91	-81.10	-73.17	-117.02	-39.47	-166.35	-158.28	-149.93	190.35	140.19
	**P1-2-O**	-10339.88	-95.96	-88.15	-80.22	-124.07	-46.52	-173.40	-165.33	-156.98	183.30	133.14
	**P1-2-P**	-10348.83	-104.91	-97.10	-89.18	-133.02	-55.47	-182.36	-174.28	-165.94	174.35	124.19

*The difference between models were calculated as log Bayes Factor.The combination of a Bayesian skyline coalescent prior, an uncorrelated log-normal relaxed clock model with exponential rate variation, and a GTR+Γ substitution model with estimated base frequency yielded the highest log marginal likelihood , indicating it as the best-fitting model for our dataset.

P1-1-K	P1-1-L	P1-1-M	P1-1-N	P1-1-O	P1-1-P
296.73	311.49	157.24	93.18	92.30	105.42
288.05	302.81	148.55	84.50	83.61	96.73
274.43	289.19	134.94	70.88	70.00	83.11
224.98	239.74	85.49	21.44	20.55	33.67
352.50	367.26	213.01	148.96	148.07	161.19
422.12	436.88	282.62	218.57	217.68	230.80
417.96	432.72	278.47	214.42	213.53	226.65
404.14	418.89	264.64	200.59	199.70	212.82
-66.05	-51.29	-205.54	-269.59	-270.48	-257.36
5.88	20.64	-133.62	-197.67	-198.56	-185.44
0.00	14.76	-139.49	-203.55	-204.43	-191.32
-14.76	0.00	-154.25	-218.31	-219.19	-206.07
139.49	154.25	0.00	-64.05	-64.94	-51.82
203.55	218.31	64.05	0.00	-0.89	12.23
204.43	219.19	64.94	0.89	0.00	13.12
191.32	206.07	51.82	-12.23	-13.12	0.00
238.06	244.49	139.57	88.91	95.96	104.91
230.25	236.68	131.76	81.10	88.15	97.10
222.32	228.76	123.84	73.17	80.22	89.18
266.17	272.60	167.68	117.02	124.07	133.02
188.62	195.05	90.14	39.47	46.52	55.47
315.50	321.94	217.02	166.35	173.40	182.36
307.43	313.86	208.94	158.28	165.33	174.28
299.08	305.52	200.60	149.93	156.98	165.94
-41.20	-34.77	-139.69	-190.35	-183.30	-174.35
8.96	15.39	-89.53	-140.19	-133.14	-124.19
0.00	6.43	-98.48	-149.15	-142.10	-133.15
-6.43	0.00	-104.92	-155.58	-148.53	-139.58
98.48	104.92	0.00	-50.66	-43.62	-34.66
149.15	155.58	50.66	0.00	7.05	16.00
142.10	148.53	43.62	-7.05	0.00	8.95
133.15	139.58	34.66	-16.00	-8.95	0.00

Table S4-B. Parameter configurations of corresponding BEAST models.

Sample group	Tree prior	Substitution model	Base frequency	Site heterogeneity	Clock type	Relaxed distribution	Model number	Marginal likelihood	Effective sample size of ML	Root age	Effective sample size of root age
P1-1	Bayesian skyline	GTR	Empirical	Gamma	uncorrelated relaxed clock	Exponential	P1-1-B	-16602.69	412	1886.20	212.00
	Bayesian skyline	GTR	Empirical	Gamma	uncorrelated relaxed clock	Gamma	P1-1-C	-16611.38	147.00	1884.982	145.00
	Bayesian skyline	GTR	Empirical	Gamma	uncorrelated relaxed clock	Lognormal	P1-1-D	-16624.99	277.00	1874.867	210.00
	Bayesian skyline	GTR	Empirical	Gamma	strict clock	-	P1-1-E	-16674.44	987.00	1658.77	450.00
	Bayesian skyline	GTR	Estimate	Gamma	strict clock	-	P1-1-F	-16546.92	1505.00	1663.40	536.00
	Bayesian skyline	GTR	Estimate	Gamma	uncorrelated relaxed clock	Exponential	P1-1-G	-16477.30	520.00	1885.03	156.00
	Bayesian skyline	GTR	Estimate	Gamma	uncorrelated relaxed clock	Gamma	P1-1-H	-16481.46	363.00	1890.95	166.00
	Bayesian skyline	GTR	Estimate	Gamma	uncorrelated relaxed clock	Lognormal	P1-1-I	-16495.29	312.00	1881.22	109.00
	Bayesian skyline	HKY	Empirical	Gamma	strict clock	-	P1-1-J	-16965.47	1445.00	1662.53	503.00
	Bayesian skyline	HKY	Empirical	Gamma	uncorrelated relaxed clock	Exponential	P1-1-K	-16893.54	255.00	1888.31	154.00
	Bayesian skyline	HKY	Empirical	Gamma	uncorrelated relaxed clock	Gamma	P1-1-L	-16899.42	388.00	1887.14	118.00
	Bayesian skyline	HKY	Empirical	Gamma	uncorrelated relaxed clock	Lognormal	P1-1-M	-16914.18	1202	1881.51	108
	Bayesian skyline	HKY	Estimate	Gamma	strict clock	-	P1-1-N	-16759.93	1164.00	1661.88	567.00
	Bayesian skyline	HKY	Estimate	Gamma	uncorrelated relaxed clock	Exponential	P1-1-O	-16695.87	147.00	1889.92	208.00
	Bayesian skyline	HKY	Estimate	Gamma	uncorrelated relaxed clock	Gamma	P1-1-P	-16694.99	428.00	1885.55	157.00
	Bayesian skyline	HKY	Estimate	Gamma	uncorrelated relaxed clock	Lognormal	P1-1-Q	-16708.11	1164	1869.30	224
P1-2	Bayesian skyline	GTR	Empirical	Gamma	uncorrelated relaxed clock	Exponential	P1-2-B	-10243.92	1594.00	1854.26	128.00
	Bayesian skyline	GTR	Empirical	Gamma	uncorrelated relaxed clock	Gamma	P1-2-C	-10251.73	1578.00	1842.91	144.00
	Bayesian skyline	GTR	Empirical	Gamma	uncorrelated relaxed clock	Lognormal	P1-2-D	-10259.66	1766	1765.145	452
	Bayesian skyline	GTR	Empirical	Gamma	strict clock	-	P1-2-E	-10215.81	1659	1748.943	2081
	Bayesian skyline	GTR	Estimate	Gamma	strict clock	-	P1-2-F	-10293.36	1978.00	1744.21	1767.00
	Bayesian skyline	GTR	Estimate	Gamma	uncorrelated relaxed clock	Exponential	P1-2-G	-10166.48	1179.00	1851.79	199.00
	Bayesian skyline	GTR	Estimate	Gamma	uncorrelated relaxed clock	Gamma	P1-2-H	-10174.55	1189	1841.586	258
	Bayesian skyline	GTR	Estimate	Gamma	uncorrelated relaxed clock	Lognormal	P1-2-I	-10182.90	1551.00	1774.97	203.00
	Bayesian skyline	HKY	Empirical	Gamma	strict clock	-	P1-2-J	-10523.18	3149.00	1743.49	1679.00
	Bayesian skyline	HKY	Empirical	Gamma	uncorrelated relaxed clock	Exponential	P1-2-K	-10473.02	1211.00	1857.78	153.00
	Bayesian skyline	HKY	Empirical	Gamma	uncorrelated relaxed clock	Gamma	P1-2-L	-10481.98	761.00	1836.71	370.00
	Bayesian skyline	HKY	Empirical	Gamma	uncorrelated relaxed clock	Lognormal	P1-2-M	-10488.41	2089.00	1764.22	924.00
	Bayesian skyline	HKY	Estimate	Gamma	strict clock	-	P1-2-N	-10383.50	2368.00	1750.63	1772.00
	Bayesian skyline	HKY	Estimate	Gamma	uncorrelated relaxed clock	Exponential	P1-2-O	-10332.83	1201.00	1846.60	127.00
	Bayesian skyline	HKY	Estimate	Gamma	uncorrelated relaxed clock	Gamma	P1-2-P	-10339.88	1296.00	1835.39	163.00
	Bayesian skyline	HKY	Estimate	Gamma	uncorrelated relaxed clock	Lognormal	P1-2-Q	-10348.83	1916.00	1770.79	937.00

Table S5. The accessory genes identified in this study that were associated with *M. pneumoniae* P1 genotypes.

Genes	Number of genes presented in P1-1 lineage	Number of genes presented in P1-2 lineage	Number of genes absent from P1-1 lineage	Number of genes absent from P1-2 lineage	Chisq	P value	Best-match NCBI reference sequence	Percentage of identical match	Corresponding gene annotation
**P1-1 Lineage**									
group_1316	427	0	0	135	556.534232	4.77E-123			
group_1328	427	0	0	135	556.534232	4.77E-123			
group_1529	427	0	0	135	556.534232	4.77E-123			
group_1530	427	0	0	135	556.534232	4.77E-123			
group_2425	427	0	0	135	556.534232	4.77E-123	NP_110340_1	100	PTS system mannitol-specific transporter subunit
group_2525	427	0	0	135	556.534232	4.77E-123			
group_2745	427	0	0	135	556.534232	4.77E-123			
group_2320	426	0	1	135	551.135618	7.13E-122			
group_534	426	0	1	135	551.135618	7.13E-122			
smc_2	426	0	1	135	551.135618	7.13E-122			
group_728	427	1	0	134	551.080782	7.33E-122			
group_2671	425	0	2	135	545.815816	1.02E-120			
group_436	425	0	2	135	545.815816	1.02E-120			
group_1318	426	1	1	134	545.682823	1.09E-120			
leuS_3	427	2	0	133	545.652757	1.11E-120	NP_110072_1	100	leucine--tRNA ligase
mgpA_37	424	0	3	135	540.573113	1.41E-119			
group_1523	424	0	3	135	540.573113	1.41E-119	NP_109826_1	100	hypothetical protein MPN138
group_2186	423	0	4	135	535.405845	1.88E-118			
arcA_2	424	1	3	134	535.123318	2.17E-118	WP_01426236	51.4	arginine deiminase
group_2433	422	0	5	135	530.312394	2.42E-117			
group_549	423	1	4	134	529.958344	2.88E-117	NP_109724_1	50.3	hypothetical protein MPN036
group_1522	421	0	6	135	525.291192	2.99E-116	NP_109737_1	52.2	hypothetical protein MPN049
group_2362	423	3	4	132	519.140404	6.51E-115			
group_552	423	6	4	129	503.103493	2.01E-111	NP_109724_1	60.4	hypothetical protein MPN036
group_533	417	1	10	134	500.474979	7.49E-111			
mgpA_19	414	0	13	135	492.042692	5.12E-109			

group_529	416	3	11	132	485.040586	1.71E-107			
ruvB_1	427	17	0	118	467.166645	1.33E-103	NP_110225_1	100	Holliday junction ATP-dependent DNA helicase RuvB
**P1-2 Lineage**									
group_1190	0	135	427	0	556.534232	4.77E-123	NP_109826_1	96.2	hypothetical protein MPN138
group_1399	0	135	427	0	556.534232	4.77E-123			
group_1433	0	135	427	0	556.534232	4.77E-123			
group_1435	0	135	427	0	556.534232	4.77E-123			
group_1437	0	135	427	0	556.534232	4.77E-123			
polC_7	0	135	427	0	556.534232	4.77E-123	NP_109722_1	98.5	DNA polymerase III
group_1449	1	135	426	0	551.135618	7.13E-122	NP_109737_1	39.3	hypothetical protein MPN049
group_543	1	135	426	0	551.135618	7.13E-122	NP_110260_1	99.7	ABC transporter ATP-binding protein
group_1106	0	134	427	1	551.080782	7.33E-122			
group_1313	0	134	427	1	551.080782	7.33E-122			
group_1410	0	134	427	1	551.080782	7.33E-122			
group_1411	0	134	427	1	551.080782	7.33E-122			
group_1413	0	134	427	1	551.080782	7.33E-122			
group_1415	0	134	427	1	551.080782	7.33E-122			
group_1416	0	134	427	1	551.080782	7.33E-122			
group_1419	0	134	427	1	551.080782	7.33E-122			
group_1420	0	134	427	1	551.080782	7.33E-122			
group_1421	0	134	427	1	551.080782	7.33E-122			
group_1422	0	134	427	1	551.080782	7.33E-122			
group_1423	0	134	427	1	551.080782	7.33E-122			
group_1436	0	134	427	1	551.080782	7.33E-122			
group_1506	0	134	427	1	551.080782	7.33E-122	WP_095532023_1	52.1	ornithine carbamoyltransferase
group_759	2	135	425	0	545.815816	1.02E-120			
group_1107	1	134	426	1	545.682823	1.09E-120			
group_1412	1	134	426	1	545.682823	1.09E-120			
group_1418	1	134	426	1	545.682823	1.09E-120			
group_992	1	134	426	1	545.682823	1.09E-120	NP_110072_1	99.6	leucine--tRNA ligase
group_1417	0	133	427	2	545.652757	1.11E-120			
group_1116	3	135	424	0	540.573113	1.41E-119			

group_609	1	133	426	2	540.255522	1.66E-119			
group_1478	0	132	427	3	540.24998	1.66E-119			
group_702	0	132	427	3	540.24998	1.66E-119	WP_01426236	51.4	arginine deiminase
group_880	0	132	427	3	540.24998	1.66E-119			
group_1123	5	135	422	0	530.312394	2.42E-117			
group_1434	5	135	422	0	530.312394	2.42E-117			
group_883	5	135	422	0	530.312394	2.42E-117			
group_433	3	132	424	3	524.300512	4.91E-116			
group_551	1	129	426	6	518.797716	7.73E-115	NP_109724_1	53.6	hypothetical protein MPN036
group_2361	5	132	422	3	514.055634	8.31E-114			
group_528	9	134	418	1	505.217056	6.96E-112			
group_1414	0	124	427	11	497.91595	2.70E-110			
group_1439	18	134	409	1	464.784661	4.37E-103			
group_531	1	118	426	17	461.788043	1.96E-102			

SUPPLEMENTARY FIGURES

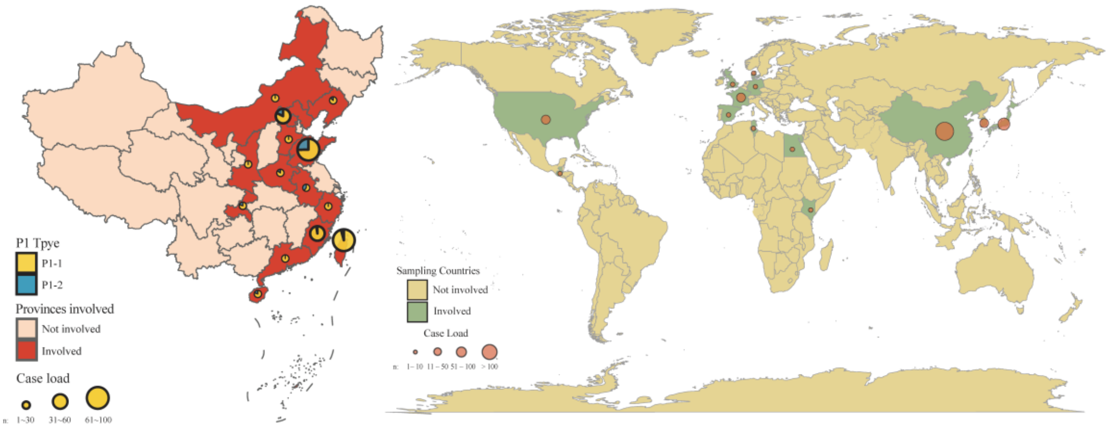

Figure S1. Distribution of the 562 global *M. pneumoniae* strains and regional differences in P1 genotypes used in this study.

(A) Regional distribution of the 271 Chinese *M. pneumoniae* strains identified in this study. The P1 genotype is indicated in different colors. (B) Geographic distribution of the 291 *M. pneumoniae* strains from the NCBI. The size of each circle represents the number of strains.

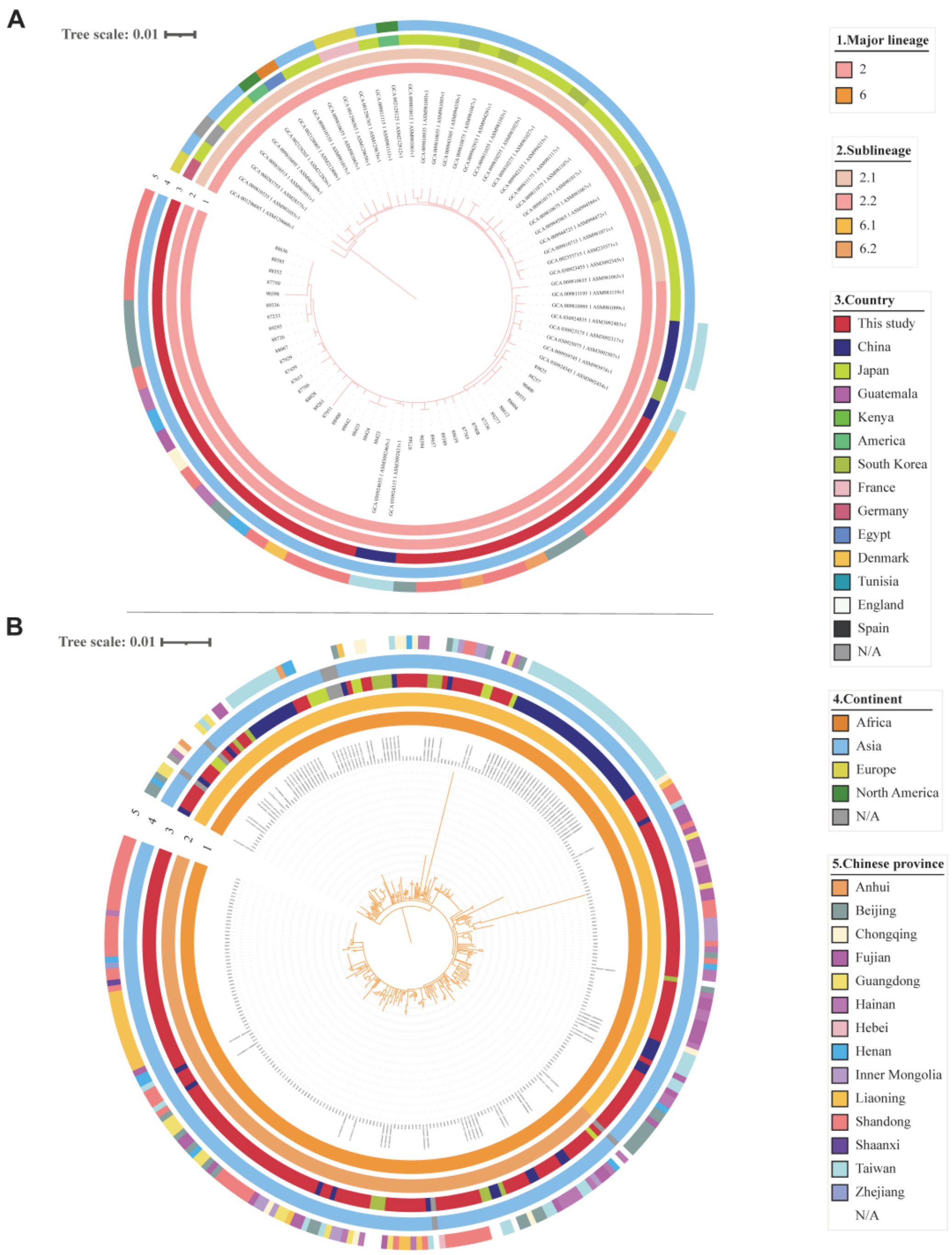

Figure S2. Phylogeny of the two lineages (L2 and L6) of *M. pneumoniae*.

The Chinese isolates identified in this study were mainly distributed in lineages L2 and L6, forming two main Chinese clades. (A) Maximum likelihood phylogentic tree of the *M. pneumoniae* lineage L2. The P1 genotype is indicated in different colors. (B) Maximum likelihood phylogentic tree of the *M. pneumoniae* lineage L6. The rings, from inner to outer, labeled with different colors indicate the lineages, sublineages, continent, country, and Chinese province.

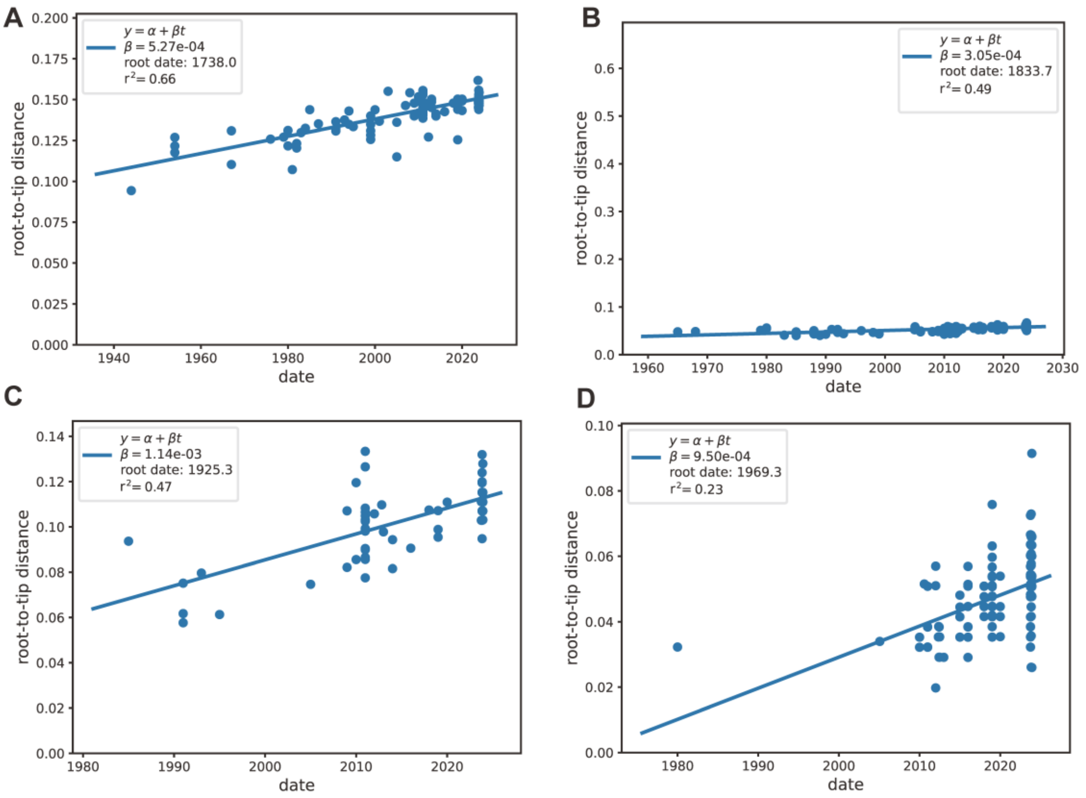

Figure S3. Root-to-tip regression curves demonstrating temporal signal for P1 genotype and L2/L6 lineages following outlier removal.

(A) Root-to-tip regression curve for P1-1 genotype isolates. A linear model ($y=\alpha+\beta t$) was fitted. The blue line represents the regression fit. (B) Root-to-tip regression curve for P1-2 genotype isolates. A linear model ($y=\alpha+\beta t$) was fitted. The blue line represents the regression fit. (C) Root-to-tip regression curve for L2 isolates. A linear model ($y=\alpha+\beta t$) was fitted. The blue line represents the regression fit. (D) Root-to-tip regression curve for L6 isolates. A linear model ($y=\alpha+\beta t$) was fitted. The blue line represents the regression fit.

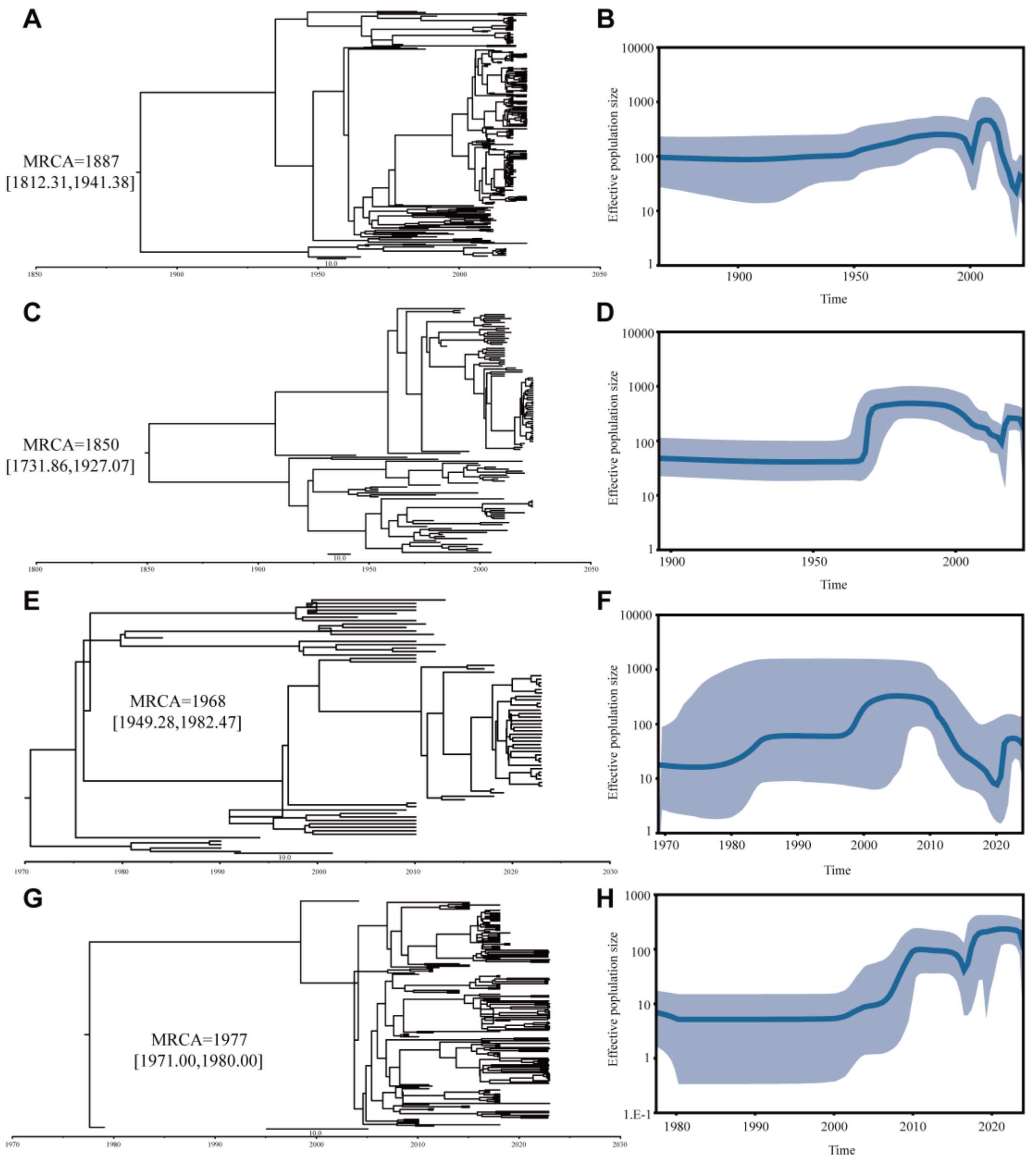

Figure S4. Bayesian phylogenetic tree and estimation of demographic history of P1 genotype and L2/L6 lineages.

(A) The maximum-clade credibility tree of P1-1 genotype isolates. (B) Bayesian skyline plot of P1-1 genotype calculated and visualized using Tracer with blue line indicating the median of the effective population sizes and blue shade delineating the range of 95% highest posterior density interval. (C) The maximum-clade credibility tree of P1-2 genotype isolates. (D) Bayesian skyline plot of P1-2 genotype calculated and visualized using Tracer with blue line indicating the median of the effective population sizes and blue shade delineating the range of 95% highest posterior density interval. (E) The maximum-clade credibility tree of lineage L2 isolates. (F) Bayesian skyline plot of lineage L2 calculated and visualized using Tracer with blue line indicating the median of the effective population sizes and blue shade delineating the range of 95% highest posterior density interval. (G) The maximum-clade credibility tree of lineage L6 isolates. (H) Bayesian skyline plot

of lineage L6 calculated and visualized using Tracer with blue line indicating the median of the effective population sizes and blue shade delineating the range of 95% highest posterior density interval.

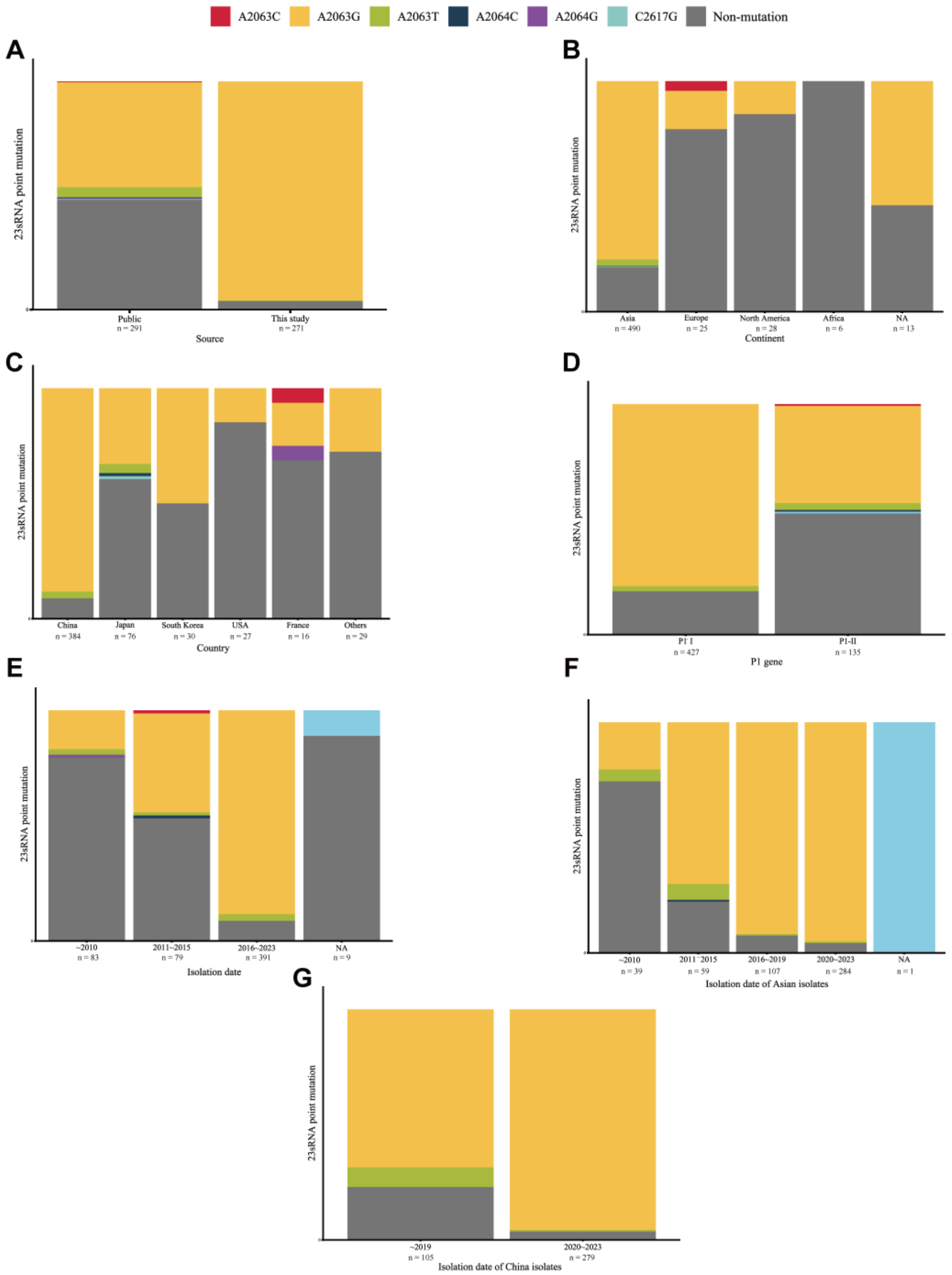

Figure S5. The proportion of global *M. pneumoniae* strains carrying the point mutations of 23S rRNA.

(A) Proportion of the point mutations of 23S rRNA associated with macrolide resistance among the global *M. pneumoniae* collections. (B) Proportion of the point mutations of 23S rRNA associated with macrolide resistance among the *M. pneumoniae* collections from different continents. (C) Proportion of the point mutations of 23S rRNA associated with macrolide resistance among the *M.*

pneumoniae collections from different countries. (D) Proportion of the point mutations of 23S rRNA associated with macrolide resistance among the different P1 genotypes of *M. pneumoniae*. (E) Temporal distribution of the point mutations of 23S rRNA associated with macrolide resistance among the global *M. pneumoniae* collections. (F) Temporal distribution of the point mutations of 23S rRNA associated with macrolide resistance among the Asian *M. pneumoniae* collections. (G) Temporal distribution of the point mutations of 23S rRNA associated with macrolide resistance among the Chinese *M. pneumoniae* collections.

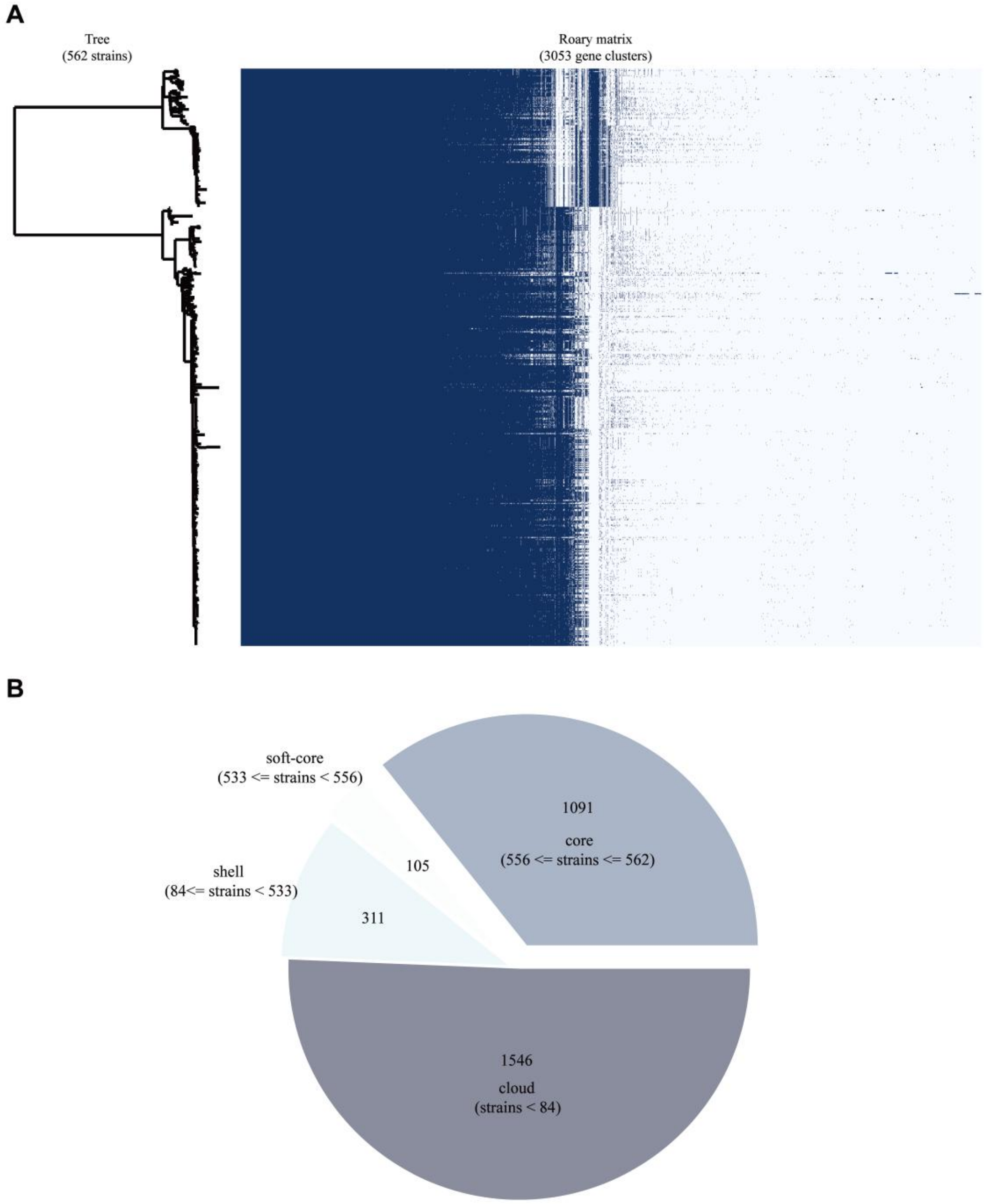

Figure S6. Pangenome analysis of the global *M. pneumoniae* strains.

A total of 3,053 genes were identified by pangenome analysis based on the 562 isolates, including 1,091 core genes, 105 soft core genes, 311 shell genes and 1,546 cloud genes. (A) shows a heatmap

displaying the distribution of the pangenome among isolates. Dark blue indicates the presence of a gene, and light blue indicates the absence of a gene. (B) shows the proportions of core and accessory genes.
